# Capturing carbon monoxide—Accessing bis(boryl)ketones through direct B–B bond carbonylation

**DOI:** 10.1126/sciadv.adz3520

**Published:** 2025-08-27

**Authors:** Eva Beck, Ivo Krummenacher, Thomas Kupfer, Maximilian Dietz, Holger Braunschweig

**Affiliations:** ^1^ Institut für Anorganische Chemie, Julius-Maximilians-Universität Würzburg, Am Hubland, 97074 Würzburg, Germany.; ^2^ Institute for Sustainable Chemistry and Catalysis with Boron, Julius-Maximilians-Universität Würzburg, Am Hubland, 97074 Würzburg, Germany.

## Abstract

Carbon monoxide (CO) readily attacks the B–B bonds of the cyclic tetra(amino)tetraborane B_4_(NCy_2_)_4_ [cyclohexyl (Cy)], which led to insertion and ring expansion, creating tetraboron analogs of cyclopentanone and cyclohexane-1,3-dione. These intriguing molecules are rare instances of stable CO diborylation products that were made accessible by direct CO capture. While the monocarbonyl product shows remarkable thermal stability, the dicarbonyl product rearranges into a bicyclic tetraborylethylene structure under thermal stress. Combined analytical and theoretical efforts clearly highlight the unique electronic, spectroscopic, and structural characteristics of these carbonyl compounds. The cyclopentanone analog engages in stepwise one- and two-electron reduction processes to yield radical monoanion and dianion species while preserving the five-membered ring structure and the boron-boron bonds. Together, these boron heterocycles expand the limited class of stable boryl ketones containing direct boron-carbonyl carbon bonds, thus offering valuable opportunities for exploring the fundamental chemistry and synthetic applications of this functionality.

## Teaser

Using carbon monoxide gas as a selective method for the direct production of bis(boryl)ketones.

## INTRODUCTION

The peculiar electronic nature of the boron-boron bond makes diboranes(4) differ from their carbon-based counterparts on a fundamental level (*1*–*5*). While C–C bonds in alkanes show high bond strengths, which impose notable thermodynamic and kinetic barriers to activation (*6*–*8*), B–B bonds in diboranes(4) and related compounds much more easily engage in functionalization reactions, particularly those involving B–B bond cleavage, eventually forming thermodynamically more stable boron-carbon and boron-heteroatom bonds (*1*–*3*). Tetrachlorodiborane (B_2_Cl_4_), the first reported molecule with a B–B single bond, exemplifies this reactivity by readily adding across carbon-carbon double and triple bonds to create diborylated species (*9*, *10*). The high versatility of the boron-carbon bond in the resulting organoboranes makes them invaluable tools in organic synthesis (*11*, *12*). From an application-related viewpoint, air-stable tetraalkoxy diboron(4) derivatives such as B_2_pin_2_ and B_2_cat_2_ [pinacolato (pin) = 1,2-O_2_C_2_Me_4_ and catecholato (cat) = 1,2-O_2_C_6_H_4_] are preferred (*13*–*15*), despite them requiring metal catalysts for B–B bond activation, unlike the more reactive B_2_Cl_4_ (*1*–*3*, *16*–*20*).

**Fig. 1.**
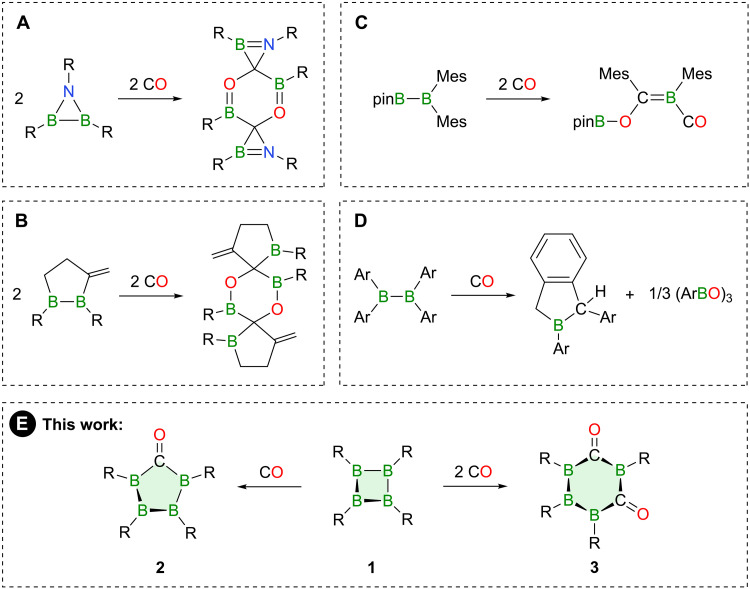
Examples of CO activation by B–B-bonded species. Substituents are as follows: (**A**) R = *t*Bu, (**B**) R = N*i*Pr_2_, (**D**) R = *o*-tolyl, and (**E**) R = NCy_2_.

Alongside their importance in B–C bond formation processes, the remarkable reactivity of B–B bonds also manifests in the surprising ability to activate small molecules such as carbon monoxide (CO), as demonstrated in Fig. 1 for selected examples. Pioneering work in this area was reported by Paetzold and coworkers in 1990 (*21*), the reaction of a *tert*-butyl-substituted azadiborirane with two equivalents of CO to form a tricyclic spiro product and, thus, the transformation of the B–B bond into more stable B–C and B–O bonds (Fig. 1A). The group of Siebert accomplished a similar reactivity for a cyclic organodiborane, which produced a tricyclic structure via the incorporation of two CO molecules (Fig. 1B) (*22*). Closely related diboranes with slightly different ligand environments showed no reactivity toward CO, which highlights the subtle yet critical influence of substituent effects on the boron atoms. These effects are also illustrated by Yamashita’s studies on two distinct diborane(4) derivatives: While the unsymmetric diborane(4) pinB-Bmes_2_ reacts with CO to produce a CO-coordinated boraalkene (Fig. 1C) (*23*), the more electrophilic tetra(*o*-tolyl)diborane(4) induces complete cleavage of the CO triple bond to provide a mixture of boraindane and boroxine (Fig. 1D) (*24*). In all of these cases, products are presumably formed via CO insertion into the B–B bond, generating a transient diboryl ketone through direct carbonylation (*21*–*24*). However, this intermediate remained elusive thus far because of boron’s high oxophilicity, which drives its rapid transformation into further products.

In continuation of our most recent studies covering the efficient synthesis of the cyclic tetra(amino)tetraborane B_4_(NCy_2_)_4_ [**1**, cyclohexyl (Cy)], which is marked by electron-precise B–B bonds, we set out to explore its reactivity toward CO (Fig. 1E) (*25*). We reasoned that **1** might be susceptible to carbonylation forming a ketone structure, with its stability being enhanced by the slightly electron-deficient character of the boron atoms and the steric protection provided by the ligand sphere, eventually hampering further transformations. This line of inquiry was particularly compelling in light of our previous work describing facile chalcogen atom insertion to yield five-membered B_4_E (E = S, Se, and Te) rings (*26*). Herein, we report the synthesis of cyclic ketones **2** and **3** through formal insertion of CO into one and two B–B single bonds of **1**, respectively. These carbonylated products were shown to be stable while displaying unique electronic, spectroscopic, and structural properties. Elevated temperatures are required to cause the thermally induced rearrangement of **2** to a thermodynamically beneficial bicyclic tetraborylethylene structure. These findings not only expand the limited family of stable boryl ketones (*27*–*30*) but also highlight the synthetic value of **1** as a precursor for constructing complex boron-rich molecules.

## RESULTS

### CO insertion

**Fig. 2.**
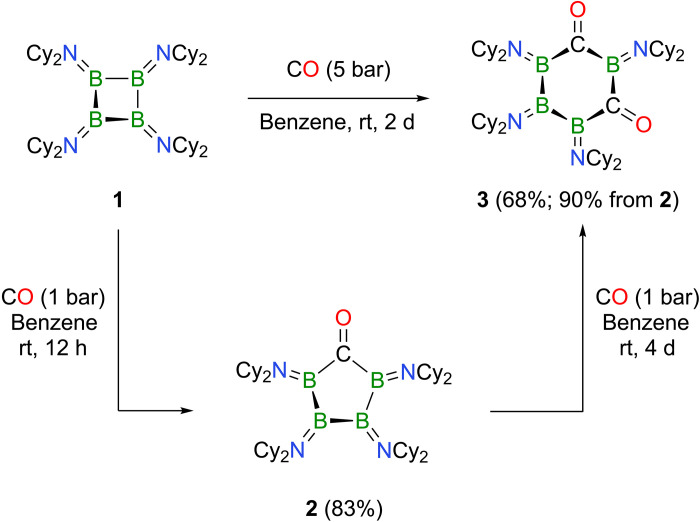
Syntheses of carbonyls 2 and 3. Single and double CO insertions into tetraborane **1** to form **2** and **3**, respectively. rt, room temperature; d, days; h, hours.

Inspired by the discoveries related to CO activation chemistry of B–B-bonded species (*21*–*24*), we turned our own efforts to the reactivity of cyclic tetraborane **1** toward carbon monoxide. Exposure of **1** to 1 bar of CO in benzene solution for 12 hours led to a gradual color change from light blue to dark red and the emergence of two ^11^B nuclear magnetic resonance (NMR) signals at δ = 61.1 and 47.8 parts per million (ppm), consistent with *C*_2_ symmetry of the anticipated insertion product. Following solvent removal, product **2** was isolated as an analytically pure dark red solid in 83% yield (Fig. 2).

**Fig. 3.**
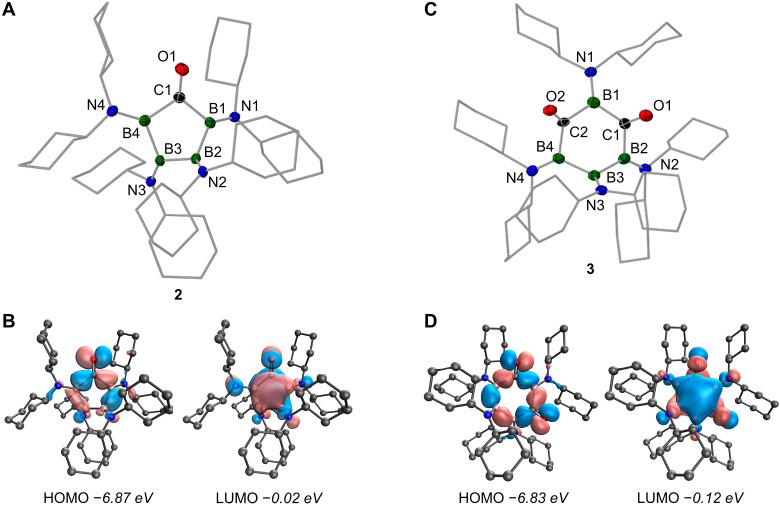
Molecular and electronic structures of carbonylated derivatives of cyclotetraborane 1. (**A** and **C**) Solid state structures of carbonyls **2** and **3**. Displacement ellipsoids are shown at the 50% probability level, and hydrogen atoms and ellipsoids of the cyclohexyl groups are omitted for clarity. (**B** and **D**) HOMO and LUMO of carbonyls **2** and **3** [isosurface plots at 0.04 atomic units (a.u.); SMD-ωB97xd/def2-TZVP//ωB97xd/def2SVPP; calculated eigenvalues in italics].

X-ray diffraction analysis of suitable single crystals clearly verified the formal 1,1-insertion of CO into one of the B–B bonds (Fig. 3A). The central five-membered B_4_C ring adopts an envelope-type conformation, with three of the boron atoms and the carbonyl carbon atom in one plane, while the residual boron atom resides above. The central boron-boron bond [1.703(2) Å] is slightly shorter than the two B–B bonds flanking the carbonyl moiety [1.731(2) and 1.733(2) Å], while the boron-nitrogen bonds [1.397(1) to 1.410(2) Å] remain largely unaltered with respect to the precursor **1**. **2** shows a carbon-oxygen bond length of 1.240(2) Å, characteristic of carbonyl functionalities, and can thus be considered a 2,3,4,5-tetraboracyclopentanone. The carbonyl group itself has only little impact on the average bond length of the adjacent B–C bonds [1.637(3) Å], which are only marginally longer than the approximated 1.60 Å commonly encountered in three-coordinate boron molecules (*31*). The bond angle at the carbonyl carbon in **2** [105.56(*9*)°] is, however, somewhat more acute as in cyclopentanone [108.3(1)°/109.1(1)°] (*32*), which indicates higher angle strain in the geometry of **2**, when keeping the 120° bond angles ideally encountered in carbonyl groups in mind. In solution, the unique electronic environment of the carbonyl carbon becomes manifest in a strongly deshielded ^13^C NMR resonance (δ = 305 ppm) as compared to cyclopentanone (δ = 220 ppm) (*33*) or a related diboryl ketone (δ = 283.1 ppm) from our own group (*34*). Moreover, the carbonyl stretching vibration of **2** is weak and found red shifted at ν = 1578 cm^−1^ in a region atypical for carbonyl species, which clearly contrasts with the two intense carbonyl stretching vibrations at ν = 1984 and 1972 cm^−1^ of our previously reported diboryl-substituted ketone (*34*). The shift to lower energies is also evident in the ultraviolet-visible (UV-vis) absorption spectrum of **2** in benzene at room temperature, which features two moderately intense absorptions at λ_max_ = 736 and 517 nm. Although rather uncommon, such characteristics are not without precedent, and similar spectroscopic profiles have been found for bis(trimethylsilyl) ketone [δ(^13^C) = 318.8 ppm, ν(C=O) = 1570 cm^−1^, and λ_max_ = 533 nm] and related derivatives (*35*–*37*).

Despite their somewhat unexpected nature, the intriguing spectroscopic features of **2** were accurately reproduced by quantum chemical calculations at the SMD-ωB97xd/def2-TZVP//ωB97xd/def2SVPP level of theory. Thus, the frequency of the prominent C=O stretching vibration of **2** showed a value of ν_calc_ = 1586 cm^−1^, while time-dependent density functional theory (TD-DFT) calculations identified the first two excited states at excitation energies of λ_calc_ = 727 and 481 nm with moderate oscillator strengths of *f* = 0.0044 and 0.0190, respectively. These excitations correspond to either HOMO➔LUMO [λ_calc_ = 727 nm; highest occupied molecular orbital (HOMO) and lowest unoccupied molecular orbital (LUMO)] or HOMO-1➔LUMO transitions (λ_calc_ = 481 nm). The calculations clearly showed that (i) HOMO-1 of **2** is dominated by B–B σ-bonding orbitals (Fig. S25), and (ii) its HOMO is a combination of C=O π*- and B–B σ-bonding interactions, while (iii) its LUMO is π-type in nature and delocalized across the whole B_4_C core (Fig. 3B).

Cyclic ketone **2** shows remarkable thermal stability up to temperatures of 80°C, which indicates that CO insertion into **1** is not easily reversible. This stability becomes also manifest in its high resistance to decarbonylation even under prolonged vacuum conditions. **2** is a unique molecule, as it represents the first example for the direct 1,1-diborylation of CO by a B–B-bonded species or, in other words, for the insertion of CO into a B–B bond. While similar carbonylated compounds have been postulated earlier as intermediates in related transformations, they have remained elusive thus far due to their inherent instability and tendency to take part in subsequent rearrangement processes (*21*–*24*). It should be noted here that other stable compounds featuring the B_2_C=O functionality are known but have been generated through capture of CO by boron-based diradicals (*38*) or through alternative routes involving diborenes (*34*, *39*, *40*).

Figure 2 shows that the B_4_ ring of **1** is capable of incorporating not only one but also two molecules of CO via two successive 1,1-diborylation events, ultimately producing the 1,3-diketone **3**. Double CO insertion can be accomplished in two different ways by (i) applying higher CO pressures (5 bar) directly to a solution of tetraborane **1** or by (ii) exposing ketone **2** to 1 bar of CO over extended reaction times of 4 days. In either case, 1,3-diketone **3** is isolated as a pink solid in good to excellent yields of up to 68% or 90%, respectively. We were not able to detect any other double insertion isomers or any higher insertion products, regardless of the reaction conditions. In solution, **3** is characterized by three distinct, broad signals in the ^11^B NMR spectrum at δ = 60.6, 55.6, and 33.1 ppm, while the ^13^C NMR spectrum features a single resonance for the two equivalent carbonyl groups at δ = 301.5 ppm. X-ray diffraction analysis of suitable single crystals helped to establish the tetraboracyclohexane-1,3-dione framework of **3** (Fig. 3C). Thus, the six-membered B_4_C_2_ ring adopts a chair-type conformation, in which the internal angles at carbon [119.8(4)° and 117.1(4)°] closely match the ideal 120° angle of sp^2^-hybridized carbon atoms. The remaining B–B–B, B–B–C, and C–B–C angles range from 102.5(4)° to 110.8(4)°. Carbon-oxygen bond lengths of 1.252(4) and 1.248(6) Å clearly indicate two carbonyl functionalities, while the boron-nitrogen distances [1.393(8) to 1.408(8) Å] and boron-boron bonds [1.724(7) and 1.729(9) Å] are within ranges typically associated with amino-substituted oligoboranes (*41*–*44*). Other spectroscopic features of 1,3-diketone **3** are reminiscent of those mentioned above for ketone **2**, i.e., weak carbonyl stretching vibrations at ν = 1548 and 1522 cm^−1^ in its solid-state Fourier transform infrared (FTIR) spectrum and two defining maxima at λ_max_ = 731 and 522 nm in the solution UV-vis absorption spectrum, with the lowest-energy absorption being rather broad and of low intensity. (TD-)DFT calculations agree very well with these results: two distinct vibrations at ν_calc_ = 1565 and 1539 cm^−1^ for the symmetric and asymmetric C=O stretching modes, and lowest-energy excited states at transition energies of λ_max_ = 688 and 485 nm with very low or moderate oscillator strengths of *f* = 0.0001 and 0.0532, respectively. Unlike for **2**, the first two excitations of **3** are not made up of only one single transition each but are more complex and involve combinations of several transitions [λ_max_ = 688 nm: HOMO-1➔LUMO+2 and HOMO➔LUMO; λ_max_ = 485 nm: HOMO-3➔LUMO, HOMO-1➔LUMO, HOMO➔LUMO+1, and HOMO➔LUMO+2; see Fig. 3D and fig. S28 for graphical representations of relevant molecular orbitals (MOs)]. We note, however, that the basic composition of the involved MOs is somewhat similar to those relevant in the electronic excitation of **2**. We also used quantum chemistry to contemplate why we were only able to observe the 1,3-isomer of diketone **3** and studied the thermochemistry of the 1,2, 1,3-, and 1,4-isomers relative to each other. In line with our observations, the calculations show that the 1,3-diketone **3**_1,3_ is the preferred isomer, while the other isomers are close yet slightly higher in energy by Δ*E*_298_ = +3.4 kcal/mol (**3**_1,2_) and Δ*E*_298_ = +4.4 kcal/mol (**3**_1,4_). However, the energy differences are too small to provide a reliable explanation for the 1,3-pattern of the insertion process; a definite explanation eludes us thus far.

**Fig. 4.**
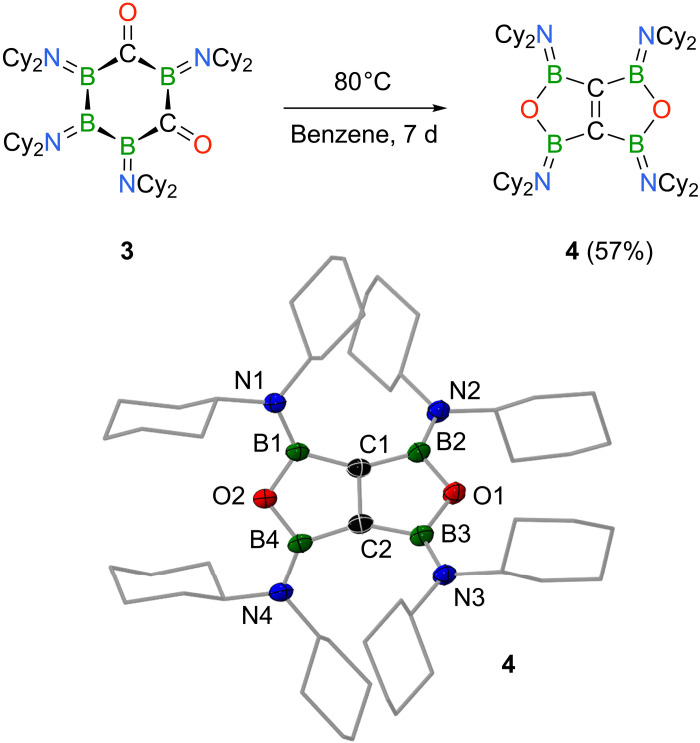
Synthesis and molecular structure of 4. Thermal rearrangement of **3** to form bicyclic tetraborylethylene derivative **4**. Molecular structure of **4** with displacement ellipsoids was shown at the 50% probability level (hydrogen atoms and ellipsoids of the cyclohexyl groups are omitted for clarity). d, days.

As described above, both carbonyl compounds **2** and **3** are quite resistant to thermal stress, and the fact that such structures are generated in the first place was particularly intriguing to us, as thermodynamics should favor the formation of B–O bonds over that of B–C bonds during the CO insertion process (see Fig. 1 for literature-known examples) (*21*–*24*). With this in mind, we next studied the temperature resistances of **2** and **3** in more detail. While carbonyl **2** is stable up to 80°C, dicarbonyl **3** is forced into isomerization under identical conditions, which led to the gradual transformation into a different species with a ^11^B NMR resonance at δ = 34.2 ppm. Reaction progress is readily made visible by a distinct color change from pink to yellow and reaches completion within 1 week, after which product **4** was isolated as a colorless solid in 57% yield (Fig. 4). NMR spectroscopic data of **4** indicated a highly symmetric molecule, for which reason its identity initially remained unclear, and it required a single-crystal x-ray diffraction study to establish its bicyclic structure with a central carbon-carbon double bond (Fig. 4). Evidently, **4** is made up of two five-membered oxadiborolane rings, fused across the two carbon atoms, with the diboroxane (BOB) groups arranged in a facially opposed, Janus-type fashion. The bicyclic system adopts a nonplanar, twisted conformation around the C=C double bond, presumably because of steric pressure inflicted by the dicyclohexylamino substituents, as suggested by B–C–C–B dihedral angles of 23.4(2)° and 22.0(2)°. The strained geometry in combination with conjugation effects spanning the C=C π-bond and the four boron p orbitals cause an elongation of the carbon-carbon bond to 1.391(3) Å (compared with 1.34 Å for a typical C=C bond). Similarly, ^13^C NMR spectroscopy is in line with mirror symmetry in solution as well, and a single resonance is present for the olefinic carbon atoms of **4** at δ = 190.9 ppm. All structural and spectroscopic features of the central C_2_B_4_ moiety of **4** align well with those of a related tetraborylethylene derivative recently reported by Kusumoto and coworkers [δ(^13^C) = 196 ppm; C=C 1.368(2) Å], while its bicyclic structure is unique to **4** (*45*). The structural motif of a five-membered oxadiborolane ring with a carbon-carbon double bond, on the other hand, was first described by Köster *et al*. in 1994 (*46*).

### Redox chemistry

**Fig. 5.**
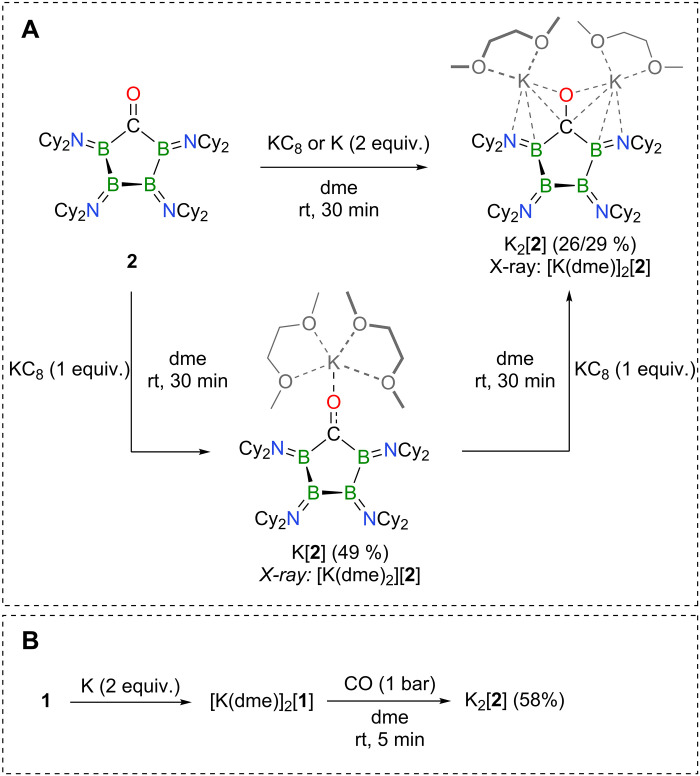
Reduction chemistry of 2. (**A**) Synthesis of mono- and dianions of **2**. (**B**) Alternative route to the dianion from tetraborane **1**. rt, room temperature; min, minutes.

**Fig. 6.**
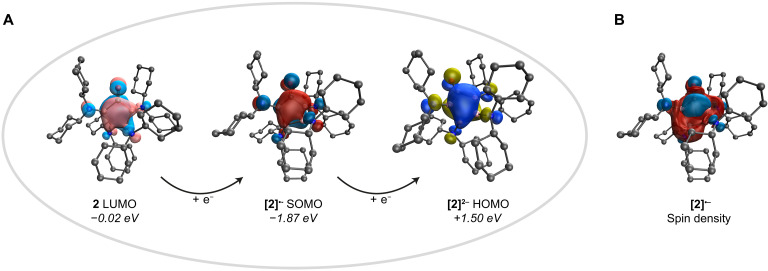
Electronic structures of 2 and its reduction products according to quantum chemical studies. (**A**) LUMO of **2**, and orbitals directly relevant to its one- and two-electron reduction ([**2**]^•−^ SOMO, [**2**]^2−^ HOMO; isosurface plots at 0.04 a.u.; calculated eigenvalues in italics). (**B**) Spin density plot of [**2**]^•−^ (isosurface plot at 0.004 a.u.) The SMD-ωB97xd/def2-TZVP//ωB97xd/def2SVPP level of theory was used for all DFT calculations.

**Fig. 7.**
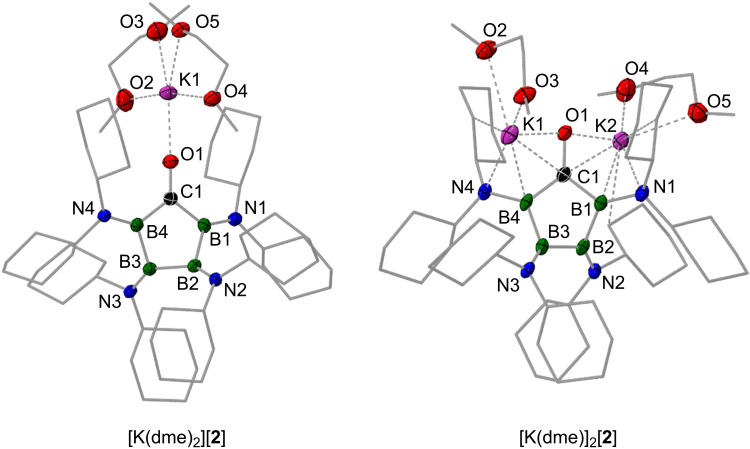
Structures of reduced derivatives of ketone 2 in the solid state. Molecular structures of [K(dme)_2_][**2**] (monoanion) and [K(dme)]_2_[**2**] (dianion). Displacement ellipsoids are shown at the 50% probability level, and hydrogen atoms and ellipsoids of peripheral carbon atoms are omitted for clarity.

We next focused on the redox behavior of ketone **2**, applying a combination of electrochemical techniques and experimental efforts. Cyclovoltammetry of **2** in tetrahydrofuran (thf) solution revealed an irreversible oxidation event at *E*_pa_ = +0.12 V and a partially reversible reduction process at *E*_1/2_ = −2.56 V, the latter presumably indicating the generation of the corresponding radical anion. No signs for the further reduction to the dianion were visible within the limits of the solvent window. The radical anion of **2** was accessible on a synthetic scale by reacting **2** with one equivalent of potassium graphite (KC_8_) in dimethoxyethane (dme) solution (Fig. 5). The initially dark red solution gradually turned to yellow over a period of 30 min, the successful formation of the radical anion [**2**]^•−^ being confirmed by its electron paramagnetic resonance (EPR) spectrum, which showed a broad, unresolved signal centered around a *g* value of 2.0047. Results of our DFT calculations suggest a delocalization of the unpaired electron across the entire B_4_C ring, even spanning the adjacent oxygen and nitrogen centers to some extent, as visualized by the spin density distribution of [**2**]^•−^ shown in Fig. 6B. Moreover, the singly occupied molecular orbital (SOMO) holding the extra electron provided by reduction shares roughly the same general shape, also indicating considerable delocalization effects, as does the molecular orbital (LUMO) of the precursor ketone **2** receiving this electron, which is to be expected (Fig. 6A). The radical anion K[**2**] was isolated as a dark yellow solid in 49% yield, single crystals of which were essential to validate its formation and, furthermore, to identify its composition in the solid state by x-ray diffraction as [K(dme)_2_][**2**] (Fig. 7; for a discussion of its molecular structure, see below).

Despite being undetected by cyclovoltammetry, two-electron reduction of **2** to its dianion [**2**]^2−^ was feasible by using two equivalents of KC_8_ or potassium metal (Fig. 5A). Carbonylation of our previously described dianion [K(dme)]_2_[**1**] with CO provided alternative access to K_2_[**2**] (Fig. 5B). In either case, we managed to isolate the salt as an orange solid in yields of up to 58%, which is defined by two distinct ^11^B NMR (δ = 46.4 and 39.1 ppm) and one carbonyl carbon ^13^C NMR resonance (δ = 190.0 ppm) in solution, all signals being shifted to lower frequencies with respect to **2** [δ(^11^B) = 61.1 and 47.8 ppm; δ(^13^C) = 305 ppm]. Consistent with our discussion of radical anion [**2**]^•−^, the accepting orbital of [**2**]^•−^ (SOMO) and the receiving orbital of dianion [**2**]^2−^ (HOMO) strongly resemble each other in shape (Fig. 6A). The x-ray diffraction structures of both reduced carbonyls are depicted in Fig. 7. As can be seen, the potassium ion of the radical anion [K(dme)_2_][**2**] is directly attached to the oxygen atom of the reduced ketone functionality [K⋯O distance, 2.439(1) Å] and is further stabilized by coordination of two chelating dme ligands, while the two potassium ions of dianion [K(dme)]_2_[**2**] are symmetrically surrounded by a ligand sphere involving the carbonyl oxygen atom, one dme ligand, and one boron-nitrogen bonds. A closer look on the heterocycle’s metric parameters reveals some intriguing structural changes during the two successive one-electron reduction processes, following a distinct trend as more electrons are put into the ring system (Table 1). Most noteworthy are the following observations: (i) The C–O bond is elongated from 1.240(2) Å in **2** to 1.308(2) Å and 1.374(3) Å in [**2**]^•−^ and [**2**]^2−^, respectively, as is the average B–N bond [**2**, 1.403(3) Å; [**2**]^•−^, 1.430(6) Å; [**2**]^2−^, 1.464(6) Å]. Note: For dianion [K(dme)]_2_[**2**], B–N bonds coordinated to the potassium ions [B1–N1, 1.474(3) Å; B4–N4, 1.489(2) Å] are substantially longer than their uncoordinated counterparts [B2–N2, 1.443(3) Å; B3–N3, 1.450(3) Å]. (ii) B–B [**2**, 1.722(3) Å; [**2**]^•−^, 1.710(5) Å; [**2**]^2−^, 1.686(5) Å] and B–C bonds [**2**, 1.637(3) Å; [**2**]^•−^, 1.586(4) Å; [**2**]^2−^, 1.550(4) Å] successively contract on average, when the electron density of the B_4_C heterocycle is increased. By contrast, the internal bond angle at carbon [**2**, 105.56(9)°; [**2**]^•−^, 106.9(1)°; [**2**]^2−^, 106.4 (2)°] is rather unaffected by the charge state of the B_4_C system.

**Table 1.** Comparison of key structural parameters of ketone 2 across different charge states.

	C–O (Å)	Avg. B–C (Å)	Avg. B–B (Å)	Avg. B–N (Å)	B–C–B (°)
**2**	1.240(2)	1.637(3)	1.722(3)	1.403(3)	105.56(9)
[**2**]^•−^	1.308(2)	1.586(4)	1.710(5)	1.430(6)	106.9(1)
[**2**]^2−^	1.374(3)	1.550(4)	1.686(5)	1.464(6)	106.4(2)

Unlike its intriguing reduction chemistry, oxidation chemistry of ketone **2** is much less tangible, and all efforts to generate a radical cation of **2** have thus far proven unsuccessful. Oxidation attempts using various ferrocenium salts, tetracyanoquinodimethane (TCNQ), or silver triflate (AgOTf) resulted in nonselective reactions, yielding no isolable products.

## DISCUSSION

In conclusion, we have shown that cyclic tetra(amino)tetraborane B_4_(NCy_2_)_4_ is highly susceptible to carbonylation with CO, ultimately producing two different intriguing molecules: a monocarbonyl and a dicarbonyl compound resulting from single and double CO insertion into the B_4_ ring, respectively. Both products are stable, although the 1,3-dicarbonyl species is susceptible to a thermally induced skeletal isomerization, generating a bicyclic tetraborylethylene derivative at elevated temperatures, a process dictated by thermodynamics, i.e., the formation of stronger boron-oxygen bonds coupled with the cleavage of relatively weak boron-boron bonds. The monocarbonyl displays a rich reduction chemistry and readily accepts two extra electrons in a stepwise manner, all while retaining stability in both the 1− and 2− charge states, as evidenced by the isolation of the corresponding mono- and dianions. The synthetic potential of these unique carbonyl compounds is now being evaluated in our labs.

## METHODS

### General procedures

All manipulations were performed either under an atmosphere of dry argon or in vacuo using standard Schlenk line or glovebox techniques (*47*). Deuterated solvents were dried over molecular sieves and degassed by three freeze-pump-thaw cycles prior to use. All other solvents were distilled and degassed from appropriate drying agents. Both deuterated and nondeuterated solvents were stored under argon over activated 4-Å molecular sieves. Liquid-phase NMR spectra were acquired on a Bruker Avance 400 (^1^H, 400.6 MHz), a Bruker Avance 500 (^1^H, 500.1 MHz; ^11^B, 160.5 MHz; ^13^C, 125.8 MHz), a Bruker Avance 600 (^1^H, 600.2 MHz; ^11^B, 192.6 MHz; ^13^C, 150.9 MHz) spectrometer. Chemical shifts (δ) are reported in ppm and internally referenced to the carbon nuclei (^13^C{^1^H}) or residual protons (^1^H) of the solvent. Heteronucleus NMR spectra are referenced to external standards (^11^B, BF_3_·OEt_2_). Resonances are given as singlet (s), multiplet (m), or broad (br). High-resolution mass spectrometry (HRMS) data were obtained from a Thermo Scientific Exactive Plus spectrometer. EPR measurements at X-band (9.86 GHz) were carried out using a Bruker ELEXSYS E580 CW EPR spectrometer. The spectral simulations were performed using MATLAB 9.12.0.1884302 (R2022a) and the EasySpin 5.2.33 toolbox (*48*). Cyclic voltammetry experiments were performed using a Gamry Instruments Reference 600 potentiostat. A standard three-electrode cell configuration was used using a platinum disk working electrode, a platinum wire counter electrode, and a silver wire, separated by a Vycor tip, serving as the reference electrode. Formal redox potentials are referenced to the ferrocene/ferrocenium ([Cp_2_Fe]^+/0^) redox couple by using decamethylferrocene ([Cp*_2_Fe], *E*_1/2_ = −0.427 V in THF) as an internal standard (*49*). Tetra(*n*-butyl)ammonium hexafluorophosphate ([*n*Bu_4_N][PF_6_]) was chosen as the supporting electrolyte. Compensation for resistive losses (iR drop) was used for all measurements. IR spectra in the solid state were recorded using an ALPHA II FTIR spectrometer with a diamond ATR sample head from Bruker in an argon atmosphere (glovebox). B_4_(NCy_2_)_4_ (**1**), [K(dme)]_2_[**1**], and KC_8_ were prepared on the basis of established literature procedures (*25*, *50*). Solvents, CO gas, and potassium metal were purchased from Sigma-Aldrich, ABCR, or Alfa Aesar.

### Single-crystal x-ray diffraction

The crystallographic data of **2**, [K(dme)_2_][**2**], [K(dme)]_2_[**2**], **3**, and **4** were collected on a XtaLAB Synergy Dualflex HyPix diffractometer with a hybrid pixel array detector and multilayer mirror monochromated Cu_Kα_ or Mo_Kα_ radiation. The structures were solved using the intrinsic phasing method (*51*), refined with the ShelXL program (*52*) and expanded using Fourier techniques. All nonhydrogen atoms were refined anisotropically. Hydrogen atoms were included in structure factor calculations. All hydrogen atoms were assigned to idealized geometric positions.

### Computational details

All computations were performed using the Gaussian16 (Revision C.01) package (*53*). All structures were fully optimized without symmetry constraints at the ωB97xd level of theory using def2-SVPP basis sets for all atoms (*54*, *55*). Zero-point vibrational energies and thermal corrections were computed from frequency calculations with a standard state of 298 K and 1 atm; thermal free energies (Δ*E*_298_) were obtained from these single-point frequency calculations. The presence of true energy minima on the potential energy surface was verified for all optimized species by the absence of imaginary frequencies. Predicted vibrational C=O frequencies were determined by applying a scaling factor of 0.91293, which was obtained by calculating the C=O stretching vibrations for a “training set” of 10 different molecules (four boron compounds/six standard organics) at this level of theory and comparison to their experimental values (see the Supplementary Materials for specifics of the training set). For EPR, TD-DFT, and orbital calculations, def2-TZVP basis sets were used, combined with the SMD solvation model (scrf = smd) for inclusion of tetrahydrofuran solvent effects (*56*). Illustrations of optimized structures as well as orbital and spin density plots were prepared with IQmol 3.1.3 (*57*).

### Synthesis of B_4_(NCy_2_)_4_CO(2)

To a solution of B_4_(NCy_2_)_4_ (**1**, 40.0 mg, 52.3 μmol) in benzene (1 ml) in a J. Young NMR tube, CO (1 bar) was added by means of three freeze-pump-thaw cycles, and the reaction mixture was stirred for 12 hours at room temperature, during which time a color change from blue to dark red was observed. Subsequently, all volatile components were removed in vacuo, yielding **2** as a dark red solid (35.2 mg, 44.4 μmol, 83%). By slowly evaporating a saturated solution of **2** in benzene at room temperature, suitable single crystals were obtained for x-ray structure analysis. ^1^H{^11^B} NMR (500.1 MHz, C_6_D_6_, 293.15 K): δ = 3.37 to 3.19 (m, 5H, CH), 2.98 to 2.90 (m, 1H, CH), 2.89 to 2.79 (m, 2H, CH), and 2.06 to 0.98 (m, 80H, CH_2_) ppm. ^13^C{^1^H,^11^B} NMR (150.9 MHz, C_6_D_6_, 293.15 K): δ = 305.0 (CO), 65.7 (CH), 64.3 (CH), 56.7 (CH), 36.4 (CH_2_), 36.2 (CH_2_), 35.9 (CH_2_), 35.7 (CH_2_), 35.1 (CH_2_), 34.6 (CH_2_), 33.8 (CH_2_), 33.3 (CH_2_), 33.0 (CH_2_), 27.6 (CH_2_), 27.5 (CH_2_), 27.3 (CH_2_), 27.1 (CH_2_), 27.0 (CH_2_), 26.9 (CH_2_), 26.9 (CH_2_), 26.8 (CH_2_), 26.7 (CH_2_), 26.7 (CH_2_), 26.5 (CH_2_), 26.3 (CH_2_), 26.2 (CH_2_), and 26.2 (CH_2_) ppm. ^11^B NMR (192.6 MHz, C_6_D_6_, 293.15 K): δ = 61.1 (br s) and 47.8 (br s) ppm. HRMS LIFDI (liquid injection field desorption ionization) for [C_49_H_88_B_4_N_4_O]^+^ = [M]^+^: calculated, 792.7325; found, 792.7305. FTIR (solid state): $\tilde{\nu }$(CO) = 1578 cm^−1^.

### Synthesis of K[2]

A solid mixture of **2** (30.0 mg, 37.9 μmol) and KC_8_ (5.12 mg, 37.9 μmol, 1.0 equiv) was combined with dme (4 ml). Subsequently, the reaction mixture was stirred for 30 min, whereupon its color changed from dark red to yellow. After separation from insoluble material by filtration, all volatiles were removed in vacuo, which yielded K[**2**] as a dark yellow solid (15.5 mg, 18.6 μmol, 49%). Single crystals of [K(dme)_2_][**2**] suitable for x-ray diffraction analysis were obtained by slow recrystallization from dme at −30°C. Note: K[**2**] decomposes at high dilution, precluding characterization by UV-vis or HRMS measurements. Elemental analyses were attempted but yielded inconsistent results due to the presence of solvent molecules or decomposition products. EPR (X-band, dme, and room temperature): *g*_iso_ = 2.0047.

### Synthesis of K_2_[2]

A mixture of **1** (40.0 mg, 52.3 μmol) and potassium (4.09 mg, 104.6 μmol, 2.0 equiv) in dme (2 ml) was stirred for 2 hours at room temperature, which was accompanied by a gradual color change to dark red-brown. Insoluble material was separated from the reaction mixture by filtration. CO (1 bar) was then introduced using four freeze-pump-thaw cycles, resulting in an immediate color change from dark red-brown to orange. Removal of all volatiles from the reaction mixture in vacuo yielded K_2_[**2**] as an orange solid (26.5 mg, 30.4 μmol, 58%). Single crystals of [K(dme)]_2_[**2**] suitable for x-ray diffraction analysis were obtained by slow recrystallization from dme at −30°C.

#### Alternative synthesis of K_2_[2]

A mixture of **2** (50.0 mg, 63.1 μmol) and potassium (4.93 mg, 12.6 μmol, 2.0 equiv) in dme (4 ml) was stirred at room temperature for 30 min, during which time the color of the reaction mixture changed from red to orange. Insoluble material was separated by filtration, and K_2_[**2**] was isolated as an orange solid (16.1 mg, 18.5 μmol, 29%) after removal of all volatiles from the reaction mixture in vacuo. Note: **2** can also be converted into its dianion K_2_[**2**] by reduction with KC_8_ with isolated yields of up to 26%. K_2_[**2**] readily decomposes at high dilution, precluding its characterization by UV-vis absorption spectroscopy. ^1^H{^11^B} NMR (500.1 MHz, d_8_-thf, 273.15 K): δ = 3.40 to 3.19 (m, 2H, CH), 3.08 to 2.77 (m, 6H, CH), and 2.11 to 0.69 (m, 80H, CH_2_ and 4H, CH_2_ of thf) ppm. ^13^C{^1^H} NMR (125.8 MHz, d_8_-thf, 263.15 K): δ = 190.0 (CO), 63.3 (CH), 56.5 (CH), 36.3 (CH_2_), 35.8 (CH_2_), 35.2 (CH_2_), 34.9 (CH_2_), 31.0 (CH_2_), 28.9 (CH_2_), 28.5 (CH_2_), 28.2 (CH_2_), 28.0 (CH_2_), 27.8 (CH_2_), 27.5 (CH_2_), and 27.2 (*C*H_2_) ppm. ^11^B NMR (160.5 MHz, d_8_-thf, 293.15 K): δ = 46.4 (overlapping, v br) and 39.1 (br s) ppm. HRMS LIFDI for [C_49_H_90_B_4_N_4_O]^+^ = [M + 2H]^+^: calculated, 794.7481; found, 794.7467.

### Attempts to generate monoradical cation [2]^•+^

**2** was reacted with a range of oxidizing agents (1 equiv) in various solvents like benzene, 1,2-difluorobenzene, and mixtures of solvents (benzene/1,2-difluorobenzene and benzene/dichloromethane) at room temperature or 60°C. However, all reactions using [Fc][PF_6_], [Fc][BF_4_], [NO][SbF_6_], [Ag][OTf], [Ag][(OC(CF_3_)_3_)], and TCNQ proceeded unselectively.

### Synthesis of B_4_(NCy_2_)_4_(CO)_2_(3)

A solution of **1** (150 mg, 104 μmol) in benzene (10 ml) in a flask with a resealable Teflon stopcock was subjected to an atmosphere of CO (5 bar) via three freeze-pump-thaw cycles. The mixture was stirred for 2 days at room temperature, during which time a gradual color change from blue to pink occurred. After exchange of the atmosphere to argon, the solution was slowly concentrated by evaporation of the solvent without applying vacuum. The pink residue was washed twice with thf (2 × 2 ml), filtered, and dried again by slow evaporation of the solvent while avoiding vacuum conditions, which yielded **3** as a pink solid (110 mg, 134 μmol, 68%). Slow evaporation of a saturated solution of **3** in dichloromethane at −30°C produced single crystals suitable for x-ray structure analysis.

#### Alternative synthesis of B_4_(NCy_2_)_4_(CO)_2_(3)

A solution of **2** (50.0 mg, 63.1 μmol) in benzene (2 ml) was subjected to an atmosphere of CO (1 bar) via three freeze-pump-thaw cycles. The mixture was then stirred for 4 days at room temperature, which resulted in a gradual color change from red to pink. After exchange of the atmosphere to argon, the solution was slowly concentrated by evaporation of the solvent without applying vacuum. The pink residue was washed twice with thf (2 × 2 ml), filtered, and dried again by slow evaporation of the solvent while avoiding vacuum conditions, which allowed for the isolation of **3** as a pink solid (46.5 mg, 56.8 μmol, 90%). Note: The isolated solid exhibits limited solubility in aromatic and aliphatic solvents such as benzene and hexane and is almost insoluble in polar solvents such as thf and acetonitrile but shows high solubility in dichloromethane and chloroform. **3** is highly sensitive to reduced pressure. ^1^H{^11^B} NMR (600.2 MHz, C_6_D_6_, 293.15 K): δ = 3.13 to 3.03 (m, 6H, CH), 2.98 to 2.88 (m, 2H, CH), and 2.16 to 0.98 (m, 80H, CH_2_) ppm. ^13^C{^1^H,^11^B} NMR (150.9 MHz, C_6_D_6_, 293.15 K): δ = 301.5 (CO), 65.8 (CH), 57.9 (CH), 36.9 (CH_2_), 36.3 (CH_2_), 34.9 (CH_2_), 34.3 (CH_2_), 33.8 (CH_2_), 27.3 (CH_2_), 27.1 (CH_2_), 26.9 (CH_2_), 26.7 (CH_2_), 26.5 (CH_2_), 26.3 (CH_2_), 26.1 (CH_2_), 25.9 (CH_2_), and 25.8 (CH_2_) ppm. ^11^B NMR (160.5 MHz, C_6_D_6_, 293.15 K): δ = 60.6 (v br shoulder), 55.6 (br), and 33.1 (br) ppm. HRMS LIFDI for [C_50_H_88_B_4_N_4_O_2_]^+^ = [M]^+^: calculated, 820.7273; found, 820.7274. FTIR (solid state): $\tilde{\nu }$(CO) = 1549 and 1522 cm^−1^.

### Synthesis of 4

A solution of **3** (30.0 mg, 36.6 μmol) in benzene (1 ml) was heated at 80°C over a period of 1 week, whereupon the color of the reaction mixture gradually changed from pink to yellow. After removal of all volatiles in vacuo, the residue was washed with hexamethyldisiloxane (6 × 0.2 ml), filtered, and dried under reduced pressure. Through this, **4** was isolated as a colorless solid (17.0 mg, 20.7 μmol, 57%). Slow evaporation of a saturated solution of **4** in benzene at room temperature produced single crystals suitable for x-ray structure analysis. ^1^H{^11^B} NMR (400.6 MHz, C_6_D_6_, 293.15 K): δ = 3.78 to 3.36 (m, 2H, CH), 3.08 to 2.31 (m, 6H, CH), and 2.09 to 0.77 (m, 80H, CH_2_) ppm. ^13^C{^1^H,^11^B} NMR (150.9 MHz, C_6_D_6_, 333.15 K): δ = 190.9 (C_q_), 56.5 (CH), 35.1 (CH_2_), 34.7 (CH_2_), 34.0 (CH_2_), 27.2 (CH_2_), 26.8 (CH_2_), 26.3 (CH_2_), and 25.5 (CH_2_). ^11^B NMR (160.5 MHz, C_6_D_6_, 293.15 K): δ = 34.2 (br) ppm. HRMS LIFDI for [C_50_H_88_B_4_N_4_O_2_]^+^ = [M]^+^: calculated, 820.7274; found, 820.7267.

## Supplementary Materials

### This PDF file includes:

Supplementary Text Figs. S1 to S28 Tables S1 and S2 Cartesian Coordinates of DFT Optimized Structures References
