# Supplementary material for: Capturing carbon monoxide—Accessing bis(boryl)ketones through direct B–B bond carbonylation
Source: Sci Adv. 2025 Aug 27;11(35):eadz3520. doi: 10.1126/sciadv.adz3520 (PMC12383245; doi:10.1126/sciadv.adz3520)
Supplement: Supplementary file 1 — Supplementary Text Figs. S1 to S28 Tables S1 and S2 Cartesian Coordinates of DFT Optimized Structures References [file sciadv.adz3520_sm.pdf]

Science Advances
AAAS

Supplementary Materials for

Capturing carbon monoxide—Accessing bis(boryl)ketones through direct B–B bond carbonylation

Eva Beck *et al.*

Corresponding author: Holger Braunschweig, h.braunschweig@uni-wuerzburg.de

Sci. Adv. **11**, eadz3520 (2025)
DOI: 10.1126/sciadv.adz3520

This PDF file includes:

Supplementary Text
Figs. S1 to S28
Tables S1 and S2
Cartesian Coordinates of DFT Optimized Structures
References

Methods

General (*47*): All manipulations were performed either under an atmosphere of dry argon or *in vacuo* using standard Schlenk line or glovebox techniques. Deuterated solvents were dried over molecular sieves and degassed by three freeze-pump-thaw cycles prior to use. All other solvents were distilled and degassed from appropriate drying agents. Both deuterated and non-deuterated solvents were stored under argon over activated 4 Å molecular sieves. Liquid-phase NMR spectra were acquired on a Bruker Avance 400 (^{1}H: 400.6 MHz), a Bruker Avance 500 (^{1}H: 500.1 MHz, ^{11}B: 160.5 MHz, ^{13}C: 125.8 MHz), a Bruker Avance 600 (^{1}H: 600.2 MHz, ^{11}B: 192.6 MHz, ^{13}C: 150.9 MHz) spectrometer. Chemical shifts (δ) are reported in ppm and internally referenced to the carbon nuclei (^{13}C{^{1}H}) or residual protons (^{1}H) of the solvent. Heteronucleus NMR spectra are referenced to external standards (^{11}B: $BF_3{\cdot}OEt_2$). Resonances are given as singlet (s), multiplet (m) or broad (br). High-resolution mass spectrometry (HRMS) data were obtained from a *Thermo Scientific Exactive Plus* spectrometer. EPR measurements at X-band (9.86 GHz) were carried out using a *Bruker ELEXSYS E580 CW* EPR spectrometer. The spectral simulations were performed using *MATLAB 9.12.0.1884302 (R2022a)* and the *EasySpin 5.2.33 toolbox* (*48*). Cyclic voltammetry experiments were performed using a *Gamry Instruments Reference 600* potentiostat. A standard three-electrode cell configuration was employed using a platinum disk working electrode, a platinum wire counter electrode, and a silver wire, separated by a Vycor tip, serving as the reference electrode. Formal redox potentials are referenced to the ferrocene/ferrocenium ($[Cp_2Fe]^{+/0}$) redox couple by using decamethylferrocene ($[Cp^*{}_2Fe]$, $E_{1/2}$ = −0.427 V in THF) as an internal standard (*49*). Tetra(*n*-butyl)ammonium hexafluorophosphate ($[n\text{Bu}_4\text{N}][\text{PF}_6]$) was chosen as the supporting electrolyte. Compensation for resistive losses (iR drop) was employed for all measurements. IR spectra in the solid state were recorded using an *ALPHA II* FT-IR spectrometer with a diamond ATR sample head from *Bruker* in an argon atmosphere (glovebox). $B_4(NCy_2)_4$ (**1**), $[K(dme)]_2$[**1**], and KC_8 were prepared based on established literature procedures (*25,50*). Solvents, CO gas, and potassium metal were purchased from Sigma-Aldrich, ABCR, or Alfa Aesar.

Experimental Procedures

Synthesis of $B_4(NCy_2)_4CO$ (**2**)

To a solution of $B_4(NCy_2)_4$ (**1**, 40.0 mg, 52.3 μmol) in benzene (1 mL) in a J. Young NMR tube was added CO (1 bar) by means of three *freeze-pump-thaw cycles*, and the reaction mixture was stirred for 12 h at room temperature, during which time a color change from blue to dark red was observed. Subsequently, all volatile components were removed *in vacuo*, yielding **2** as a dark red solid (35.2 mg, 44.4 μmol, 83%). By slowly evaporating a saturated solution of **2** in benzene at room temperature, suitable single crystals were obtained for X-ray structure analysis.

^{1}H{^{11}B} NMR (500.1 MHz, C_6D_6, 293.15 K): δ = 3.37–3.19 (m, 5H, C*H*), 2.98–2.90 (m, 1H, C*H*), 2.89–2.79 (m, 2H, C*H*), 2.06–0.98 (m, 80H, CH_2) ppm. **^{13}C{^{1}H,^{11}B} NMR** (150.9 MHz, C_6D_6, 293.15 K): δ = 305.0 (*C*O), 65.7 (*C*H), 64.3 (*C*H), 56.7 (*C*H), 36.4 (*C*H_2), 36.2 (*C*H_2), 35.9 (*C*H_2), 35.7 (*C*H_2), 35.1 (*C*H_2), 34.6 (*C*H_2), 33.8 (*C*H_2), 33.3 (*C*H_2), 33.0 (*C*H_2), 27.6 (*C*H_2), 27.5 (*C*H_2), 27.3 (*C*H_2), 27.1 (*C*H_2), 27.0 (*C*H_2), 26.9 (*C*H_2), 26.9 (*C*H_2), 26.8 (*C*H_2), 26.7 (*C*H_2), 26.7 (*C*H_2), 26.5 (*C*H_2), 26.3 (*C*H_2), 26.2 (*C*H_2), 26.2 (*C*H_2) ppm. **^{11}B NMR** (192.6 MHz, C_6D_6, 293.15 K): δ = 61.1 (br s), 47.8 (br s) ppm. **HRMS LIFDI** for $[C_{49}H_{88}B_4N_4O]^+$ = $[M]^+$: calcd. 792.7325, found 792.7305. **FT-IR** (solid-state): $\tilde{\nu}$(CO) = 1578 cm^{-1}.

Synthesis of K[**2**]

A solid mixture of **2** (30.0 mg, 37.9 μmol) and KC_8 (5.12 mg, 37.9 μmol, 1.0 eq.) was combined with dme (4 mL). Subsequently, the reaction mixture was stirred for 30 min, whereupon its color changed from dark red to yellow. After separation from insoluble material by filtration, all volatiles were removed *in vacuo*, which yielded K[**2**] as a dark yellow solid (15.5 mg, 18.6 μmol, 49%). Single crystals of $[K(dme)_2]$[**2**] suitable for X-ray diffraction analysis were obtained by slow recrystallization from dme at –30 °C.

*Note: K[**2**] decomposes at high dilution, precluding characterization by UV-Vis or HRMS measurements. Elemental analyses were attempted, but yielded inconsistent results due to the presence of solvent molecules or decomposition products.*

EPR (X-band, dme, room temperature): g_{iso} = 2.0047.

Synthesis of K_2[**2**]

A mixture of **1** (40.0 mg, 52.3 μmol) and potassium (4.09 mg, 104.6 μmol, 2.0 eq.) in dme (2 mL) was stirred for 2 h at room temperature, which was accompanied by a gradual color change to dark red-brown. Insoluble material was separated from the reaction mixture by filtration. CO (1 bar) was then introduced using four *freeze-pump-thaw cycles*, resulting in an immediate color change from dark red-brown to orange. Removal of all volatiles from the reaction mixture *in vacuo* yielded K_2[**2**] as an orange solid (26.5 mg, 30.4 μmol, 58%). Single crystals of $[K(dme)]_2$[**2**] suitable for X-ray diffraction analysis were obtained by slow recrystallization from dme at –30 °C. *Alternative synthesis of K_2[2].* A mixture of **2** (50.0 mg, 63.1 μmol) and

potassium (4.93 mg, 12.6 μmol, 2.0 eq.) in dme (4 mL) was stirred at room temperature for 30 min, during which time the color of the reaction mixture changed from red to orange. Insoluble material was separated by filtration, and K_2[**2**] was isolated as an orange solid (16.1 mg, 18.5 μmol, 29%) after removal of all volatiles from the reaction mixture *in vacuo*.

*Note: **2** can also be converted into its dianion K_2[**2**] by reduction with KC_8 with isolated yields of up to 26%. K_2[**2**] readily decomposes at high dilution, precluding its characterization by UV-vis absorption spectroscopy.*

^{1}H{^{11}B} NMR (500.1 MHz, d_8-thf, 273.15 K): δ = 3.40–3.19 (m, 2H, C*H*), 3.08–2.77 (m, 6H, C*H*), 2.11–0.69 (m, 80H, C*H*$_2$ *and* 4H, C*H*$_2$ of thf) ppm. **^{13}C{^{1}H} NMR** (125.8 MHz, d_8-thf, 263.15 K): δ = 190.0 (*C*O), 63.3 (*C*H), 56.5 (*C*H), 36.3 (*C*H$_2$), 35.8 (*C*H$_2$) 35.2 (*C*H$_2$), 34.9 (*C*H$_2$), 31.0 (*C*H$_2$), 28.9 (*C*H$_2$), 28.5 (*C*H$_2$), 28.2 (*C*H$_2$), 28.0 (*C*H$_2$), 27.8 (*C*H$_2$), 27.5 (*C*H$_2$), 27.2 (*C*H$_2$) ppm. **^{11}B NMR** (160.5 MHz, d_8-thf, 293.15 K): δ = 46.4 (overlapping, v br), 39.1 (br s) ppm. **HRMS LIFDI** for $[C_{49}H_{90}B_4N_4O]^+$ = $[M+2H]^+$: calcd. 794.7481, found 794.7467.

Attempts to generate monoradical cation [**2**]$^{•+}$

2 was reacted with a range of oxidizing agents (1 eq.) in various solvents like benzene, 1,2-difluorobenzene, and mixtures of solvents (benzene/1,2-difluorobenzene, and benzene/ dichloromethane) at room temperature or 60 °C. However, all reactions using [Fc][PF_6], [Fc][BF_4], [NO][SbF_6], [Ag][OTf], [Ag][($OC(CF_3)_3$)], and TCNQ proceeded unselectively.

Synthesis of $B_4(NCy_2)_4(CO)_2$ (**3**)

A solution of **1** (150 mg, 104 μmol) in benzene (10 mL) in a flask with a resealable Teflon stopcock was subjected to an atmosphere of CO (5 bar) *via* three *freeze-pump-thaw cycles*. The mixture was stirred for 2 d at room temperature, during which time a gradual color change from blue to pink occurred. After exchange of the atmosphere to argon, the solution was slowly concentrated by evaporation of the solvent without applying vacuum. The pink residue was washed twice with THF (2 × 2 mL), filtered and dried again by slow evaporation of the solvent, while avoiding vacuum conditions, which yielded **3** as a pink solid (110 mg, 134 μmol, 68%). Slow evaporation of a saturated solution of **3** in dichloromethane at –30 °C produced single crystals suitable for X-ray structure analysis. *Alternative synthesis of $B_4(NCy_2)_4(CO)_2$ (3).* A solution of **2** (50.0 mg, 63.1 μmol) in benzene (2 mL) was subjected to an atmosphere of CO (1 bar) *via* three *freeze-pump-thaw cycles*. The mixture was then stirred for 4 d at room temperature, which resulted in a gradual color change from red to pink. After exchange of the atmosphere to argon, the solution was slowly concentrated by evaporation of the solvent without applying vacuum. The pink residue was washed twice with THF (2 × 2 mL), filtered and dried again by slow evaporation of the solvent, while avoiding vacuum conditions, which allowed for the isolation of **3** as a pink solid (46.5 mg, 56.8 μmol, 90%).

Note: The isolated solid exhibits limited solubility in aromatic and aliphatic solvents such as benzene and hexane, is almost insoluble in polar solvents such as THF and acetonitrile, but

*shows high solubility in dichloromethane and chloroform. **3** is highly sensitive to reduced pressure.*

$^{1}H\{^{11}B\}$ NMR (600.2 MHz, C_6D_6, 293.15 K): δ = 3.13–3.03 (m, 6H, C*H*), 2.98–2.88 (m, 2H, C*H*), 2.16–0.98 (m, 80H, C*H*$_2$) ppm. **$^{13}C\{^{1}H,^{11}B\}$ NMR** (150.9 MHz, C_6D_6, 293.15 K): δ = 301.5 (*C*O), 65.8 (*C*H), 57.9 (*C*H), 36.9 (*C*H$_2$), 36.3 (*C*H$_2$) 34.9 (*C*H$_2$), 34.3 (*C*H$_2$), 33.8 (*C*H$_2$), 27.3 (*C*H$_2$), 27.1 (*C*H$_2$), 26.9 (*C*H$_2$), 26.7 (*C*H$_2$), 26.5 (*C*H$_2$), 26.3 (*C*H$_2$), 26.1 (*C*H$_2$), 25.9 (*C*H$_2$), 25.8 (*C*H$_2$) ppm. **^{11}B NMR** (160.5 MHz, C_6D_6, 293.15 K): δ = 60.6 (v br shoulder), 55.6 (br), 33.1 (br) ppm. **HRMS LIFDI** for $[C_{50}H_{88}B_4N_4O_2]^+ = [M]^+$: calcd. 820.7273, found 820.7274.
FT-IR (solid-state): $\tilde{\nu}$(CO) = 1549, 1522 cm^{-1}.

Synthesis of **4**

A solution of **3** (30.0 mg, 36.6 µmol) in benzene (1 mL) was heated at 80 °C over a period of one week, whereupon the color of the reaction mixture gradually changed from pink to yellow. After removal of all volatiles *in vacuo*, the residue was washed with hexamethyldisiloxane (6 × 0.2 mL), filtered and dried under reduced pressure. Through this, **4** was isolated as a colorless solid (17.0 mg, 20.7 µmol, 57%). Slow evaporation of a saturated solution of **4** in benzene at room temperature produced single crystals suitable for X-ray structure analysis.

$^{1}H\{^{11}B\}$ NMR (400.6 MHz, C_6D_6, 293.15 K): δ = 3.78–3.36 (m, 2H, C*H*), 3.08–2.31 (m, 6H, C*H*), 2.09–0.77 (m, 80H, C*H*$_2$) ppm. **$^{13}C\{^{1}H,^{11}B\}$ NMR** (150.9 MHz, C_6D_6, 333.15 K): δ = 190.9 (*C*$_q$), 56.5 (*C*H), 35.1 (*C*H$_2$), 34.7(*C*H$_2$), 34.0 (*C*H$_2$), 27.2 (*C*H$_2$), 26.8 (*C*H$_2$), 26.3 (*C*H$_2$), 25.5 (*C*H$_2$). **^{11}B NMR** (160.5 MHz, C_6D_6, 293.15 K): δ = 34.2 (br) ppm. **HRMS LIFDI** for $[C_{50}H_{88}B_4N_4O_2]^+ = [M]^+$: calcd. 820.7274, found 820.7267.

NMR Spectra

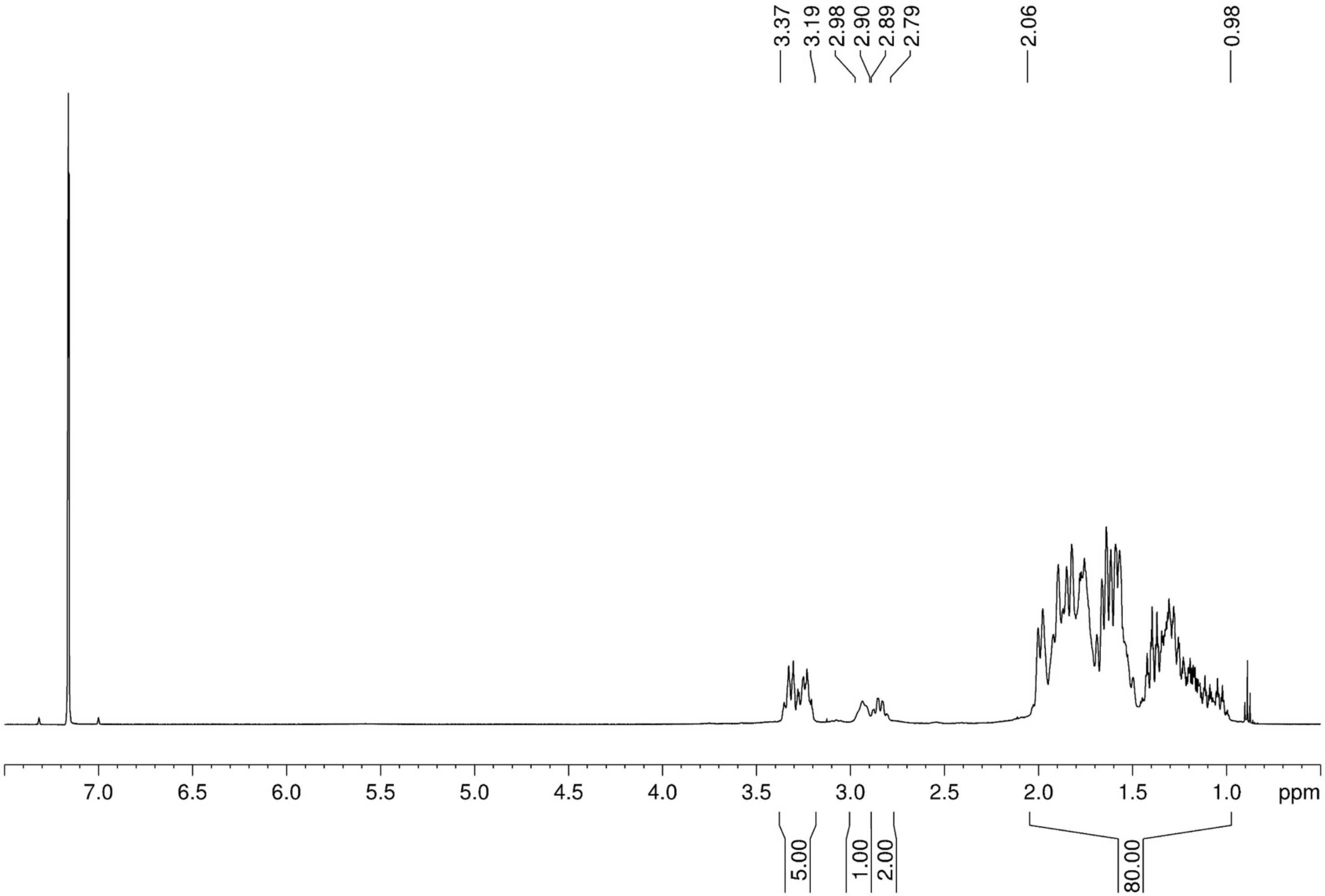

Fig. S1. $^{1}H\{^{11}B\}$ NMR spectrum of **2** in C_6D_6.

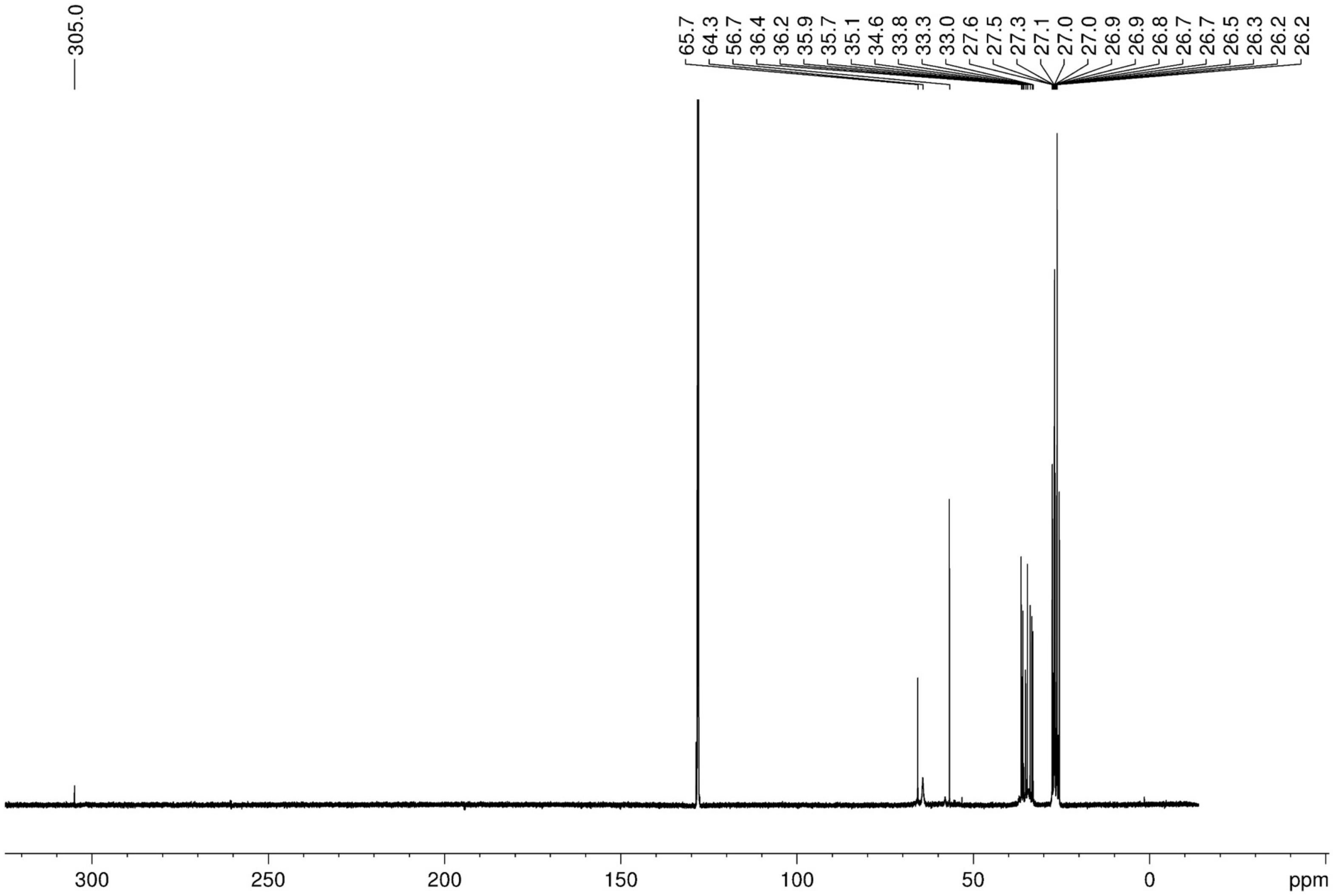

Fig. S2. $^{13}C\{^{1}H,^{11}B\}$ NMR spectrum of **2** in C_6D_6.

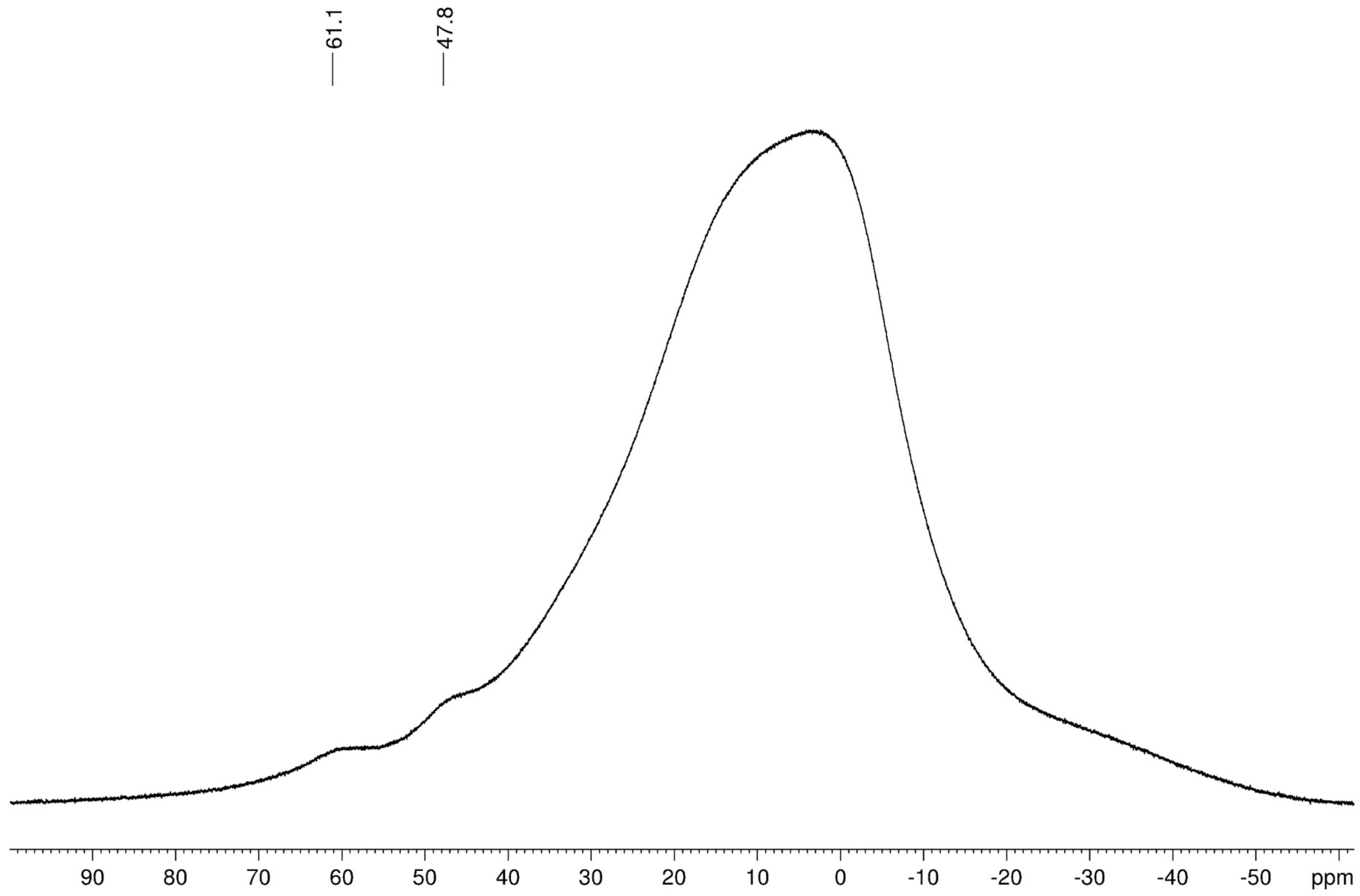

Fig. S3. ^{11}B NMR spectrum of **2** in C_6D_6.

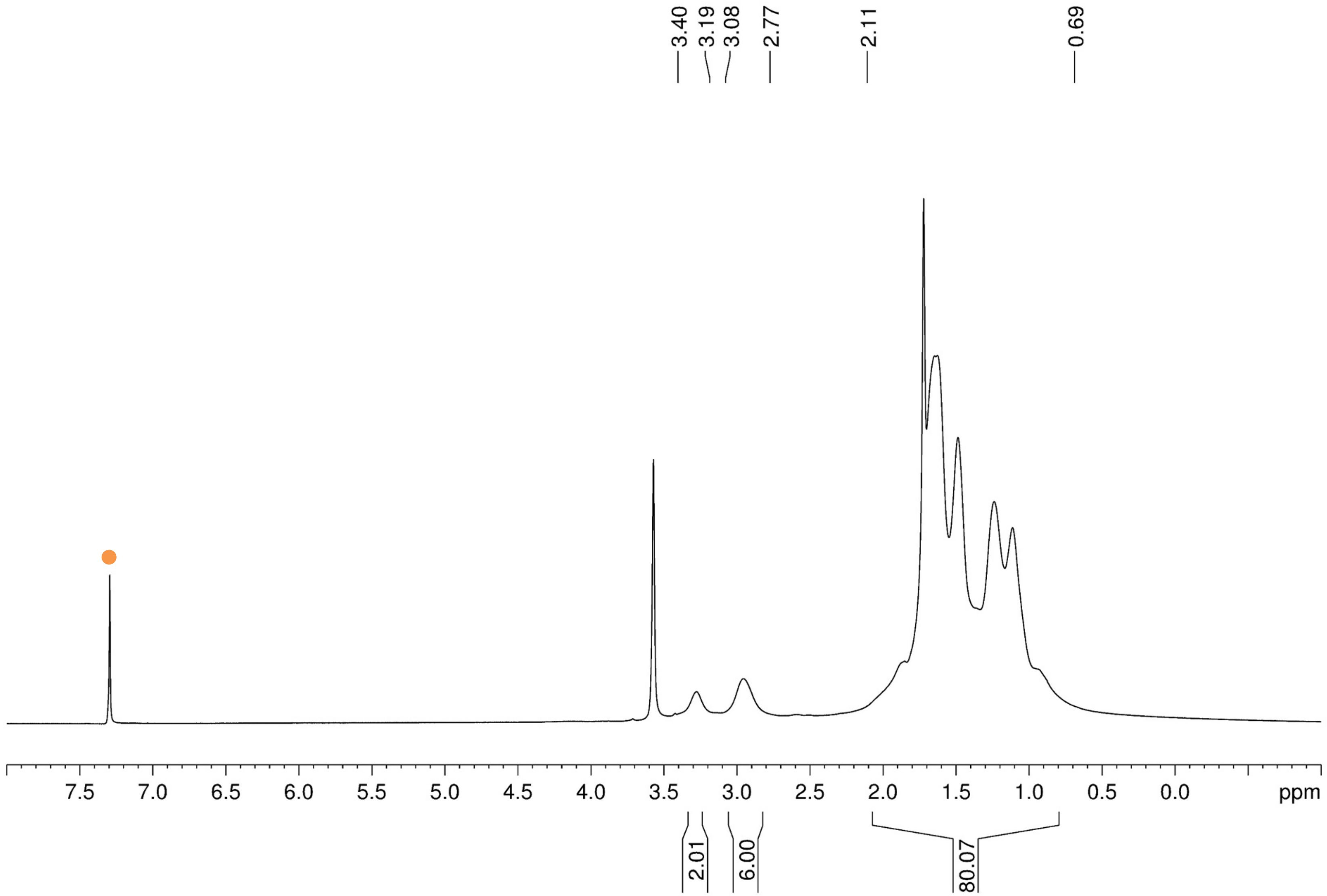

Fig. S4. $^{1}H\{^{11}B\}$ NMR spectrum of K_2[**2**] in d_8-thf (green dot = C_6H_6 impurities in d_8-thf).

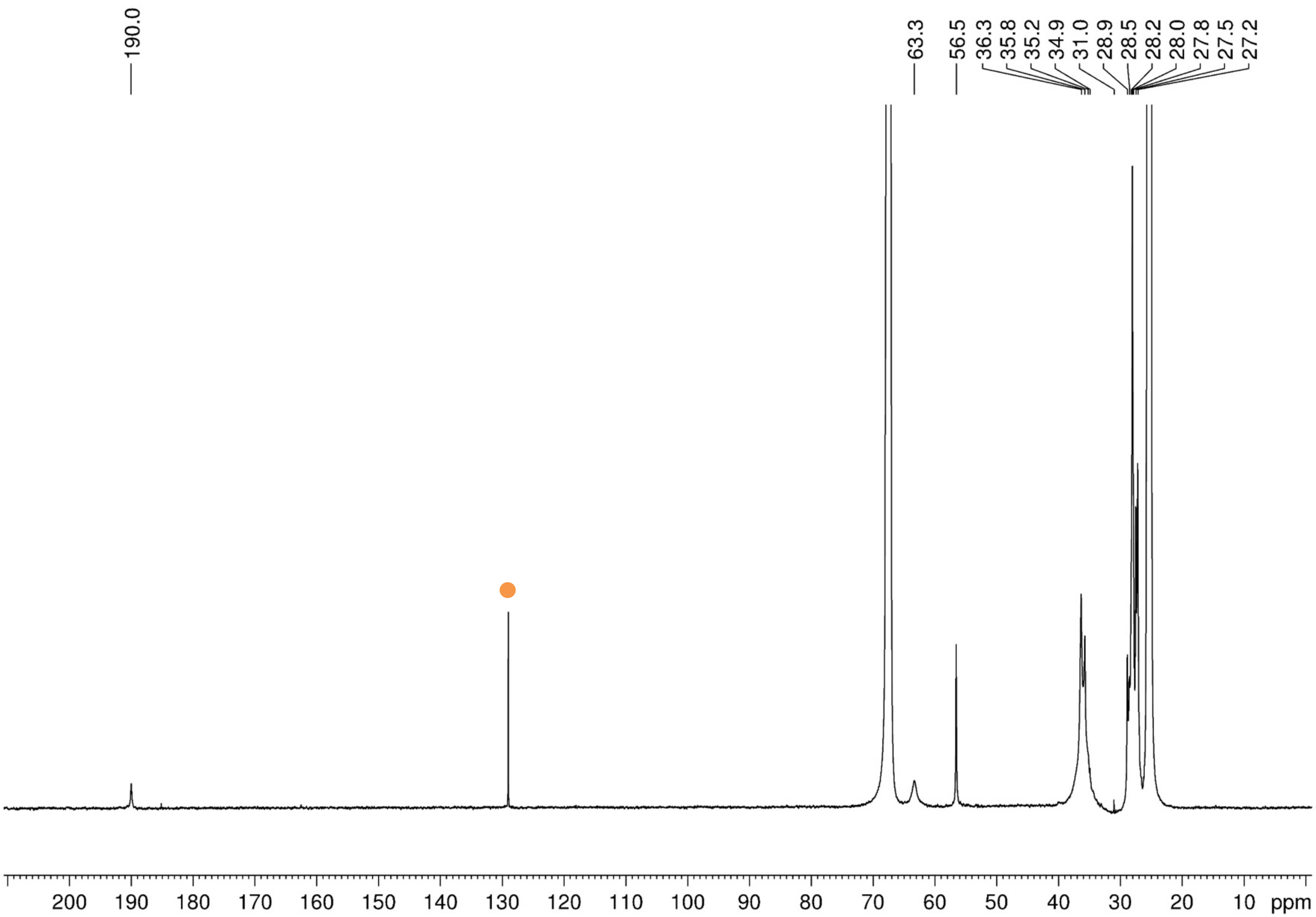

Fig. S5. $^{13}C\{^{1}H\}$ NMR spectrum K_2[**2**] in d_8-thf (green dot = C_6H_6 impurities in d_8-thf).

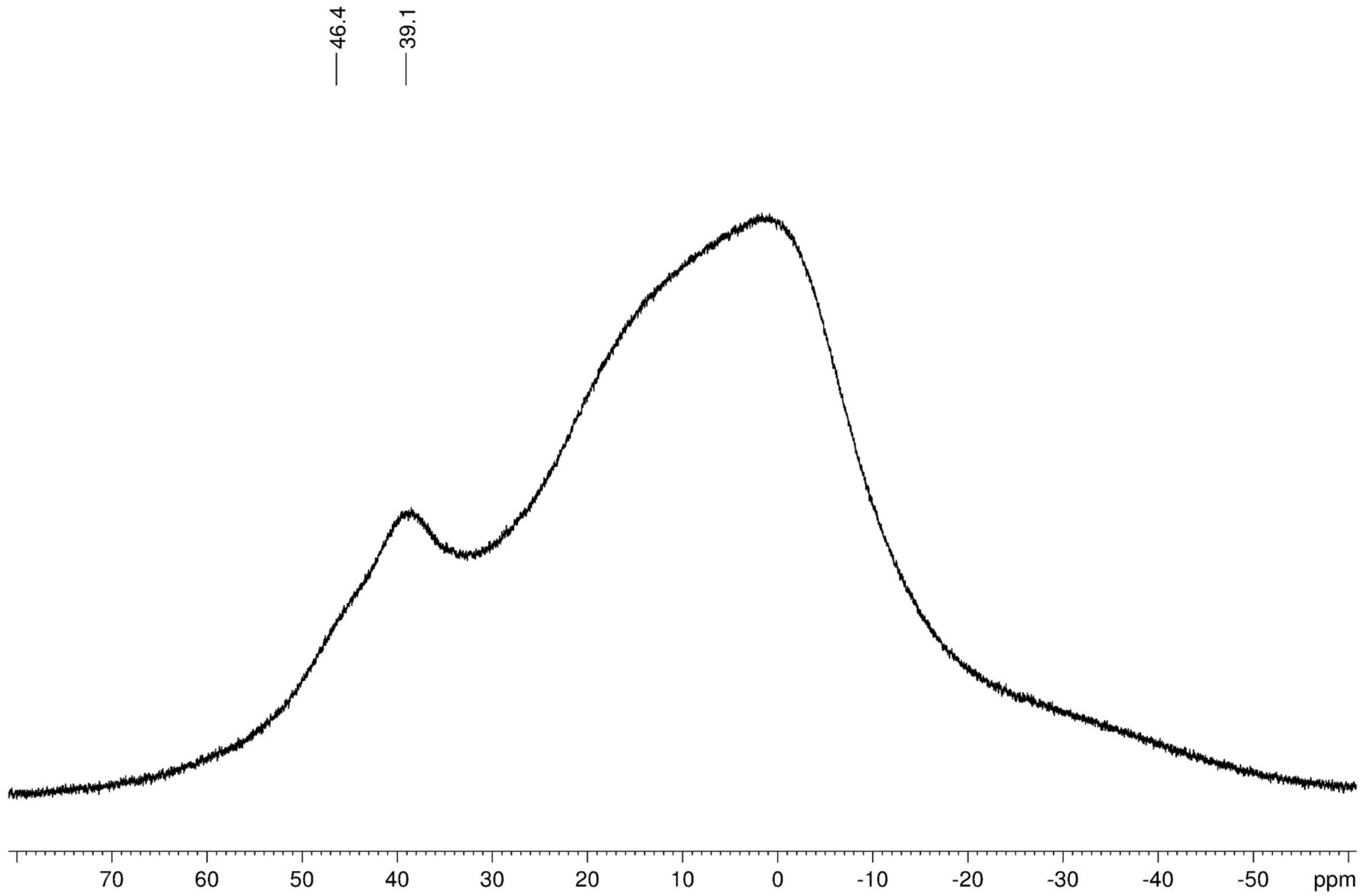

Fig. S6. ^{11}B NMR spectrum K_2[**2**] in d_8-thf (293 K).

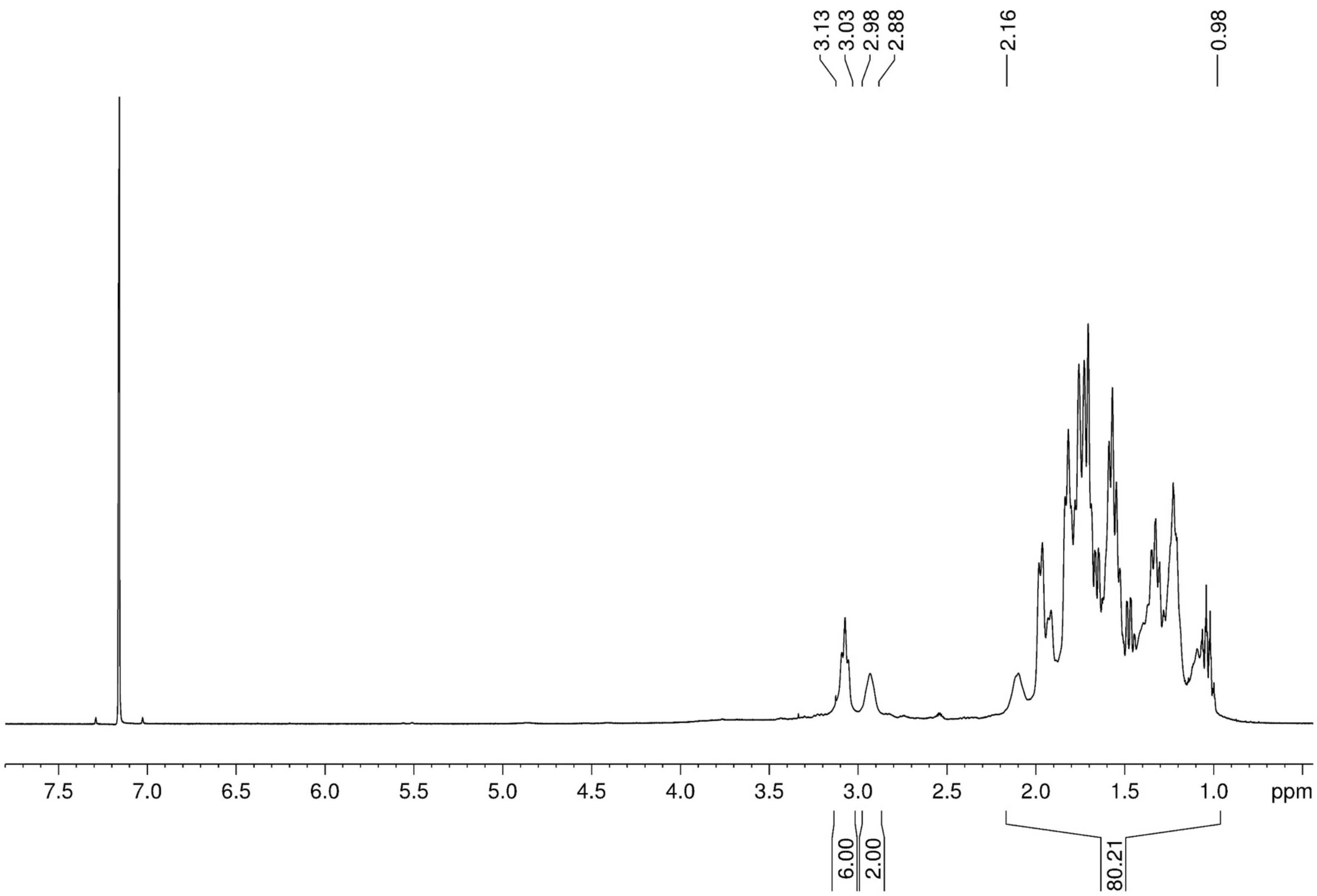

Fig. S7. $^{1}H\{^{11}B\}$ NMR spectrum of **3** in C_6D_6.

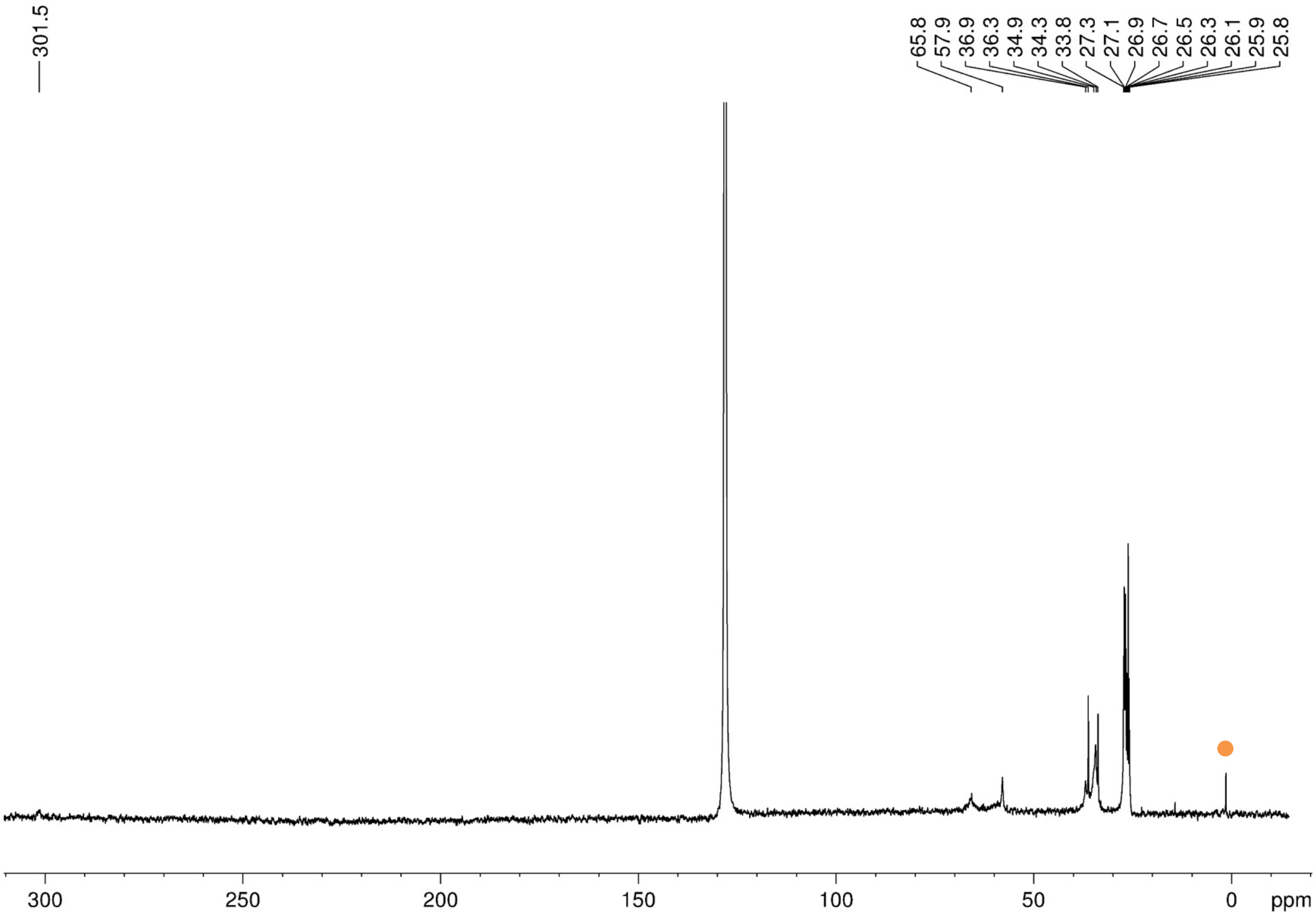

Fig. S8. $^{13}C\{^{1}H,^{11}B\}$ NMR spectrum of **3** in C_6D_6 (green dot = silicon grease).

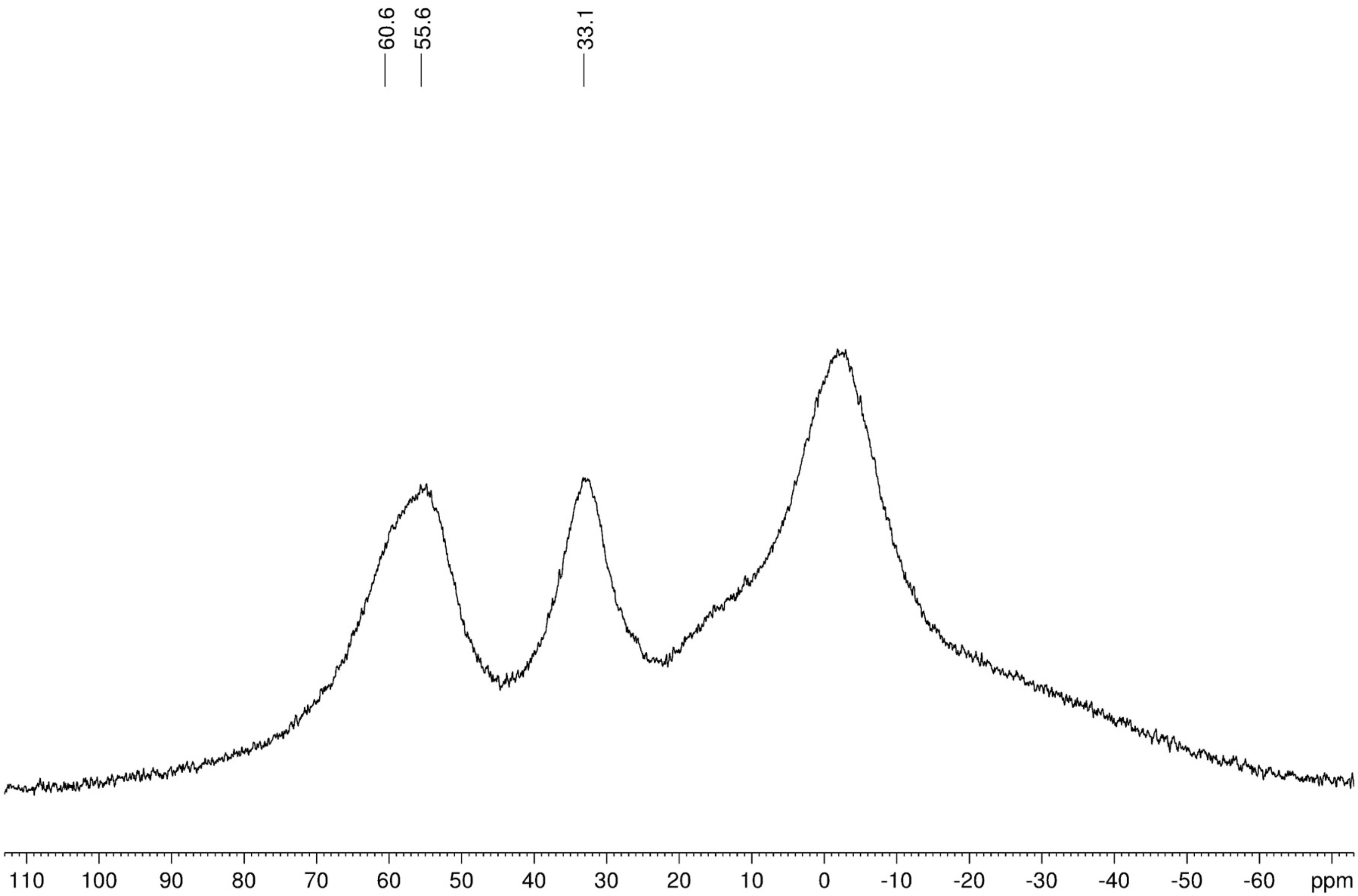

Fig. S9. ^{11}B NMR spectrum of **3** in C_6D_6.

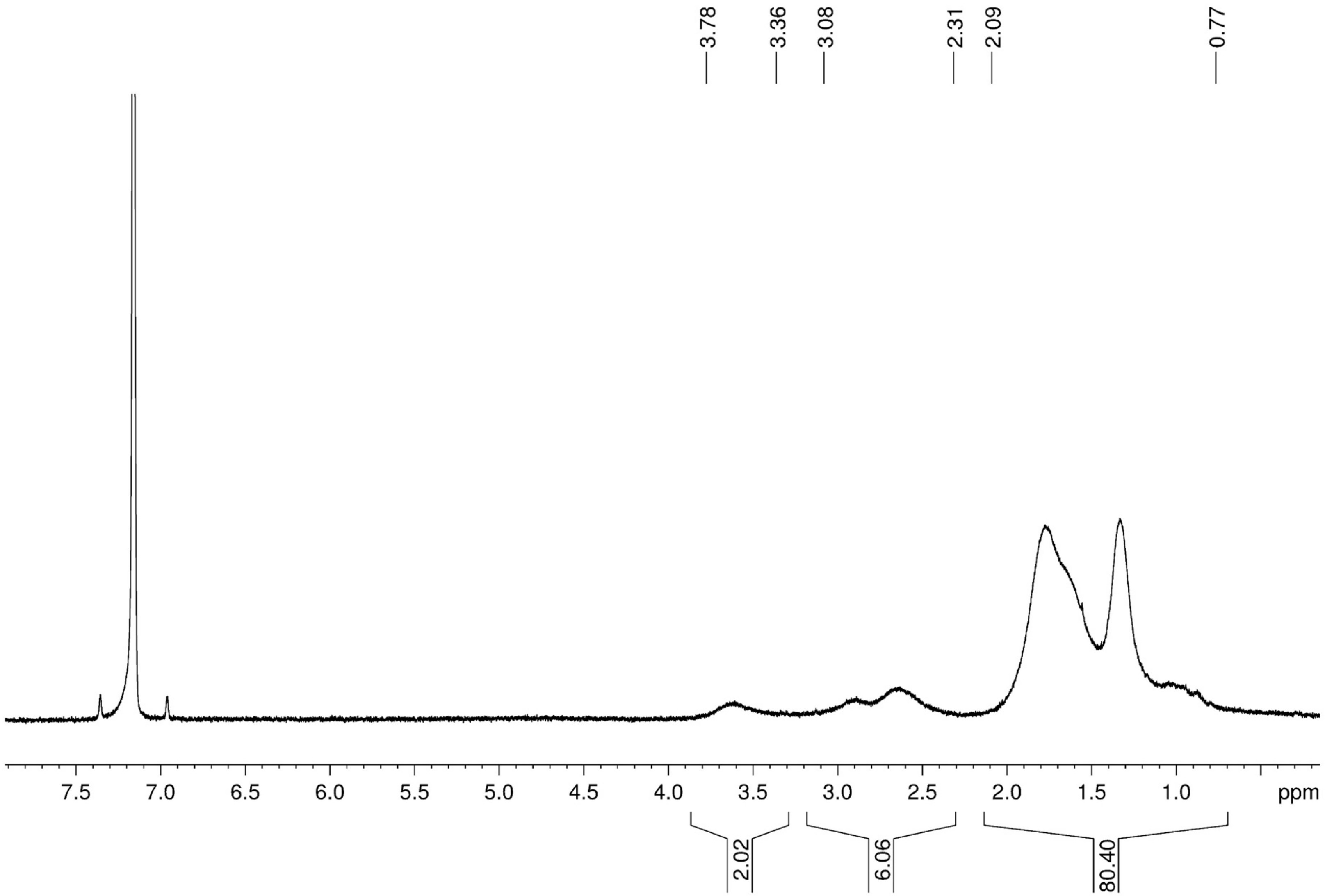

Fig. S10. ^{1}H NMR spectrum of **4** in C_6D_6.

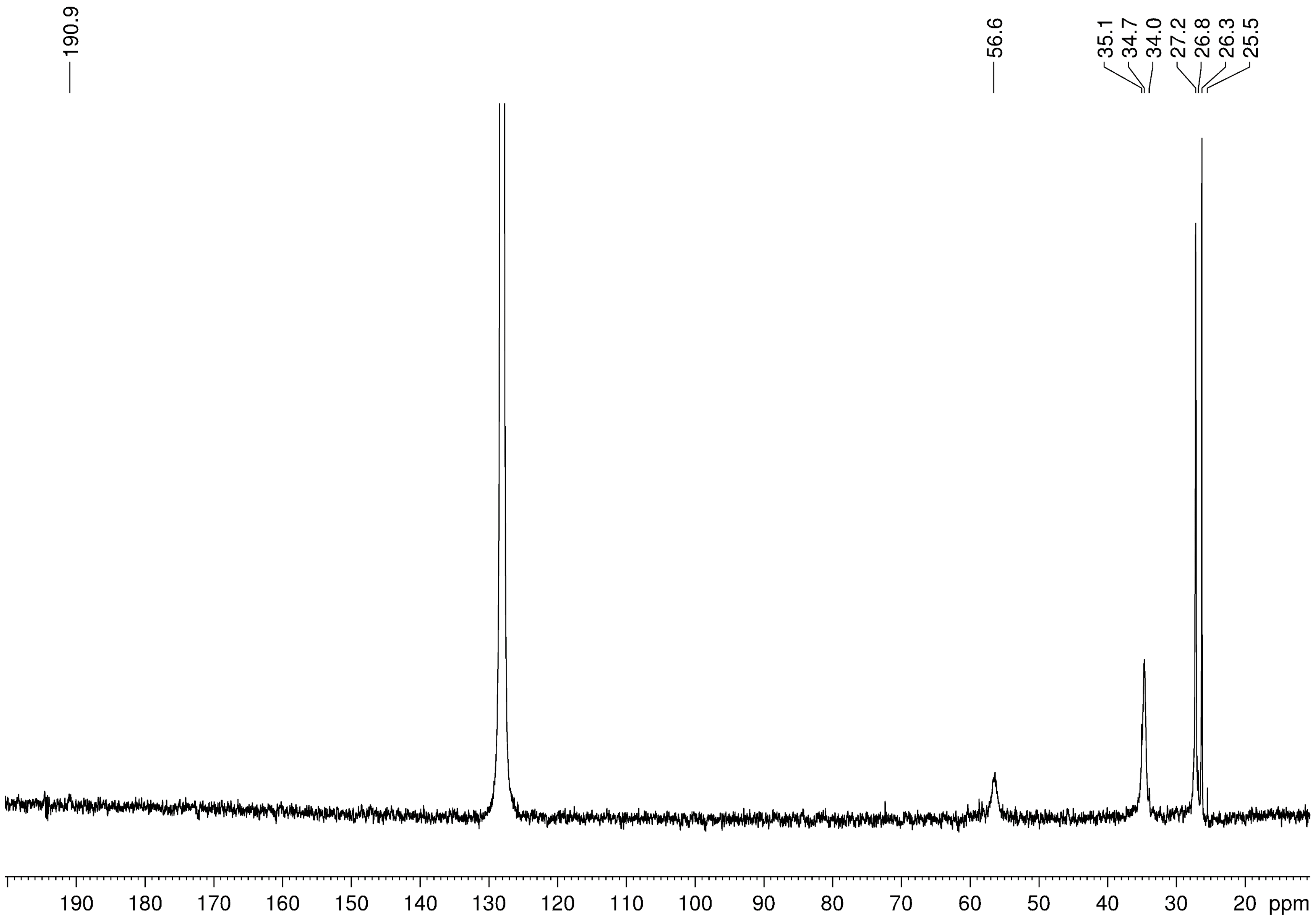

Fig. S11. ^{13}C NMR spectrum of **4** in C_6D_6.

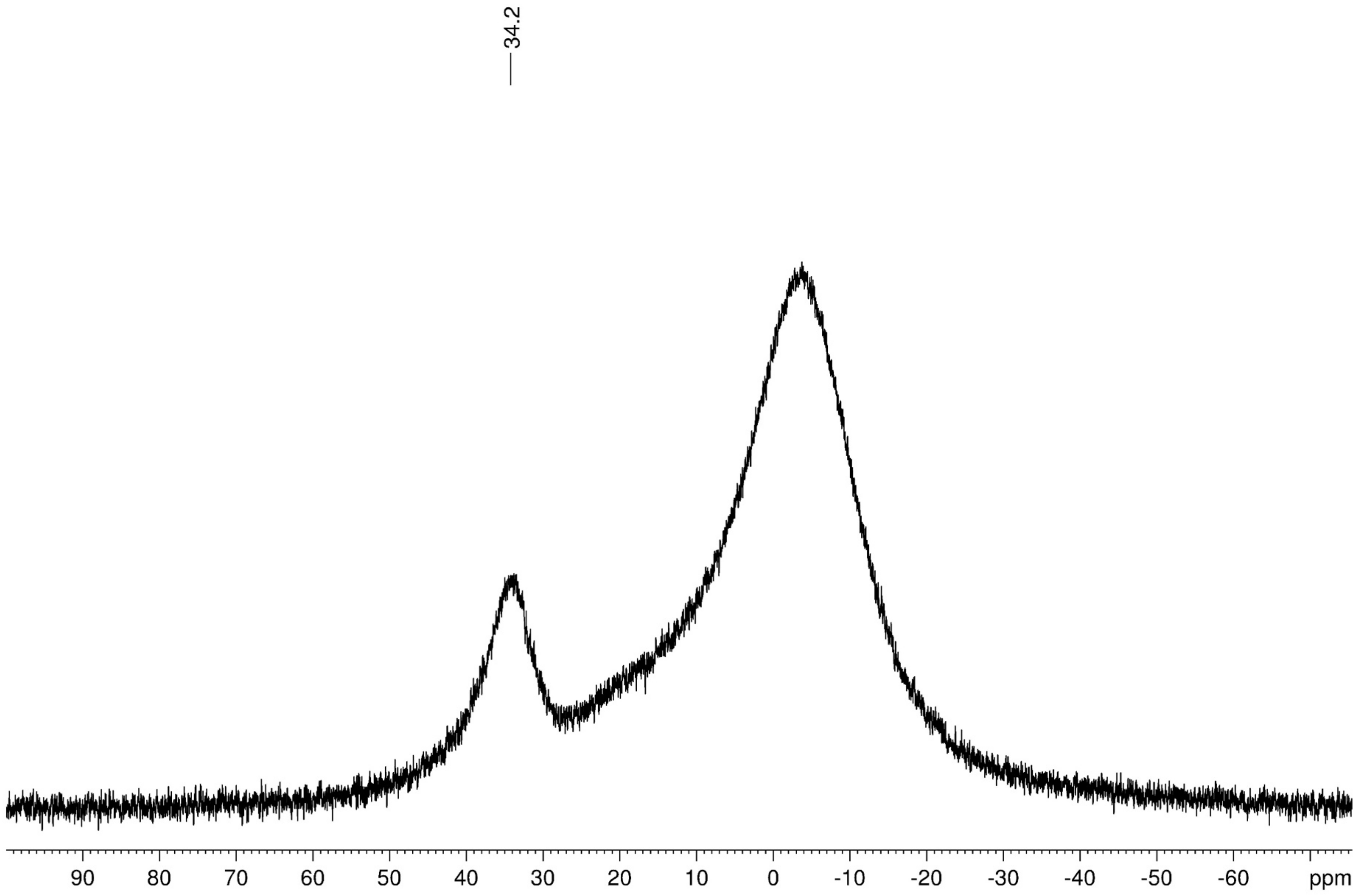

Fig. S12. ^{11}B NMR spectrum of **4** in C_6D_6.

UV-vis Spectra

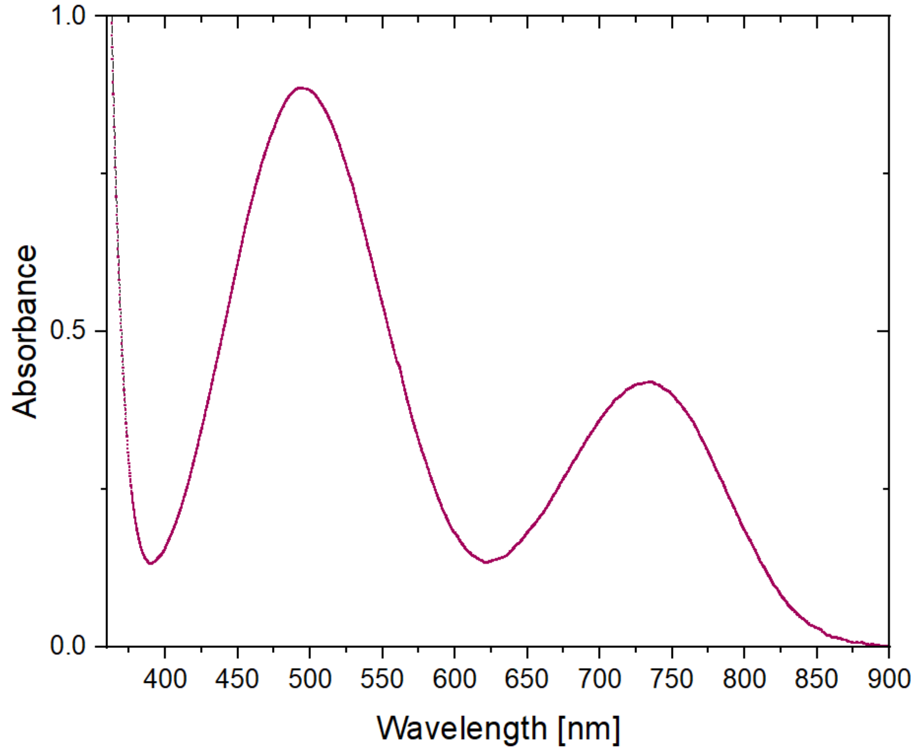

Fig. S13. UV-vis absorption spectrum of **2** in benzene at 23 °C (l_1 = 517 nm, l_2 = 736 nm).

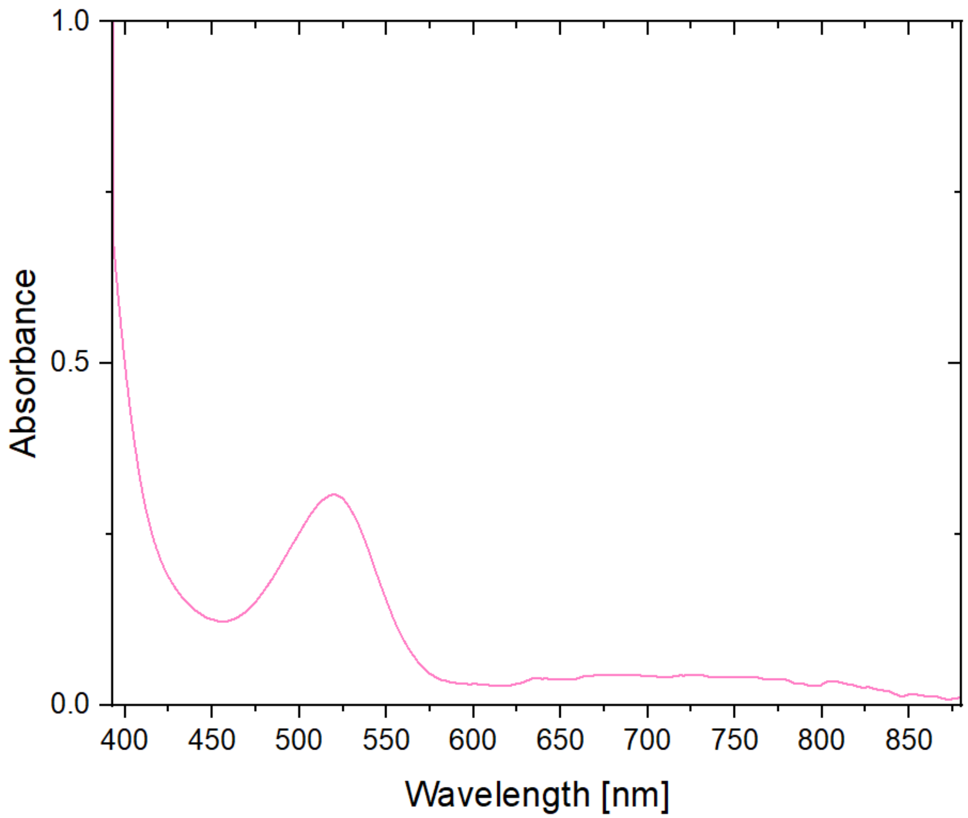

Fig. S14. UV-vis absorption spectrum of **3** in benzene at 23 °C (l_1 = 522 nm, l_2 = 731 nm).

FT-IR Spectra

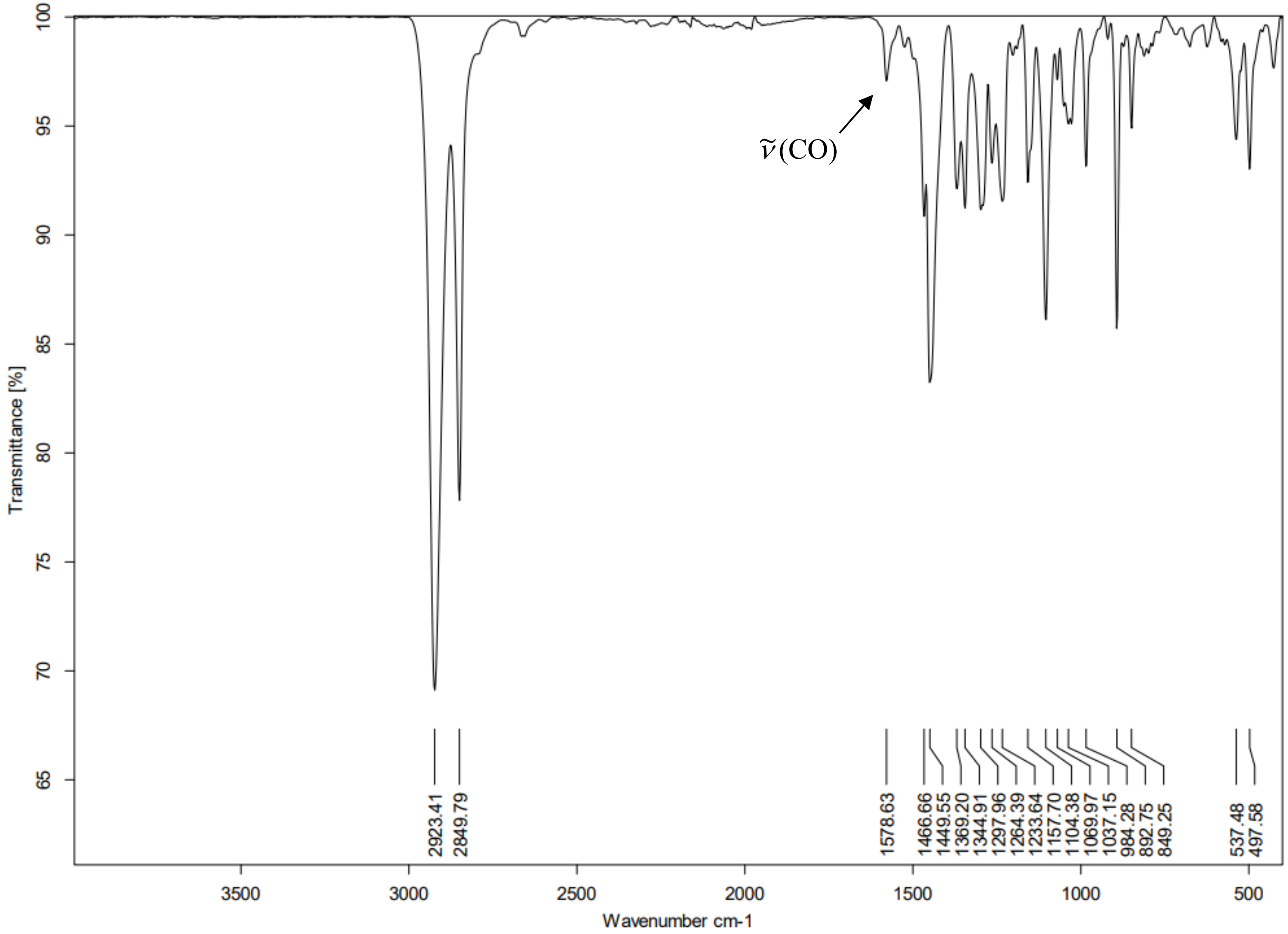

Fig. S15. Solid-state FT-IR spectrum of **2**.

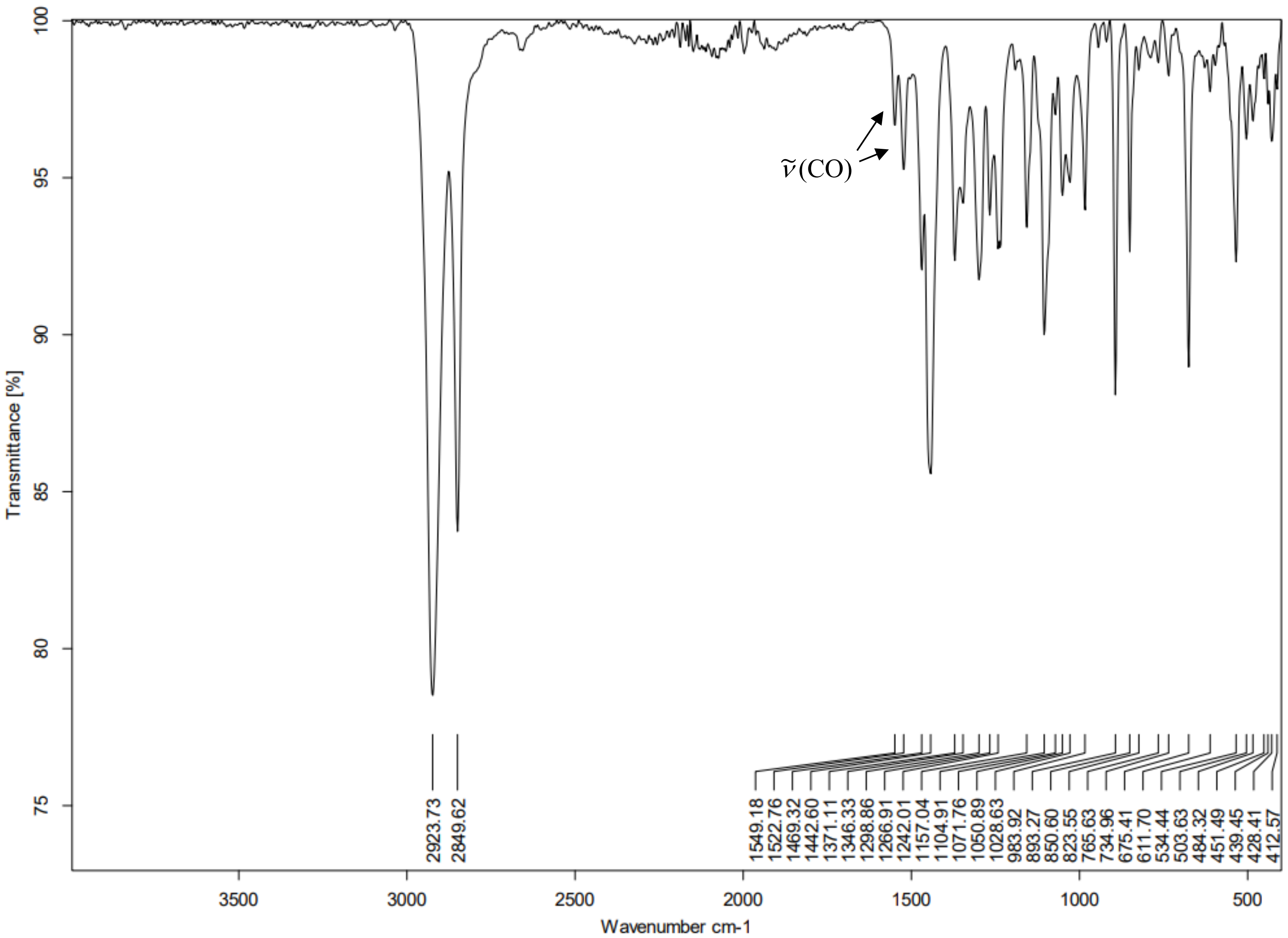

Fig. S16. Solid-state FT-IR spectrum of **3**.

EPR Spectrum

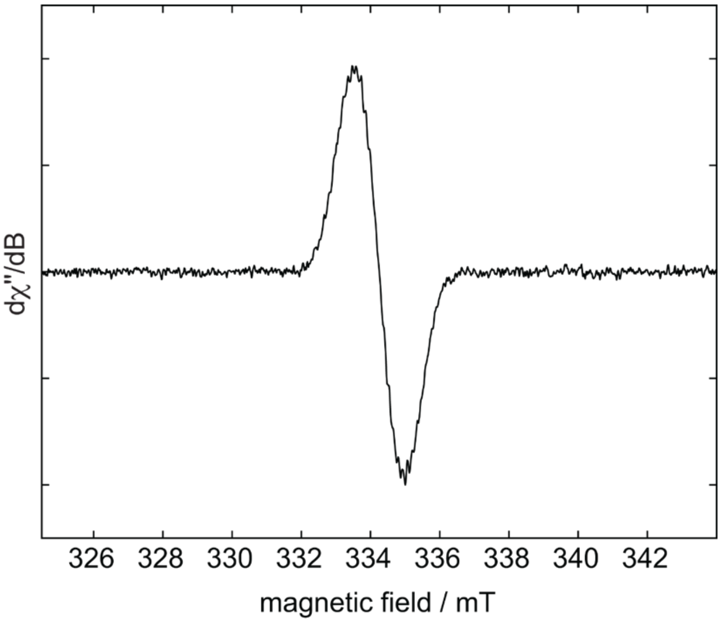

Fig. S17. Continuous-wave X-band EPR spectrum of the radical anion K[**2**]. The broad, unresolved signal is centered around a *g* value of 2.0047.

Cyclic Voltammetry

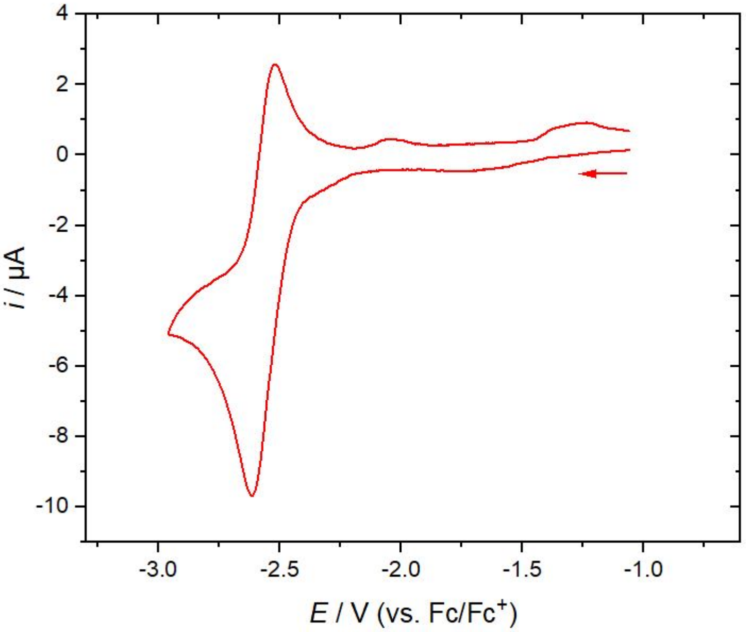

Fig. S18. Cyclic voltammogram of **2** in THF/0.1 M [nBu$_4$N][PF$_6$] measured at 250 mV s^{-1}. Formal potential: $E_{1/2} = -2.56$ V.

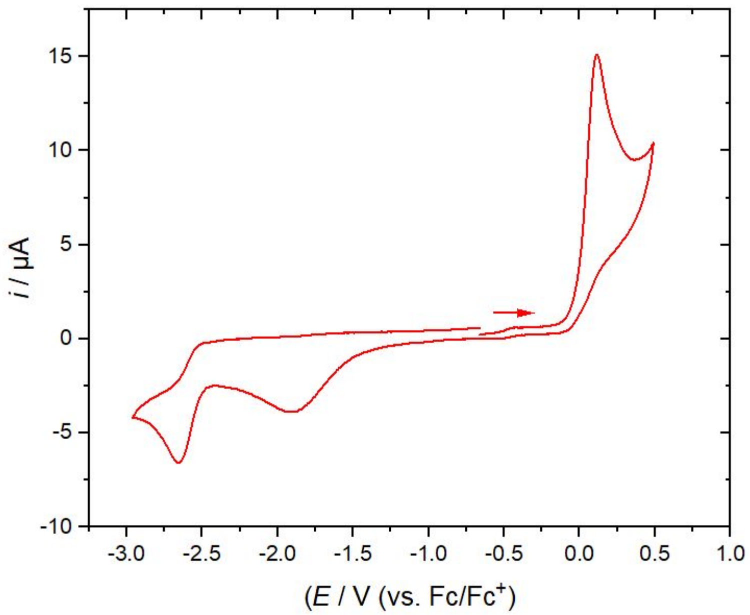

Fig. S19. Cyclic voltammogram of **2** in THF/0.1 M [nBu$_4$N][PF$_6$] measured at 250 mV s^{-1}. Formal potential: $E_{pa} = 0.12$ V.

Single Crystal X-Ray Diffraction Data

General: The crystallographic data of **2**, [K(dme)$_2$][**2**], [K(dme)]$_2$[**2**], **3**, and **4** were collected on a *XtaLAB Synergy Dualflex HyPix* diffractometer with a Hybrid Pixel array detector and multi-layer mirror monochromated $Cu_{K\alpha}$ or $Mo_{K\alpha}$ radiation. The structures were solved using the intrinsic phasing method (*51*), refined with the ShelXL program (*52*) and expanded using Fourier techniques. All non-hydrogen atoms were refined anisotropically. Hydrogen atoms were included in structure factor calculations. All hydrogen atoms were assigned to idealized geometric positions.

CCDC 2444752-2444756 contain the supplementary crystallographic data for this paper. These data can be obtained free of charge via www.ccdc.cam.ac.uk/data_request/cif, or by emailing data_request@ccdc.cam.ac.uk, or by contacting The Cambridge Crystallographic Data Centre, 12 Union Road, Cambridge CB2 1EZ, UK, fax: +44 1223 336033.

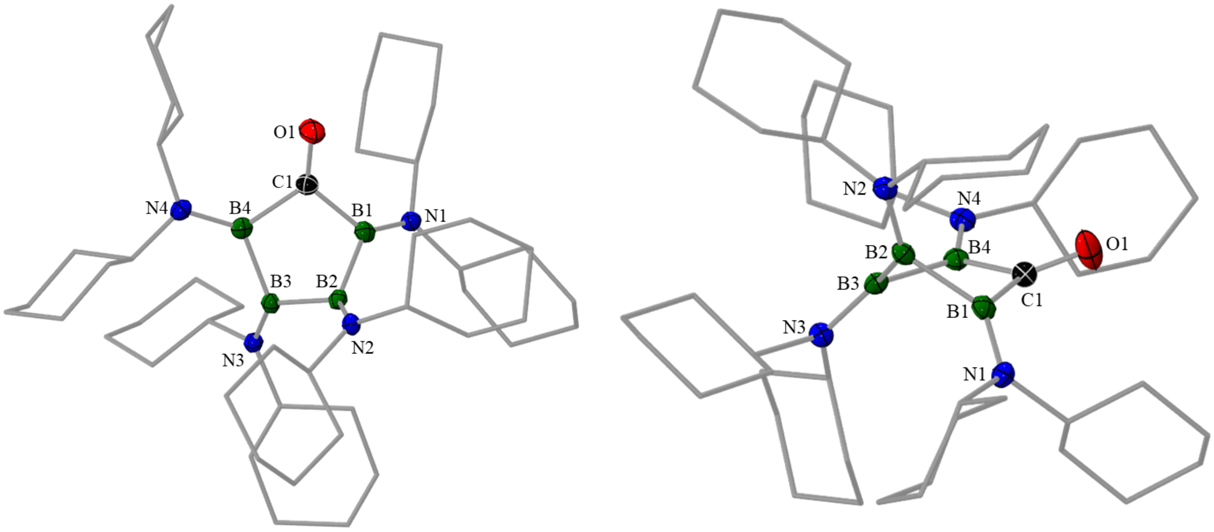

Fig. S20. Solid-state structure of **2**. Atomic displacement ellipsoids represented at 50%. Ellipsoids of ligand periphery and hydrogen atoms omitted for clarity.

Crystal data for **2**: $C_{52}H_{91}B_4N_4O$, M_r = 831.52, orange block, 0.100×0.090×0.060 mm^3, triclinic space group $P\bar{1}$, a = 10.41740(10) Å, b = 14.14150(10) Å, c = 18.0440(2) Å, α = 85.2360(10)°, β = 85.5590(10)°, γ = 76.6790(10)°, V = 2573.08(4) Å^3, Z = 2, ρ_{calcd} = 1.073 g·cm^{-3}, μ = 0.458 mm^{-1}, F(000) = 918, T = 100(2) K, R_1 = 0.0409, wR_2 = 0.0975, 9362 independent reflections [2θ≤136.484°] and 550 parameters.

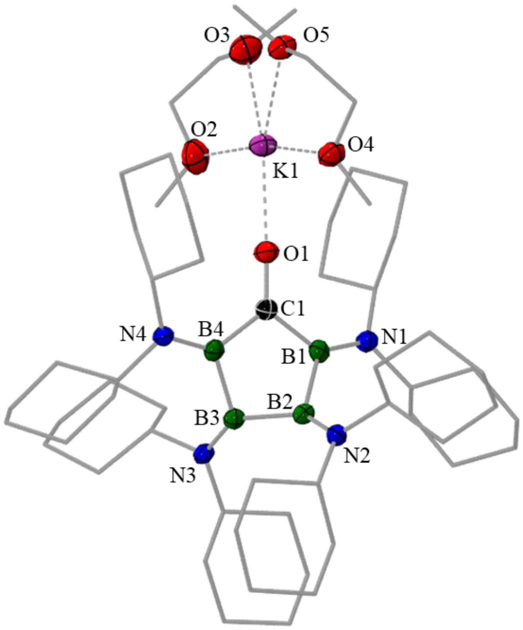

Fig. S21. Solid-state structure of [K(dme)$_2$][**2**]. Atomic displacement ellipsoids represented at 50%. Ellipsoids of ligand periphery and hydrogen atoms omitted for clarity.

Crystal data for [K(dme)$_2$][**2**]: $C_{57}H_{108}B_4KN_4O_5$, M_r = 1011.81, clear light yellow plate, 0.260×0.100×0.020 mm^3, triclinic space group $P\bar{1}$, a = 12.5666(2) Å, b = 13.1416(3) Å, c = 20.1304(4) Å, α = 94.074(2)°, β = 98.1530(10)°, γ = 112.054(2)°, V = 3021.93(11) Å^3, Z = 2, ρ_{calcd} = 1.112 g·cm^{-3}, μ = 1.123 mm^{-1}, F(000) = 1114, T = 100(2) K, R_1 = 0.0602, wR_2 = 0.1337, 10968 independent reflections [2θ≤136.498°] and 644 parameters.

One cyclohexyl moiety showed disorder. The atomic displacement parameters (ADPs) of overlapping atoms from different PARTs (C1 to C6 of RESIs 9/91) were restrained using similarity restraint (SIMU) and rigid body restraint (RIGU). The 1-2 and 1-3 distances of the disordered cyclohexyl moiety were restrained using the same distance restraint (SAME). The N6_1-C1_9/91 distances were restrained using the same distance restraint (SADI).

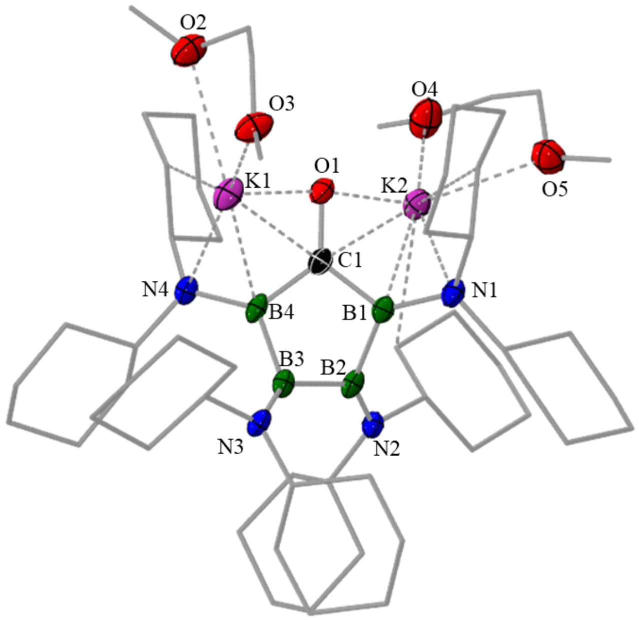

Fig. S22. Solid-state structure of [K(dme)]$_2$[**2**]. Atomic displacement ellipsoids represented at 50%. Ellipsoids of ligand periphery and hydrogen atoms omitted for clarity.

Crystal data for [K(dme)]$_2$[**2**]: $C_{57}H_{108}B_4K_2N_4O_5$, M_r = 1050.91, dark yellow plate, 0.250×0.200×0.030 mm^3, monoclinic space group $P2_1/n$, a = 13.8416(2) Å, b = 21.2206(2) Å, c = 21.5202(2) Å, β = 107.0550(10)°, V = 6043.09(12) Å^3, Z = 4, ρ_{calcd} = 1.155 g·cm^{-3}, μ = 1.746 mm^{-1}, F(000) = 2304, T = 100(2) K, R_1 = 0.0593, wR_2 = 0.1636, 11464 independent reflections [2θ≤140.136°] and 708 parameters.

Refined as a 2-component inversion twin. The BASF parameter was refined to 44.9%.

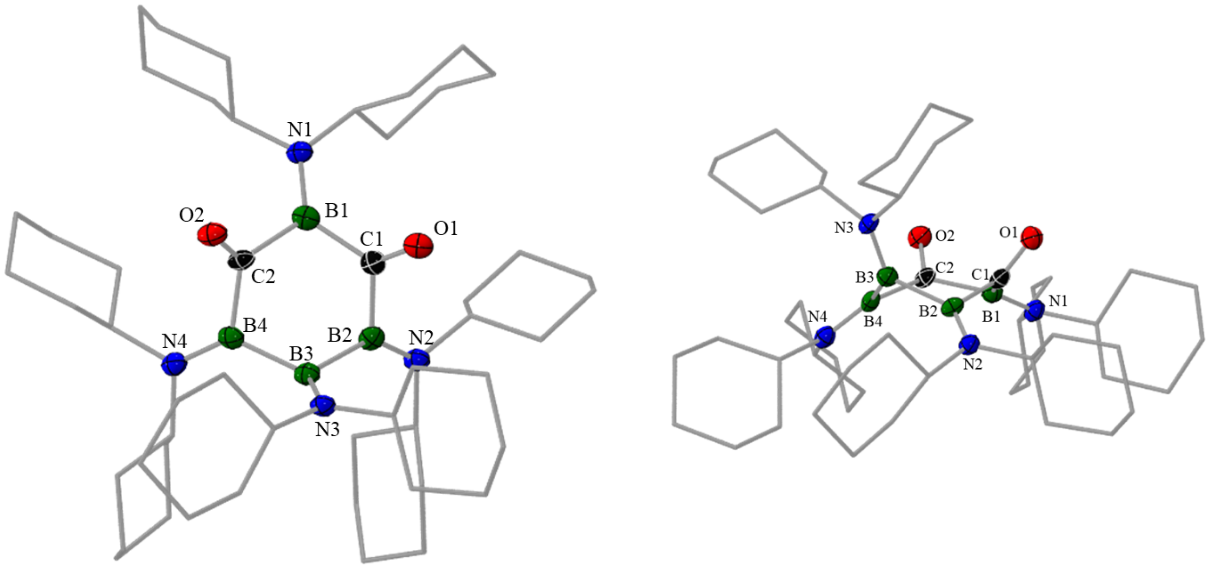

Fig. S23. Solid-state structure of **3**. Atomic displacement ellipsoids represented at 50%. Ellipsoids of ligand periphery and hydrogen atoms omitted for clarity.

Crystal data for **3**: $C_{52}H_{92}B_4Cl_4N_4O_2$, M_r = 990.33, colorless plate, 0.180×0.100×0.070 mm^3, triclinic space group $P1$, a = 10.5996(2) Å, b = 11.5999(4) Å, c = 13.2712(3) Å, α = 101.949(2)°, β = 113.172(2)°, γ = 101.513(2)°, V = 1394.11(7) Å^3, Z = 1, ρ_{calcd} = 1.180 g·cm^{-3}, μ = 2.235 mm^{-1}, F(000) = 536, T = 100(2) K, R_1 = 0.0637, wR_2 = 0.1363, Flack parameter = 0.45(2), 7832 independent reflections [2θ≤136.496°] and 596 parameters.

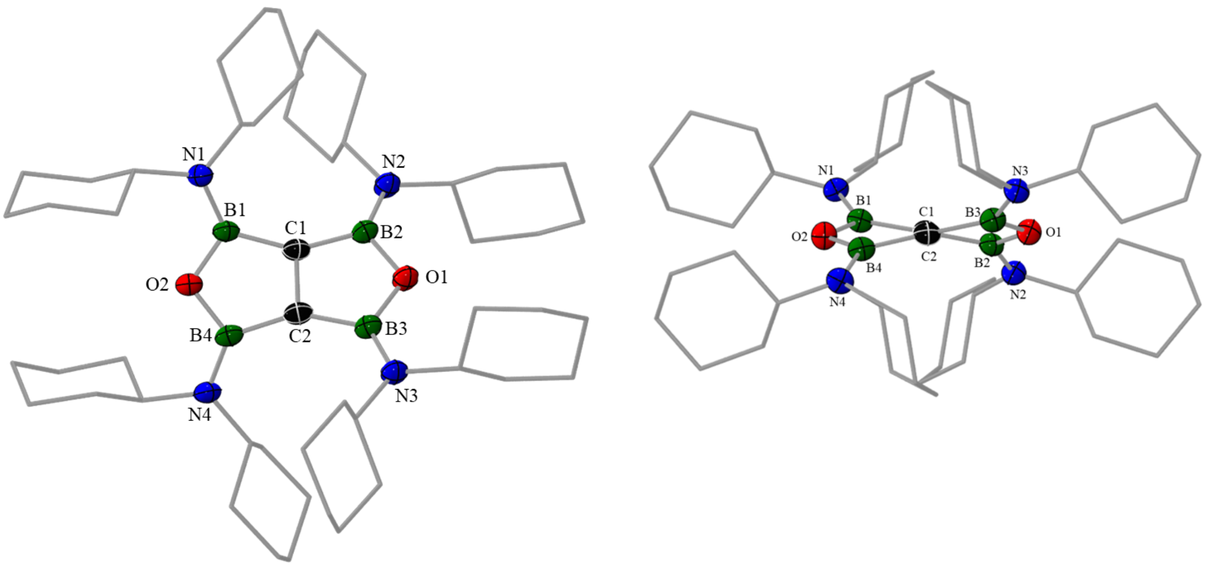

Fig. S24. Solid-state structure of **4**. Atomic displacement ellipsoids represented at 50%. Ellipsoids of ligand periphery and hydrogen atoms omitted for clarity.

Crystal data for **4**: $C_{50}H_{88}B_4N_4O_2$, M_r = 820.48, light colorless plate, 0.220×0.090×0.040 mm^3, monoclinic space group $P2_1/n$, a = 10.16070(10) Å, b = 22.0468(3) Å, c = 28.0695(5) Å, β = 95.6420(10)°, V = 6257.41(15) Å^3, Z = 4, ρ_{calcd} = 0.871 g·cm^{-3}, μ = 0.385 mm^{-1}, F(000) = 1808, T = 100(2) K, R_1 = 0.0926, wR_2 = 0.2091, 11458 independent reflections [2θ≤136.5°] and 541 parameters.

The unit cell contains solvent molecules which have been treated as a diffuse contribution to the overall scattering without specific atom positions by SQUEEZE/PLATON (A.L. Spek, *Acta Cryst.*, **2015**, *C71*, 9–18).

Computational Details

All computations were performed using the *Gaussian16 (Revision C.01)* package (*53*). All structures were fully optimized without symmetry constraints at the ωB97xd level of theory employing def2-SVPP basis sets for all atoms (*54,55*). Zero-point vibrational energies and thermal corrections were computed from frequency calculations with a standard state of 298 K and 1 atm; thermal free energies (ΔE_{298}) were obtained from these single-point frequency calculations. The presence of true energy minima on the potential energy surface was verified for all optimized species by the absence of imaginary frequencies. Predicted vibrational C=O frequencies were determined by applying a scaling factor of 0.91293, which was obtained by calculating the C=O stretching vibrations for a "training set" of ten different molecules (4 boron compounds/ 6 standard organics) at this level of theory and comparison to their experimental values (see Supplementary Materials for specifics of the "training set"). For EPR, TD-DFT, and orbital calculations, def2-TZVP basis sets were used, combined with the SMD solvation model (scrf=smd) for inclusion of tetrahydrofuran solvent effects (*56*). Illustrations of optimized structures, as well as orbital and spin density plots were prepared with *IQmol 3.1.3* (*57*).

The scaling factor used in the prediction of the vibrational C=O frequencies was determined to a value of 0.91293 (linear regression line through origin; R^2 = 0.99991) by calculating the C=O stretching vibrations $\tilde{\nu}(CO)_{calc}$ for a "training set" of the following ten different molecules at the ωB97xd/def2-SVPP level of theory and comparison to their experimental values $\tilde{\nu}(CO)_{exp}$.

Table S1. Experimental ($\tilde{\nu}(CO)_{exp}$) and calculated ($\tilde{\nu}(CO)_{calc}$) C=O stretching vibrations (in cm^{-1}) for a selected "training set" of molecules, as well as for **2** and **3** (scaled values given in *italics*).

	$\tilde{\nu}(CO)_{calc}$	$\tilde{\nu}(CO)_{exp}$
TrippB$(CO)_2$ (*58*)	2116, 2225	1942, 2060
cAAC(CO)B-B(CO)cAAC (*59*)	2095, 2105	1928
C_2(IDip·BC(O)O$)_2$ (*60*)	1824	1691
(IMes·BBr$)_2$C(O)O (*40*)	1863	1693
acetaldehyde	1896	1727
acetone	1886	1715
benzophenone	1824	1655
cinnamaldehyde	1865	1678
cyclohexanone	1884	1716
succinimide	1906, 1949	1708, 1774
**2**	1737(*1586*)	1578
**3**	1686 (1539),<br>1714 (*1565*)	1522,<br>1549

Table S2. Isotropic Fermi contact coupling constants a_{iso} for the boron and carbonyl centers of [**2**]$^{\bullet-}$ in Hz (SMD(dimethoxyethane)).

	**B(1)**	**B(2)**	**B(3)**	**B(4)**	**C**	**O**
a_{iso}	-8.2	-16.2	-13.1	-10.5	-17.8	-22.1

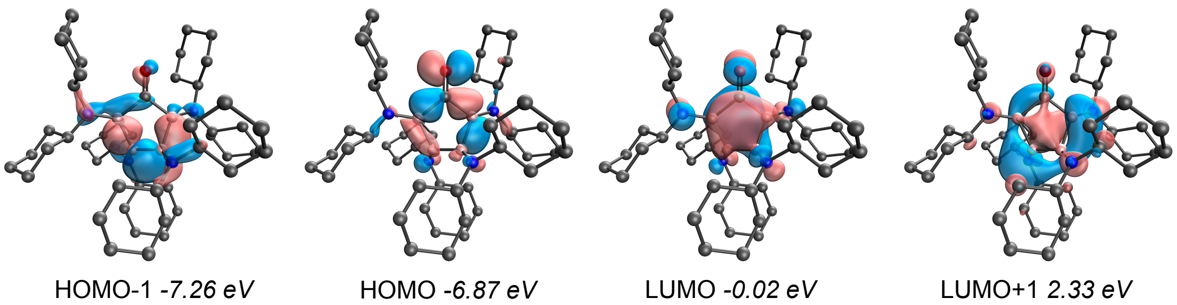

Fig. S25. Selected molecular orbitals of **2** illustrating important bonding interactions within the B_4C ring. Isosurface plots at 0.04 a.u.; calculated *Eigenvalues* in italics.

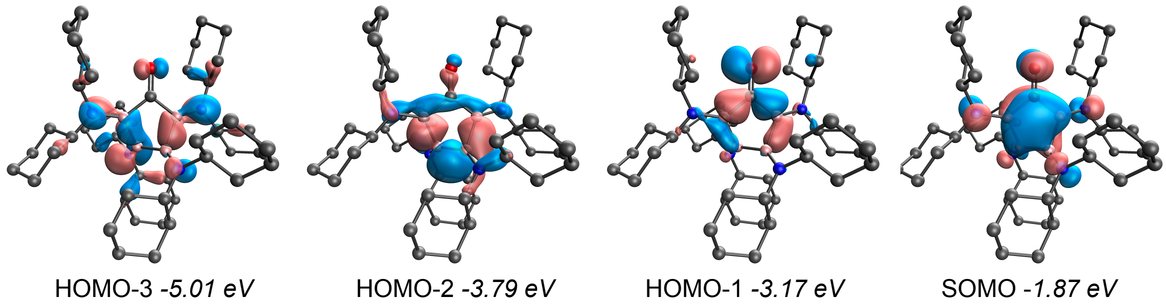

Fig. S26. Selected molecular orbitals of [**2**]$^{\bullet-}$ illustrating important bonding interactions within the monoradical anionic B_4C ring. Isosurface plots at 0.04 a.u.; calculated *Eigenvalues* in italics.

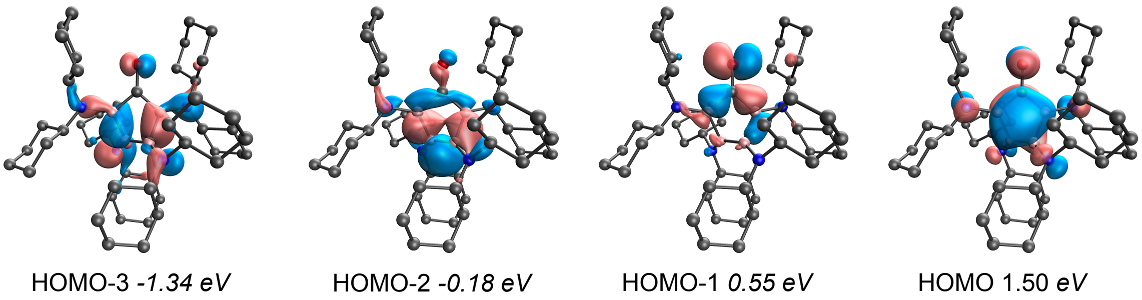

Fig. S27. Selected molecular orbitals of [**2**]$^{2-}$ illustrating important bonding interactions within the dianionic B_4C ring. Isosurface plots at 0.04 a.u.; calculated *Eigenvalues* in italics.

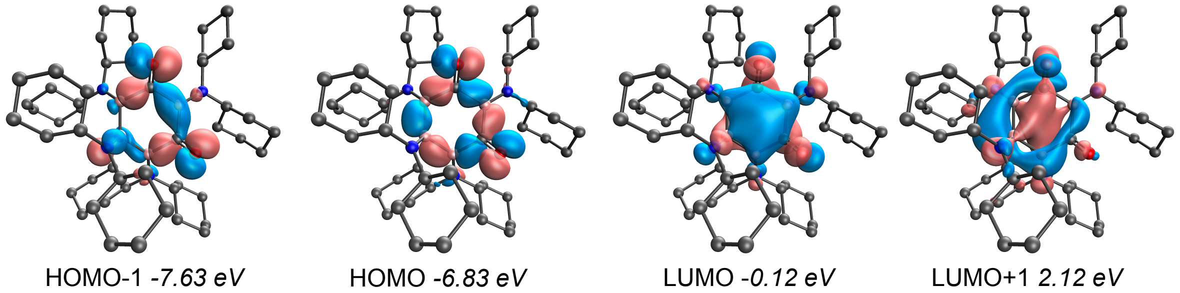

Fig. S28. Selected molecular orbitals of **3** illustrating important bonding interactions within the B_4C_2 ring. Isosurface plots at 0.04 a.u.; calculated *Eigenvalues* in italics.

Cartesian Coordinates of DFT Optimized Structures

**2**	B	-1.22153100	0.95063300	0.16246200
	B	-0.65190400	-0.59659200	-0.37265300
	B	0.72734400	-0.63948300	0.59492200
	B	1.34919300	0.84185700	-0.05177100
	N	-2.52291000	1.25246200	0.60403200
	N	-1.01041600	-1.22823200	-1.58577300
	N	1.14600200	-1.11946100	1.84877900
	N	2.46108100	1.08614500	-0.86871100
	C	0.15436100	1.86192700	0.35142800
	O	0.27006200	2.96735500	0.87847000
	C	-2.94870800	2.52551000	1.20633900
	H	-4.03970000	2.46142600	1.35931900
	C	-2.72884400	3.71624800	0.26670800
	H	-3.25855100	3.52203300	-0.68323900
	H	-1.65397500	3.80250200	0.04523700
	C	-3.21861200	5.01948000	0.90156900
	H	-3.00550100	5.86745100	0.22720200
	H	-4.31944900	4.98361100	1.02380100
	C	-2.56894100	5.24891100	2.26829900
	H	-1.47779200	5.36677200	2.13358600
	H	-2.94401500	6.18539300	2.71775100
	C	-2.83120900	4.06791200	3.20569000
	H	-3.91858300	4.00001900	3.40918400
	H	-2.33992200	4.23483200	4.18041800
	C	-2.33858000	2.75422000	2.59538000
	H	-1.24282100	2.78318400	2.50052400
	H	-2.59913300	1.90197800	3.24758600
	C	-3.57711500	0.23381200	0.51139300
	H	-3.09560300	-0.65862300	0.07902700
	C	-4.72090300	0.62283500	-0.43746700
	H	-5.23577800	1.52589600	-0.06089700
	H	-4.30459400	0.88513100	-1.42458500
	C	-5.74123500	-0.51172600	-0.57061900
	H	-5.26229800	-1.37642500	-1.06657300
	H	-6.57102400	-0.19617100	-1.22683300
	C	-6.27572500	-0.96026700	0.79112800
	H	-6.96933300	-1.81024000	0.66736200
	H	-6.86105000	-0.13662000	1.24326200
	C	-5.13136800	-1.33522400	1.73526200
	H	-4.60287200	-2.22415900	1.33876100
	H	-5.52642200	-1.62025300	2.72600200
	C	-4.13741800	-0.18187200	1.87931900
	H	-3.30615500	-0.46480400	2.54303700
	H	-4.64997300	0.67408600	2.35641900
	C	-1.78211500	-0.49427000	-2.59321200
	H	-2.20163800	0.37819600	-2.06712600
	C	-2.96950800	-1.25467800	-3.19691300
	H	-3.63632000	-1.59293500	-2.38616200
	H	-2.62303100	-2.16159900	-3.72569700
	C	-3.73890200	-0.37683500	-4.18836000
	H	-4.57183900	-0.95108800	-4.63006700

H	-4.19709200	0.47293000	-3.64757700
C	-2.82244000	0.16618400	-5.28692500
H	-2.44347600	-0.67848300	-5.89409600
H	-3.39179600	0.81769000	-5.97251100
C	-1.63722300	0.92765600	-4.68920600
H	-2.01045900	1.83057900	-4.16891100
H	-0.96274500	1.28228000	-5.48809900
C	-0.86500900	0.06215300	-3.69266900
H	-0.37804800	-0.77427600	-4.23093100
H	-0.05985800	0.64468000	-3.21111800
C	-0.54643100	-2.55823000	-1.99406700
H	-0.71558100	-2.65543300	-3.08110200
C	-1.37381600	-3.65941500	-1.31609500
H	-1.26953200	-3.54510900	-0.22265800
H	-2.44223500	-3.50438200	-1.54327200
C	-0.93754300	-5.06081100	-1.74755400
H	-1.51160500	-5.82279600	-1.19153200
H	-1.18470400	-5.20212400	-2.81760900
C	0.56488800	-5.27639400	-1.55142300
H	0.85892600	-6.27180500	-1.92784000
H	0.79929000	-5.26643800	-0.47050800
C	1.37513100	-4.18386000	-2.25313700
H	1.23202200	-4.27154900	-3.34770400
H	2.45395500	-4.32905200	-2.06719600
C	0.95207300	-2.78726400	-1.79417800
H	1.21118800	-2.65799100	-0.73024500
H	1.50084700	-2.01248300	-2.35102200
C	0.43131800	-2.15110600	2.60950700
H	0.82753000	-2.14161000	3.64025600
C	0.67603300	-3.55442800	2.04828200
H	0.33210600	-3.56425800	1.00233600
H	1.75990600	-3.76169200	2.02732000
C	-0.06640000	-4.63190500	2.84059800
H	0.09061100	-5.61883300	2.37025600
H	0.36120500	-4.69841000	3.85963800
C	-1.56200600	-4.32257700	2.94433100
H	-2.07021400	-5.08941600	3.55478600
H	-2.01510400	-4.36958800	1.93531000
C	-1.79730200	-2.92981300	3.53298500
H	-1.44063900	-2.91151800	4.58094600
H	-2.87790100	-2.70340400	3.56503800
C	-1.06591600	-1.85926300	2.72345600
H	-1.49774700	-1.81259800	1.70767900
H	-1.19891400	-0.85827500	3.16976600
C	2.29035300	-0.50430800	2.52866800
H	2.74457100	0.17076900	1.78599400
C	3.38638900	-1.49955600	2.92975600
H	2.99297400	-2.23504700	3.65660800
H	3.70108900	-2.06761700	2.03561200
C	4.58359300	-0.77698100	3.55387900
H	5.34400200	-1.51160500	3.87098300
H	5.06398700	-0.13944200	2.78721700
C	4.15873000	0.09844800	4.73517100
H	5.03284800	0.63229000	5.14757400

	H	3.77187100	-0.54904800	5.54622300
	C	3.07176500	1.09395000	4.32387300
	H	3.48761000	1.80342800	3.58312000
	H	2.75324000	1.69774500	5.19116900
	C	1.86580500	0.38133100	3.70795400
	H	1.36737600	-0.23023000	4.48398100
	H	1.12468800	1.11607200	3.34750400
	C	3.37693100	-0.00611100	-1.20361900
	H	2.92373700	-0.91351900	-0.77172200
	C	4.76084700	0.14484000	-0.55781900
	H	4.64082000	0.29633200	0.52845400
	H	5.26724800	1.04586800	-0.95245500
	C	5.62764900	-1.08569600	-0.83462700
	H	6.62683200	-0.95501700	-0.38395400
	H	5.17215500	-1.96577700	-0.34102200
	C	5.75077700	-1.35702500	-2.33583400
	H	6.34229300	-2.27265900	-2.51307500
	H	6.30635800	-0.52491400	-2.80948400
	C	4.37442400	-1.47984200	-2.99487500
	H	3.86368800	-2.38044000	-2.60468500
	H	4.48139700	-1.62665000	-4.08361700
	C	3.50940900	-0.24876200	-2.71355700
	H	2.50384400	-0.36167700	-3.15462900
	H	3.97025700	0.63006200	-3.20111300
	C	2.83222600	2.41518100	-1.37525700
	H	3.74220200	2.29150700	-1.98782000
	C	3.19827300	3.38063800	-0.24000600
	H	4.02380600	2.94109000	0.34835500
	H	2.32913000	3.47950600	0.43158200
	C	3.59673700	4.75481100	-0.78117700
	H	3.80805100	5.44006300	0.05814800
	H	4.53570900	4.67091200	-1.36356000
	C	2.50101500	5.33503600	-1.67842300
	H	1.58924500	5.50034700	-1.07410200
	H	2.80842700	6.31938000	-2.07360000
	C	2.17450200	4.38188100	-2.83026900
	H	3.06401000	4.28321100	-3.48285700
	H	1.36840700	4.79732000	-3.45998600
	C	1.76895300	3.00034000	-2.31253500
	H	0.80830000	3.08145100	-1.77575100
	H	1.61148600	2.30223900	-3.15355400
**[2]$^{\bullet-}$**	B	1.15925300	0.97335500	-0.22816900
	B	0.69897300	-0.55868300	0.38945200
	B	-0.67922700	-0.65046000	-0.52126500
	B	-1.29678800	0.80566200	0.15351100
	N	2.48188600	1.31580300	-0.70144600
	N	1.22390000	-1.16481300	1.58279800
	N	-1.19714900	-1.24880300	-1.71389100
	N	-2.55169300	0.98600200	0.83585700
	C	-0.16203800	1.85398800	-0.18527700

O	-0.28528800	3.11416300	-0.36395200
C	2.83335900	2.61608500	-1.26712100
H	3.89129900	2.57240600	-1.59166700
C	2.74455200	3.73794500	-0.22312700
H	3.40538600	3.48760600	0.62733900
H	1.70177200	3.76673400	0.13551400
C	3.11731600	5.09810100	-0.81593200
H	2.99835000	5.88884200	-0.05247400
H	4.18702100	5.10299800	-1.11187700
C	2.25844700	5.41534000	-2.04283600
H	1.20084000	5.47931300	-1.72853100
H	2.53970500	6.39694400	-2.46888400
C	2.39177700	4.31561300	-3.09939800
H	3.43823900	4.29223000	-3.46801100
H	1.75525600	4.54628200	-3.97340500
C	2.01212400	2.95120200	-2.52100500
H	0.94827500	2.95965400	-2.23502200
H	2.16150300	2.15259500	-3.27042000
C	3.53296300	0.31132200	-0.72324400
H	3.09281000	-0.59336000	-0.27005400
C	4.76430500	0.66637500	0.12905500
H	5.25388100	1.57322400	-0.27465100
H	4.43604600	0.91392900	1.15223300
C	5.77883100	-0.48046200	0.15599300
H	5.32500600	-1.35248500	0.66436900
H	6.66479300	-0.19541900	0.75237900
C	6.19842500	-0.90052500	-1.25477300
H	6.89314800	-1.75924000	-1.20912300
H	6.75198700	-0.06828700	-1.73207200
C	4.97737000	-1.24188900	-2.11241300
H	4.47630800	-2.13650200	-1.69321300
H	5.29034400	-1.50834900	-3.13858200
C	3.98188500	-0.08102900	-2.14228800
H	3.09416800	-0.34369500	-2.73745300
H	4.45475700	0.78665300	-2.64117400
C	1.97647300	-0.32671500	2.51082700
H	2.32829600	0.52917600	1.91330000
C	3.22673300	-0.97500600	3.12349400
H	3.88651500	-1.32707200	2.31189300
H	2.95309100	-1.86476100	3.72192700
C	3.97498800	0.00835700	4.02855200
H	4.85522700	-0.48456400	4.48021300
H	4.36272100	0.84449900	3.41604400
C	3.06287000	0.57571100	5.11913400
H	2.74808900	-0.24672400	5.79165100
H	3.61766000	1.30050800	5.74270900
C	1.82046400	1.23131100	4.51128600
H	2.12963200	2.11121600	3.91539400
H	1.15489100	1.60948000	5.30812600
C	1.06600700	0.26032000	3.60219500
H	0.63873700	-0.56049600	4.21270600
H	0.22274000	0.76930500	3.10258500
C	0.83064200	-2.47683000	2.08371600
H	1.07870000	-2.52591300	3.16110100

C	1.64000700	-3.58992400	1.39953700
H	1.46414400	-3.51802800	0.31180500
H	2.71591000	-3.39536500	1.55254400
C	1.27318300	-4.98615500	1.90636800
H	1.82853400	-5.75629300	1.34023400
H	1.59177200	-5.08247900	2.96339900
C	-0.23301800	-5.24426600	1.81945800
H	-0.47624000	-6.23574100	2.24380400
H	-0.53820500	-5.27083600	0.75650000
C	-1.02127200	-4.14768400	2.54018100
H	-0.79489300	-4.19447600	3.62417800
H	-2.10627700	-4.33084200	2.43990100
C	-0.67311200	-2.75819900	2.00276100
H	-1.00938100	-2.66814200	0.95717200
H	-1.20617600	-1.97569200	2.56573600
C	-0.49444500	-2.28730400	-2.46309300
H	-0.95776600	-2.36294800	-3.46470300
C	-0.64163200	-3.66474000	-1.80861900
H	-0.23830100	-3.59128100	-0.78692700
H	-1.71460300	-3.90768400	-1.70896500
C	0.09625300	-4.76443100	-2.57471100
H	0.01539100	-5.72431500	-2.03189800
H	-0.39079700	-4.91716500	-3.55831700
C	1.56734100	-4.40375700	-2.80020200
H	2.06985400	-5.19236800	-3.38980900
H	2.08447100	-4.35917900	-1.82252300
C	1.70027600	-3.04605400	-3.49392100
H	1.27041400	-3.11692500	-4.51307900
H	2.76589900	-2.78134700	-3.61850600
C	0.98043300	-1.95351200	-2.70434500
H	1.47456200	-1.81504900	-1.72693700
H	1.04056200	-0.98004700	-3.22020700
C	-2.30527400	-0.60407600	-2.40995100
H	-2.74276500	0.09425100	-1.68023700
C	-3.43210500	-1.56101100	-2.82673200
H	-3.05128700	-2.32433800	-3.53228800
H	-3.78782100	-2.10303600	-1.93181400
C	-4.58897400	-0.80993500	-3.49292200
H	-5.36603000	-1.52289200	-3.82414800
H	-5.06883600	-0.14631600	-2.74876000
C	-4.10684500	0.04035100	-4.67098500
H	-4.95481400	0.59421700	-5.11383700
H	-3.71451200	-0.62706900	-5.46377400
C	-3.00242600	1.00727900	-4.23682000
H	-3.41619900	1.72855500	-3.50643200
H	-2.64909600	1.60053100	-5.09928700
C	-1.83451500	0.26185500	-3.58895600
H	-1.33786700	-0.37089900	-4.35093000
H	-1.07938900	0.97225800	-3.20936600
C	-3.37936300	-0.16238200	1.15985800
H	-2.84808300	-1.03750900	0.74828100
C	-4.76913500	-0.14683500	0.50136700
H	-4.65217400	0.00335300	-0.58420200
H	-5.35752300	0.71091900	0.87973600

C	-5.53182800	-1.44362900	0.78185400
H	-6.53330500	-1.40988600	0.31538700
H	-4.99221800	-2.28738700	0.30970100
C	-5.65352900	-1.70755800	2.28492500
H	-6.16855900	-2.66861400	2.46692400
H	-6.28397700	-0.91825900	2.73941900
C	-4.28021800	-1.70152500	2.96181300
H	-3.68777700	-2.55701900	2.58497800
H	-4.38940300	-1.84990400	4.05157200
C	-3.52096100	-0.40503800	2.67245800
H	-2.51501600	-0.42675000	3.12491200
H	-4.05909800	0.44172900	3.13993000
C	-3.04465400	2.28480100	1.28813000
H	-4.00874400	2.12266700	1.80847600
C	-3.33213700	3.22111600	0.10572100
H	-4.06570700	2.73464700	-0.56398300
H	-2.38150800	3.34861400	-0.44257600
C	-3.84480800	4.58581100	0.56868100
H	-3.99447600	5.24858000	-0.30328200
H	-4.83596400	4.47530600	1.05589200
C	-2.87069800	5.22880100	1.55922200
H	-1.90692200	5.40243000	1.04676500
H	-3.25137100	6.21196800	1.89448300
C	-2.63175000	4.31073000	2.76058500
H	-3.58386200	4.18604000	3.31647000
H	-1.91603200	4.77675000	3.46240300
C	-2.10926700	2.94473000	2.31196200
H	-1.12021500	3.06897400	1.84386700
H	-1.99097100	2.26753500	3.17741300

**[2]**$^{2-}$	B	-0.98836000	1.09584100	0.16829900
	B	-0.71786200	-0.47204800	-0.40520900
	B	0.54922400	-0.61743200	0.58322400
	B	1.26466300	0.64928500	-0.28904800
	N	-2.33623600	1.62728300	0.51901700
	N	-1.49909900	-1.13582400	-1.47416600
	N	1.18033200	-1.26515800	1.74312100
	N	2.60009100	0.60419300	-0.93012900
	C	0.37504100	1.84548100	0.09584200
	O	0.67473300	3.10629600	0.26284000
	C	-2.52734900	2.97438800	1.02610100
	H	-3.59073600	3.09360100	1.32803400
	C	-2.25337400	4.02320600	-0.06399900
	H	-2.91570600	3.82104500	-0.92655000
	H	-1.20012200	3.88070900	-0.37022300
	C	-2.43962300	5.45450400	0.44410600
	H	-2.19685300	6.17915500	-0.35709700
	H	-3.50263300	5.62955100	0.71824000
	C	-1.55936300	5.71431800	1.66912400
	H	-0.50239300	5.59317900	1.37071600
	H	-1.69571200	6.75205200	2.03613700

C	-1.86666300	4.70378500	2.77711400
H	-2.91259000	4.85128200	3.12386500
H	-1.21512100	4.89174600	3.65196500
C	-1.67172200	3.27259900	2.27179300
H	-0.61801200	3.13908000	1.96696100
H	-1.91214000	2.54027500	3.06404200
C	-3.42318900	0.69748500	0.70366400
H	-3.06771400	-0.25740700	0.27876700
C	-4.71396300	1.04585700	-0.06445500
H	-5.14118900	1.99408000	0.31722700
H	-4.46125400	1.21517900	-1.12493300
C	-5.76226100	-0.06354200	0.06570800
H	-5.37189000	-0.98136400	-0.41386900
H	-6.68847400	0.20701100	-0.47691100
C	-6.07916000	-0.37674900	1.53106600
H	-6.80382700	-1.21068200	1.59823400
H	-6.56531300	0.50573400	1.99238100
C	-4.80486900	-0.70535000	2.31325600
H	-4.36800800	-1.64291300	1.91763300
H	-5.04632000	-0.89388400	3.37709500
C	-3.77133500	0.41445000	2.17999600
H	-2.84307000	0.15314500	2.71069000
H	-4.16736100	1.33447400	2.65506300
C	-2.05351300	-0.25655200	-2.48680300
H	-2.24113800	0.69680700	-1.96507700
C	-3.40168200	-0.69334800	-3.08566400
H	-4.11721900	-0.88147000	-2.26612600
H	-3.29660100	-1.64444000	-3.64410500
C	-3.95012500	0.37018300	-4.04262000
H	-4.91344900	0.04030100	-4.47780000
H	-4.16069100	1.29435400	-3.47111900
C	-2.94854300	0.69411300	-5.15605200
H	-2.80972800	-0.20710300	-5.78733500
H	-3.35324900	1.48530300	-5.81687000
C	-1.59334200	1.11363500	-4.57839200
H	-1.71226800	2.06807000	-4.03125300
H	-0.87225200	1.30613100	-5.39549200
C	-1.04837200	0.06338000	-3.60891000
H	-0.80432900	-0.86559700	-4.16590600
H	-0.12054200	0.42050900	-3.12732900
C	-1.33433300	-2.52733500	-1.84214500
H	-1.75508800	-2.68437900	-2.85597900
C	-2.14027300	-3.43538800	-0.89838200
H	-1.78922200	-3.23406400	0.12825200
H	-3.19942200	-3.12245800	-0.93137000
C	-2.01090200	-4.92384100	-1.23059200
H	-2.54656900	-5.53470300	-0.47890300
H	-2.50294900	-5.12091900	-2.20516300
C	-0.54744200	-5.36496200	-1.32302500
H	-0.48660700	-6.42644900	-1.63175000
H	-0.08415700	-5.29887000	-0.32154300
C	0.23595000	-4.47864500	-2.29486100
H	-0.15965900	-4.63699000	-3.31967500
H	1.29725000	-4.78965000	-2.31855800

C	0.12578100	-2.99735200	-1.92717200
H	0.61638300	-2.79798900	-0.95972900
H	0.65250000	-2.37434900	-2.66834700
C	0.47472400	-2.21326600	2.58807400
H	0.99130600	-2.27374500	3.56750400
C	0.51283400	-3.62533800	1.98793100
H	0.07847400	-3.55494600	0.97856000
H	1.56722900	-3.92917700	1.85613300
C	-0.25113700	-4.66069900	2.81700400
H	-0.24874900	-5.64129200	2.30283900
H	0.26885100	-4.81149000	3.78536400
C	-1.68914000	-4.21371700	3.09574400
H	-2.20671500	-4.95633100	3.73352400
H	-2.24593300	-4.17021200	2.14068900
C	-1.71476800	-2.82930000	3.74799400
H	-1.24382100	-2.89793500	4.75063300
H	-2.75896000	-2.50312400	3.91062800
C	-0.97211200	-1.80077400	2.89476300
H	-1.49360700	-1.64929100	1.93365800
H	-0.95868200	-0.81418300	3.38789000
C	2.26281100	-0.52709800	2.37161000
H	2.71139200	0.06792800	1.56025200
C	3.39683800	-1.39550700	2.94098000
H	3.03029900	-2.02752000	3.77368400
H	3.75170000	-2.08242900	2.15107600
C	4.54931600	-0.52771700	3.45603200
H	5.34904300	-1.16057700	3.88759400
H	4.99925500	0.01347800	2.60208400
C	4.06717200	0.49823400	4.48721000
H	4.91189400	1.13365700	4.81683100
H	3.70467400	-0.03987000	5.38686300
C	2.93089900	1.35864000	3.92574000
H	3.31108200	1.96651900	3.08293600
H	2.57598400	2.07142200	4.69400400
C	1.77724700	0.49339900	3.41516300
H	1.30375600	-0.03519000	4.26890400
H	1.00597800	1.11961200	2.93242100
C	3.20084400	-0.66497700	-1.24635000
H	2.55659800	-1.42276000	-0.76729000
C	4.61343100	-0.87502300	-0.66514900
H	4.58411000	-0.67661700	0.41906200
H	5.31682200	-0.14117000	-1.10612700
C	5.13132300	-2.29064800	-0.93506100
H	6.15114600	-2.41895300	-0.52319500
H	4.48230900	-3.01498100	-0.40528100
C	5.12078200	-2.61462800	-2.43253700
H	5.46250100	-3.65367900	-2.60628200
H	5.84320000	-1.95095800	-2.94908400
C	3.72895500	-2.40103700	-3.03411600
H	3.02998200	-3.13336500	-2.58725500
H	3.74765100	-2.60571300	-4.12211700
C	3.20834900	-0.98900300	-2.75455600
H	2.18114100	-0.86446500	-3.13723600
H	3.84121200	-0.25303500	-3.28949500

	C	3.26049400	1.80480900	-1.40703800
	H	4.18961300	1.51423200	-1.94543500
	C	3.67742500	2.71065600	-0.23372800
	H	4.34927900	2.13983200	0.43620300
	H	2.74082500	2.95762600	0.30944700
	C	4.34828300	4.00418300	-0.70091400
	H	4.58952700	4.64507800	0.16951600
	H	5.31342300	3.77815800	-1.20589200
	C	3.44179000	4.76266200	-1.67469900
	H	2.50945800	5.03182900	-1.14460200
	H	3.92710200	5.70045100	-2.01291200
	C	3.07989900	3.88091700	-2.87328000
	H	4.00549300	3.64211800	-3.44115500
	H	2.41777800	4.43367100	-3.56746800
	C	2.39773300	2.59249200	-2.40938900
	H	1.45605600	2.84843000	-1.89449800
	H	2.15775400	1.94036500	-3.26996200

**3***1,2*	B	-1.51829900	1.09067500	0.01599800
	B	-0.69839800	-0.42149500	-0.21227200
	B	0.73191400	-0.40938200	0.71625100
	B	1.64029900	0.89655100	0.03106500
	N	-2.81047800	1.27996800	0.52167800
	N	-1.01180600	-1.24516900	-1.32004800
	N	1.16480000	-0.90455300	1.96055200
	N	2.76889300	0.86450900	-0.79096300
	C	-0.50739700	2.27654600	-0.36347200
	C	0.82534700	2.22329200	0.41030200
	O	-0.59701700	3.04710000	-1.30912500
	O	1.02653400	3.03608700	1.29973200
	C	-3.40674400	2.60284400	0.77191400
	H	-4.38402500	2.43101100	1.25464300
	C	-2.58691100	3.43165800	1.76847500
	H	-1.56173100	3.59173300	1.39053900
	H	-2.48457800	2.85884400	2.70718900
	C	-3.23884900	4.79027100	2.03248000
	H	-2.60237900	5.38209100	2.71254800
	H	-4.20522400	4.64150000	2.55306200
	C	-3.48402900	5.55308700	0.72871700
	H	-2.51132500	5.77646200	0.25161000
	H	-3.96907100	6.52265500	0.93845900
	C	-4.33811900	4.72863400	-0.23708800
	H	-4.49000700	5.27845700	-1.18210600
	H	-5.34288800	4.57845700	0.20513000
	C	-3.69470500	3.37098400	-0.52634000
	H	-4.35509700	2.75981400	-1.16656300
	H	-2.75281500	3.51113600	-1.08095900
	C	-3.68436000	0.12929700	0.76817900
	H	-3.07376200	-0.76143700	0.54224000
	C	-4.14531100	-0.00077900	2.22622400
	H	-4.80614600	0.84416500	2.49341700

H	-3.27320200	0.05507800	2.89668900
C	-4.91131300	-1.31002600	2.43001600
H	-5.24322800	-1.39639900	3.47935300
H	-4.22931600	-2.16181700	2.24428300
C	-6.10833100	-1.40992600	1.48149500
H	-6.84354000	-0.62477400	1.74217800
H	-6.62109900	-2.37884400	1.61159200
C	-5.67634900	-1.22858300	0.02462400
H	-6.55444500	-1.24875400	-0.64394600
H	-5.03468800	-2.08071800	-0.27208400
C	-4.89712700	0.07393000	-0.17132100
H	-4.55909500	0.16696700	-1.21603200
H	-5.56555100	0.93303100	0.02601000
C	-1.86705700	-0.68722200	-2.38176300
H	-2.43099600	0.11867400	-1.88488300
C	-1.05422200	0.01863000	-3.48142000
H	-0.47455100	-0.71638300	-4.06593700
H	-0.32986000	0.70012300	-3.00240300
C	-1.97162200	0.79993400	-4.42229600
H	-1.37579400	1.28341400	-5.21549800
H	-2.45855700	1.61533200	-3.85459700
C	-3.03750600	-0.11167300	-5.03291600
H	-2.54586900	-0.86566700	-5.67787800
H	-3.71488600	0.46755500	-5.68449100
C	-3.83971800	-0.82698700	-3.94448800
H	-4.57942200	-1.51099400	-4.39581600
H	-4.41703400	-0.07502100	-3.37399200
C	-2.93605200	-1.60753800	-2.98281000
H	-3.54362300	-2.04693000	-2.17306400
H	-2.47418100	-2.44457500	-3.53509500
C	-0.40379600	-2.57504000	-1.44380700
H	0.39996300	-2.57923400	-0.68815200
C	-1.36646600	-3.70934700	-1.04534300
H	-2.18522000	-3.78837100	-1.77876300
H	-1.83419800	-3.45824800	-0.07668500
C	-0.64540700	-5.05678700	-0.96763300
H	-1.36370600	-5.85480400	-0.71075800
H	0.10135000	-5.03013900	-0.15142600
C	0.06542100	-5.37753900	-2.28358200
H	-0.68796700	-5.51426900	-3.08291700
H	0.61643000	-6.33058100	-2.20137300
C	1.01707400	-4.24666900	-2.67700600
H	1.51317900	-4.47263600	-3.63680600
H	1.81947900	-4.17458400	-1.91734200
C	0.29183900	-2.90061300	-2.77096200
H	1.00695100	-2.09894700	-3.01524600
H	-0.43684200	-2.94693500	-3.59876700
C	3.51264200	2.07253600	-1.18597200
H	4.37783400	1.74226500	-1.78556400
C	4.09114700	2.80523800	0.03328100
H	4.73207900	2.10431500	0.59849200
H	3.27003000	3.10234100	0.70756900
C	4.88732000	4.04239400	-0.38592200
H	5.78792200	3.73032000	-0.95056200

H	5.24766000	4.57644100	0.51037700
C	4.04454300	4.97344600	-1.26069900
H	4.63858100	5.85200900	-1.56809200
H	3.19137700	5.35338100	-0.66786000
C	3.51026500	4.23667900	-2.49105900
H	4.36268700	3.93046800	-3.12897800
H	2.88511200	4.90988100	-3.10237300
C	2.69907400	3.00253600	-2.09158000
H	1.77282600	3.32599500	-1.58655400
H	2.37806400	2.43988400	-2.98604300
C	3.33832100	-0.41361300	-1.22679400
H	2.64546400	-1.19244200	-0.86143000
C	4.70937900	-0.70126100	-0.60117900
H	4.62707600	-0.64117000	0.49621900
H	5.43384900	0.07541500	-0.90941400
C	5.23774900	-2.07177900	-1.02726700
H	4.56581700	-2.85949900	-0.63406700
H	6.22955100	-2.25094400	-0.57724800
C	5.31091800	-2.18480600	-2.55135100
H	5.66851300	-3.18667600	-2.84635200
H	6.05333700	-1.45802300	-2.93334600
C	3.94866700	-1.90177200	-3.18765300
H	3.24478000	-2.69800800	-2.88752600
H	4.01921200	-1.94539700	-4.28838400
C	3.39218600	-0.54265500	-2.75598400
H	4.03283400	0.25330200	-3.17777000
H	2.37939700	-0.38127300	-3.16513000
C	2.40313700	-0.40714700	2.57465700
H	2.89532500	0.20791300	1.80440800
C	3.40470000	-1.51255600	2.93388900
H	3.60265400	-2.12736800	2.03736600
H	2.97877600	-2.18984700	3.69733400
C	4.70608500	-0.91513900	3.47641300
H	5.20638100	-0.33867500	2.67461700
H	5.40163600	-1.72363700	3.76145400
C	4.44435700	0.01216400	4.66515300
H	4.03849100	-0.58197100	5.50679400
H	5.39217200	0.45401500	5.01911300
C	3.44753100	1.11342300	4.29783200
H	3.89220200	1.76745500	3.52370800
H	3.24352800	1.75684900	5.17092000
C	2.13888400	0.53098100	3.75967700
H	1.61877400	-0.01445800	4.57039500
H	1.47110700	1.34186200	3.42310700
C	0.40144700	-1.84401200	2.79001300
H	0.90690000	-1.90858700	3.76844500
C	-1.02056100	-1.36454200	3.07714700
H	-1.56319400	-1.27670800	2.11954200
H	-0.98157100	-0.35247900	3.51638900
C	-1.75937900	-2.33998200	3.99380900
H	-2.78499700	-1.98118600	4.18491100
H	-1.25263200	-2.37547300	4.97747400
C	-1.78765100	-3.74548000	3.39018900
H	-2.37310900	-3.72252900	2.45020600

	H	-2.30495200	-4.44685300	4.06786000
	C	-0.37262800	-4.24351200	3.08653700
	H	-0.40927700	-5.23367000	2.59901800
	H	0.17577700	-4.38123300	4.03830600
	C	0.38745000	-3.25569400	2.20057500
	H	1.42354900	-3.59640000	2.03002900
	H	-0.09980600	-3.20051900	1.21532900

**3***1,3*	B	-1.88923200	-0.30218800	0.67138000
	C	-1.21083800	1.16956900	0.74053600
	O	-1.51915700	2.00563700	1.59886700
	N	-3.24138900	-0.59813800	0.47143600
	B	0.13804400	1.40309200	-0.10069200
	C	-0.76310600	-1.45333000	0.90925800
	O	-0.92735300	-2.23050100	1.85442000
	N	0.25033000	2.44597600	-1.03370900
	B	1.22159700	0.21324000	0.51005700
	N	2.04316000	0.55388200	1.60682200
	B	0.64892200	-1.34957600	0.10190700
	N	1.13104200	-2.31033100	-0.80464600
	C	-3.75260100	-1.97807300	0.52991700
	H	-2.88421800	-2.62912200	0.69580500
	C	-4.42196700	-2.43041700	-0.77677800
	H	-5.28075500	-1.77803200	-1.01768700
	H	-3.70312100	-2.32639100	-1.60878700
	C	-4.92301400	-3.87395800	-0.67747700
	H	-4.05995500	-4.55468800	-0.57610300
	H	-5.43762000	-4.15581900	-1.61267900
	C	-5.84395100	-4.07323900	0.52713700
	H	-6.76186800	-3.46851400	0.39400800
	H	-6.16557200	-5.12747600	0.58956500
	C	-5.14333000	-3.65071300	1.81955800
	H	-4.27212800	-4.31023900	1.99539500
	H	-5.81580600	-3.78014600	2.68518500
	C	-4.66813400	-2.19875500	1.74236600
	H	-4.11244200	-1.92104800	2.65362000
	H	-5.55247000	-1.53702800	1.68171300
	C	-4.27857200	0.42343300	0.24436200
	H	-5.21880700	-0.11849200	0.05355300
	C	-4.01767300	1.24400100	-1.02214400
	H	-3.88356600	0.55661300	-1.87591100
	H	-3.07498700	1.80646000	-0.91208800
	C	-5.16134300	2.22408800	-1.28451300
	H	-6.08887500	1.65634500	-1.49286100
	H	-4.95159400	2.81852200	-2.19030300
	C	-5.39009100	3.13397800	-0.07596600
	H	-4.48619500	3.75205000	0.08990700
	H	-6.22249200	3.83213400	-0.27311200
	C	-5.67092300	2.30992100	1.18322200
	H	-6.62770900	1.76738200	1.05012200
	H	-5.80660300	2.97441800	2.05389900

C	-4.54605500	1.31131200	1.46713400
H	-3.62125300	1.84026700	1.74486700
H	-4.81124000	0.66906200	2.32439600
C	-0.78909200	3.47346300	-1.16571100
H	-1.60318300	3.18069200	-0.48867500
C	-1.38893300	3.57022800	-2.57398500
H	-0.60709600	3.83230600	-3.31103000
H	-1.78413500	2.58096300	-2.86586200
C	-2.49545300	4.62707900	-2.62744000
H	-2.89519700	4.70440400	-3.65353600
H	-3.33438800	4.30346400	-1.98508500
C	-2.00148300	5.98886800	-2.13621900
H	-2.82430400	6.72442000	-2.16182500
H	-1.21790900	6.36515000	-2.82210800
C	-1.42725500	5.88149200	-0.72221400
H	-1.05200900	6.86187100	-0.38088900
H	-2.23442600	5.59154100	-0.02271900
C	-0.30845400	4.84027700	-0.65515800
H	0.04817400	4.71996300	0.38270100
H	0.54979700	5.19569000	-1.25635300
C	1.44396200	2.65967300	-1.86076300
H	1.27066700	3.57047400	-2.45779900
C	1.66099100	1.52121100	-2.85877200
H	1.83807900	0.59181200	-2.28864600
H	0.73901800	1.36614600	-3.44576200
C	2.85129400	1.80513200	-3.77593300
H	2.62530900	2.69111400	-4.40017100
H	3.00266400	0.96426700	-4.47426900
C	4.12409600	2.07263700	-2.96910700
H	4.40783600	1.15335900	-2.42051300
H	4.96472100	2.31084800	-3.64400400
C	3.91098600	3.20157500	-1.95848000
H	3.73991000	4.15129800	-2.50112400
H	4.81954400	3.34816200	-1.34847900
C	2.71657700	2.90975500	-1.04950500
H	2.54824600	3.74331100	-0.34614500
H	2.93090200	2.01242800	-0.44507700
C	1.98820500	1.90690400	2.18253200
H	1.43268400	2.51894500	1.45457700
C	3.34422600	2.59890900	2.37934900
H	3.94219800	2.06829500	3.14365400
H	3.92677600	2.57322600	1.44373800
C	3.13501500	4.04809100	2.82961800
H	4.11159000	4.54052500	2.98079900
H	2.62264900	4.60560900	2.02161600
C	2.29434400	4.12684700	4.10631400
H	2.11819100	5.18158800	4.38177900
H	2.86444600	3.67369200	4.94032000
C	0.96446500	3.38677200	3.94679600
H	0.40027400	3.40186300	4.89525700
H	0.33392600	3.90267800	3.19862000
C	1.17582500	1.94427000	3.48556800
H	0.20311900	1.45595000	3.31951100
H	1.70361500	1.37615900	4.27558200

C	2.89301500	-0.38537800	2.35223200
H	3.14424400	0.09833400	3.31161100
C	4.22069200	-0.64494400	1.63087300
H	4.71498900	0.31513100	1.40519400
H	4.00005300	-1.12186800	0.66072300
C	5.14876500	-1.53621800	2.46038600
H	6.06798500	-1.75842600	1.89007000
H	5.46735900	-0.97814500	3.36199500
C	4.46507200	-2.83544500	2.89572100
H	5.13463700	-3.41169800	3.55814800
H	4.28407800	-3.47020200	2.00802800
C	3.13035100	-2.56245800	3.59335500
H	2.62642400	-3.51223600	3.84190700
H	3.31898100	-2.04538900	4.55415000
C	2.21113700	-1.70251400	2.72375100
H	1.95976800	-2.27026500	1.81138500
H	1.25833100	-1.49875800	3.23678600
C	0.48601700	-3.60710600	-1.05506000
H	1.06495900	-4.11009700	-1.84770000
C	-0.93119100	-3.46964900	-1.61478700
H	-0.90268500	-2.83945500	-2.52110400
H	-1.56895200	-2.94805000	-0.88315800
C	-1.53685400	-4.84024400	-1.92109700
H	-2.56933700	-4.72220300	-2.29321100
H	-0.96402500	-5.31952900	-2.73850000
C	-1.50593800	-5.74820700	-0.68931100
H	-1.93588500	-6.73608500	-0.93104900
H	-2.14009000	-5.30886300	0.10421200
C	-0.08252200	-5.89824000	-0.14932600
H	0.53800500	-6.42224100	-0.90268800
H	-0.08009900	-6.53112200	0.75486300
C	0.53469100	-4.53502200	0.16731600
H	0.00197500	-4.05713800	1.00634700
H	1.58496300	-4.65401700	0.48592300
C	2.41149700	-2.12424000	-1.49484000
H	2.77819400	-1.13173000	-1.18049700
C	2.28276000	-2.09043900	-3.02533100
H	1.90268600	-3.06098400	-3.39425900
H	1.53932800	-1.32896000	-3.31536400
C	3.63519200	-1.80656800	-3.68444200
H	3.52689500	-1.80572300	-4.78303300
H	3.96930300	-0.79279500	-3.40029900
C	4.70289900	-2.81202000	-3.24964800
H	5.67475300	-2.55850500	-3.70773500
H	4.43099800	-3.81946300	-3.61898500
C	4.82496700	-2.85242600	-1.72517700
H	5.56797800	-3.60771500	-1.41551300
H	5.19835800	-1.87542500	-1.36132200
C	3.47381700	-3.15089900	-1.07459300
H	3.56119000	-3.15574900	0.02341400
H	3.15315400	-4.16664700	-1.37094300

**3**$_{1,4}$	B	1.35133300	0.36472000	-0.31369400
	N	2.67447700	0.56417700	0.13166500
	C	0.88876000	-0.97704100	-1.09508700
	O	1.23768900	-1.28433800	-2.23744200
	B	-1.56508200	-0.59581400	0.03300500
	N	-2.81044400	-0.55064900	-0.60380100
	B	-0.26020600	-1.68463900	-0.20501800
	N	-0.14424000	-2.84022400	0.58371200
	C	3.79085500	-0.18237800	-0.46476500
	H	3.32421400	-1.02973300	-0.99016100
	C	4.80629800	-0.78886600	0.51239400
	H	5.35588200	0.00246500	1.04884500
	H	4.28176400	-1.38573600	1.27574700
	C	5.81561600	-1.66122300	-0.23971900
	H	5.28700700	-2.52054700	-0.69208700
	H	6.54931000	-2.08102000	0.47043200
	C	6.52482500	-0.87891000	-1.34623900
	H	7.13203900	-0.07231800	-0.89175600
	H	7.22664200	-1.53623500	-1.88863800
	C	5.51246200	-0.26517900	-2.31505000
	H	4.97407400	-1.07501500	-2.84283600
	H	6.02907400	0.32764300	-3.08982200
	C	4.49695300	0.61046000	-1.57858100
	H	3.73150000	0.97744200	-2.28339300
	H	5.01113900	1.49290800	-1.16130400
	C	2.87791400	1.53929800	1.21887400
	H	2.00053200	2.20427300	1.15693100
	C	4.08803500	2.47939100	1.13408700
	H	4.12567300	2.97001000	0.14949400
	H	5.03407100	1.92519800	1.25421600
	C	3.99477100	3.54433800	2.23227600
	H	3.10544800	4.17725400	2.04588200
	H	4.87195100	4.21230200	2.17806000
	C	3.88606500	2.91988500	3.62473700
	H	4.82426100	2.37759000	3.85139700
	H	3.78511700	3.70912800	4.39020300
	C	2.71018100	1.94458200	3.70554000
	H	1.75965000	2.50125300	3.59639800
	H	2.67656800	1.46143400	4.69722400
	C	2.78971000	0.88329300	2.60708000
	H	3.66661200	0.23437600	2.77935900
	H	1.89415700	0.23802000	2.63635200
	C	-3.84446300	0.41335100	-0.20706400
	H	-3.34641500	1.15376800	0.44099400
	C	-4.93005500	-0.25971000	0.64539100
	H	-5.41781000	-1.05983100	0.05559500
	H	-4.44553900	-0.74429700	1.51087300
	C	-5.98831600	0.74186300	1.11052800
	H	-6.77030400	0.22281600	1.69144800
	H	-5.51827800	1.47216500	1.79672900
	C	-6.60501700	1.48932800	-0.07306300
	H	-7.34496400	2.22774600	0.28174700
	H	-7.15438200	0.77180000	-0.71269900
	C	-5.52254100	2.18302100	-0.90170600

H	-5.96939300	2.69780500	-1.76996900
H	-5.04919700	2.96551100	-0.28222700
C	-4.46010700	1.18968000	-1.38140700
H	-3.65863400	1.70605000	-1.93842600
H	-4.93862500	0.49541900	-2.09429100
C	-3.19694400	-1.51564400	-1.64704100
H	-4.24591900	-1.30885100	-1.91375800
C	-3.16503700	-2.97250400	-1.18117900
H	-3.78651800	-3.08381900	-0.27494600
H	-2.13292300	-3.23549700	-0.90384400
C	-3.63099700	-3.92162700	-2.28602200
H	-3.57254600	-4.96541900	-1.93005700
H	-4.69596800	-3.72826500	-2.52011700
C	-2.79131200	-3.74148100	-3.55291900
H	-3.15084300	-4.41393000	-4.35140000
H	-1.74516500	-4.03117500	-3.33707400
C	-2.81775900	-2.28725200	-4.02922000
H	-2.17371500	-2.16199600	-4.91646600
H	-3.84685300	-2.02749200	-4.34577600
C	-2.36800900	-1.33029000	-2.92357800
H	-1.30009600	-1.50858200	-2.69972900
H	-2.44810200	-0.28121300	-3.25815500
C	0.95018000	-3.80886200	0.48079200
H	0.73789000	-4.62281400	1.19339800
C	2.30774100	-3.23124900	0.88115500
H	2.24556100	-2.81064300	1.89973300
H	2.54755200	-2.39699500	0.20502100
C	3.40858400	-4.28807300	0.78562500
H	4.38261600	-3.84778000	1.06078700
H	3.21072800	-5.09246700	1.52038400
C	3.46949700	-4.89158900	-0.61928100
H	4.25571000	-5.66502500	-0.67149700
H	3.74999600	-4.10141100	-1.34190100
C	2.11597400	-5.47800700	-1.02722000
H	1.88211400	-6.33843500	-0.37047800
H	2.16303700	-5.87229300	-2.05702400
C	1.00046500	-4.43693300	-0.91838200
H	1.15384600	-3.63565600	-1.66441100
H	0.01989600	-4.89597600	-1.13844700
C	-1.12508000	-3.07252700	1.65247100
H	-1.95183000	-2.37049700	1.45016100
C	-0.57154800	-2.70288200	3.03429800
H	0.23937600	-3.40637200	3.30760800
H	-0.13557000	-1.69119600	2.98776100
C	-1.68076400	-2.74623600	4.08673300
H	-1.26705700	-2.51617200	5.08375300
H	-2.40575700	-1.94358300	3.85572400
C	-2.39221200	-4.10172800	4.10645000
H	-3.23288300	-4.08259400	4.82202000
H	-1.68819600	-4.87610200	4.46787900
C	-2.89160800	-4.49926000	2.71445400
H	-3.34903100	-5.50353400	2.74388100
H	-3.68726500	-3.79892800	2.39480900
C	-1.75584700	-4.47113200	1.68701500

H	-2.12939200	-4.75009500	0.68810500
H	-1.00559100	-5.23051100	1.97327900
B	0.05141200	1.37877900	0.20321100
N	-0.24402800	2.69565200	-0.19864700
C	-1.03331600	0.54392600	1.04618500
O	-1.33272100	0.60787100	2.23908900
C	0.34564500	3.33264900	-1.38397200
C	1.84646100	3.61188900	-1.27535800
C	0.03571500	2.53292400	-2.65638700
H	-0.14397400	4.31383300	-1.50198500
C	2.37193400	4.32880100	-2.52021800
H	2.37636800	2.65359800	-1.16839500
H	2.04987100	4.20512000	-0.36692000
C	0.56592400	3.23587300	-3.90697600
H	0.49457400	1.52875300	-2.57871800
H	-1.05517000	2.37558000	-2.72633900
C	2.06306100	3.53030800	-3.78880500
H	3.45994500	4.49219600	-2.42490000
H	1.90836600	5.33165200	-2.59566900
H	0.36501500	2.61755800	-4.79871700
H	0.01844500	4.18745100	-4.05201100
H	2.41905000	4.07376100	-4.68142300
H	2.61722300	2.57304200	-3.75675800
C	-1.15584700	3.52270800	0.59801100
C	-0.41258900	4.65816600	1.31781600
C	-2.36130700	4.07125800	-0.17171100
H	-1.54849400	2.86558200	1.38986900
C	-1.35295400	5.42385600	2.25199100
H	0.01676900	5.35705200	0.57427000
H	0.43288100	4.23148200	1.88627800
C	-3.30948300	4.81638700	0.77065500
H	-2.03445100	4.76636400	-0.96655600
H	-2.87839400	3.23825800	-0.67176800
C	-2.58428100	5.94551600	1.50721300
H	-0.81306900	6.25825500	2.73256000
H	-1.67884000	4.74828600	3.06527700
H	-4.16832900	5.21710700	0.20451700
H	-3.72289500	4.10333100	1.50975100
H	-3.27168100	6.44775800	2.20992100
H	-2.26823100	6.71047200	0.77205000

**TrippB(CO)₂**	O	1.97823600	-0.11295800	-2.53079700
	C	-2.50290600	0.13049400	0.75044300
	C	0.00000200	0.00122500	0.76602300
	C	-1.20050000	0.06453100	2.88593000
	H	-2.15313100	0.11415600	3.42219300
	C	-4.34216300	-0.97727300	-0.35483700
	H	-4.84928700	-1.90150500	-0.65090000
	C	4.22393600	-1.41560500	-0.34987700
	H	4.64393400	-2.38312100	-0.64538800
	C	2.50285300	-0.12917000	0.75056600

C	1.20051700	-0.05992000	2.88598800
H	2.15314300	-0.10916000	3.42229700
O	-1.97814800	0.11283200	-2.53092800
C	-1.16165500	0.06648000	-1.72321500
C	-1.20868500	0.06421600	1.48883700
C	2.59906200	2.42991200	0.74623500
H	1.62190500	2.27249200	1.22843500
C	3.03611600	-1.38110700	0.38499200
C	1.20868400	-0.06120700	1.48889600
C	-4.88915600	0.25033800	-0.73669500
C	-3.03718600	1.38160100	0.38354400
C	-2.34403500	2.68495500	0.75739400
H	-1.38918400	2.42956900	1.24238700
C	-3.15914200	-1.06253500	0.37854700
C	-2.00344300	3.53315000	-0.47380400
H	-1.39029100	2.96038700	-1.19073500
H	-1.42855500	4.42781700	-0.17586600
H	-2.91062800	3.87929400	-1.00032100
C	4.88903100	-0.25252500	-0.73639500
C	-6.15489200	0.31560900	-1.57284000
H	-6.39605800	1.38426900	-1.71352100
C	1.16173800	-0.06588400	-1.72312900
C	-2.59716700	-2.42873700	0.74884700
H	-1.62097000	-2.27003900	1.23255400
C	4.34306500	0.97593100	-0.35578500
H	4.85097900	1.89943400	-0.65274900
C	6.15472200	-0.31970200	-1.57245800
H	6.39498900	-1.38870600	-1.71206500
C	0.00001600	0.00277000	3.58904500
H	0.00002200	0.00340200	4.68267200
C	-4.22506000	1.41436600	-0.35131600
H	-4.64588800	2.38122800	-0.64777600
C	3.16004900	1.06292100	0.37740100
C	-7.34724400	-0.33490400	-0.86154100
H	-7.18453600	-1.41734000	-0.71279100
H	-8.26913700	-0.21461000	-1.45760600
H	-7.51579000	0.12000100	0.12948300
C	2.34889200	3.30529900	-0.48760100
H	3.28785800	3.55968100	-1.01080900
H	1.86377100	4.25282700	-0.19347800
H	1.68533600	2.79438300	-1.20638800
C	-3.17230700	3.47495900	1.77792800
H	-4.14907800	3.77487800	1.35738700
H	-2.63998400	4.39245600	2.08568200
H	-3.36496000	2.87093900	2.68122300
B	0.00002800	0.00065200	-0.80864400
C	-2.34399900	-3.30424900	-0.48429800
H	-3.28179100	-3.55931100	-1.00928000
H	-1.85883500	-4.25139800	-0.18901600
H	-1.67932600	-2.79308200	-1.20187000
C	-3.49807600	-3.13468500	1.76943200
H	-3.62835900	-2.51636800	2.67419000
H	-3.05931700	-4.10126800	2.07448800
H	-4.50060500	-3.33482800	1.35004100

C	-5.92960400	-0.29217600	-2.96338400
H	-5.07685200	0.18875400	-3.47148800
H	-6.82736400	-0.16879900	-3.59484400
H	-5.71176800	-1.37303700	-2.89432800
C	1.99917200	-3.53186900	-0.47033700
H	2.90546100	-3.87879400	-0.99788800
H	1.38550600	-2.95887400	-1.18664100
H	1.42399500	-4.42602400	-0.17141800
C	3.17036500	-3.47404200	1.78020000
H	4.14630500	-3.77542700	1.35878300
H	2.63723100	-4.39071600	2.08899800
H	3.36474700	-2.86980100	2.68297700
C	7.34761500	0.33051600	-0.86179900
H	7.18582400	1.41323800	-0.71413800
H	8.26941300	0.20884400	-1.45773200
H	7.51576700	-0.12353700	0.12968400
C	5.92996200	0.28688100	-2.96361200
H	5.07681500	-0.19384200	-3.47124900
H	6.82762700	0.16212400	-3.59493600
H	5.71302400	1.36799200	-2.89563800
C	2.34194200	-2.68354600	0.76017400
H	1.38786700	-2.42692500	1.24603300
C	3.49919300	3.13513400	1.76801100
H	4.50269900	3.33363600	1.35017100
H	3.62709500	2.51708000	2.67328600
H	3.06126700	4.10247600	2.07186100

**[cAAC(CO)B]$_2$**	O	-1.28022700	-1.96749100	1.62391700
	N	-3.26567900	0.72522900	-0.53399500
	C	-1.19574300	-1.10104200	0.85948200
	B	-0.86500300	-0.04611100	-0.10470300
	O	1.28017400	1.96736000	1.62410500
	N	3.26565700	-0.72515900	-0.53407900
	C	1.19570400	1.10097400	0.85960000
	B	0.86497600	0.04614900	-0.10470600
	C	-1.92200400	0.81274000	-0.72772900
	C	-1.66105900	1.99897700	-1.65525600
	C	-3.05206200	2.28144700	-2.25995500
	H	-3.21627100	3.35420300	-2.45347600
	H	-3.13880000	1.75769500	-3.22720400
	C	-4.09302900	1.70918000	-1.28388800
	C	4.09299500	-1.70904100	-1.28405300
	C	3.05203100	-2.28122000	-2.26017500
	H	3.21623200	-3.35396200	-2.45377800
	H	3.13877100	-1.75739500	-3.22738400
	C	1.66103500	-1.99879900	-1.65545300
	C	1.92197800	-0.81264400	-0.72781800
	C	-4.18116000	-1.50728000	-0.09152900
	C	-3.82072400	-0.22344800	0.37886400
	C	-4.73209800	-2.41677500	0.81523800
	H	-5.02237400	-3.41299700	0.46889500

C	-4.89462100	-2.08939000	2.15515400
H	-5.32427000	-2.81672900	2.84985900
C	-4.47616400	-0.84771700	2.61656800
H	-4.56519400	-0.61602100	3.68186400
C	-3.91993900	0.09997300	1.75333300
C	-3.92649400	-1.97066100	-1.51987300
H	-3.51010500	-1.12020200	-2.07882200
C	-2.87112500	-3.08474200	-1.56286200
H	-2.65914900	-3.36772200	-2.60924700
H	-3.21539100	-3.98906100	-1.03034100
H	-1.92780300	-2.75344600	-1.10406100
C	-5.21000900	-2.43548400	-2.22156300
H	-5.01092200	-2.64066500	-3.28827200
H	-6.01283100	-1.68330600	-2.16079600
H	-5.59394000	-3.36903400	-1.77350600
C	-3.38162500	1.39414000	2.35090500
H	-3.00031700	2.01076400	1.52685800
C	-2.19450800	1.13527600	3.29129400
H	-1.80713900	2.09334200	3.68004100
H	-1.36874100	0.62950100	2.76873300
H	-2.49182900	0.51428900	4.15487000
C	-4.46565900	2.19043600	3.09071400
H	-4.07480900	3.17809000	3.39237100
H	-4.78696600	1.66921800	4.00989300
H	-5.36243200	2.35280400	2.47114600
C	-5.25188100	1.04227300	-2.02946500
H	-5.83078400	1.81322600	-2.56609300
H	-5.93478000	0.52956200	-1.33064100
H	-4.89551600	0.31409900	-2.77289000
C	-4.69481400	2.78318900	-0.36754300
H	-5.28050900	3.49571100	-0.97291700
H	-3.92904300	3.35476800	0.17660900
H	-5.37673200	2.32502400	0.36646500
C	-0.64771900	1.68431400	-2.75861800
H	-0.54307300	2.55423000	-3.43276800
H	-0.97557800	0.81851400	-3.35859400
H	0.33876700	1.44477000	-2.33073000
C	-1.15322000	3.19158900	-0.82373900
H	-1.04546700	4.08280000	-1.46813700
H	-0.17348200	2.97020800	-0.37523600
H	-1.83960500	3.44486400	0.00041000
C	3.82072700	0.22340600	0.37888200
C	4.18122100	1.50727400	-0.09136800
C	4.73216100	2.41666000	0.81550800
H	5.02247300	3.41291000	0.46927600
C	4.89463400	2.08913600	2.15539600
H	5.32427000	2.81640200	2.85019300
C	4.47614100	0.84742300	2.61666900
H	4.56512900	0.61561700	3.68194600
C	3.91992900	-0.10017100	1.75331700
C	3.92660800	1.97082200	-1.51966300
H	3.51021900	1.12044100	-2.07872900
C	2.87127600	3.08494000	-1.56254100
H	2.65931500	3.36803400	-2.60889200

H	3.21556900	3.98919100	-1.02993600
H	1.92794700	2.75363400	-1.10377800
C	5.21015100	2.43570400	-2.22124600
H	5.01107100	2.64117700	-3.28789900
H	6.01290600	1.68343700	-2.16067900
H	5.59417200	3.36910000	-1.77295300
C	3.38162900	-1.39441000	2.35073700
H	3.00045200	-2.01099800	1.52660300
C	2.19439100	-1.13568800	3.29099700
H	1.80710300	-2.09380300	3.67969500
H	1.36860800	-0.63000600	2.76835000
H	2.49155900	-0.51466700	4.15460300
C	4.46562700	-2.19070900	3.09059100
H	4.07470700	-3.17831500	3.39228700
H	4.78698800	-1.66951000	4.00974500
H	5.36235800	-2.35318500	2.47102300
C	5.25184700	-1.04205100	-2.02955900
H	5.83072200	-1.81294200	-2.56631200
H	5.93477600	-0.52945300	-1.33067600
H	4.89547500	-0.31376400	-2.77287200
C	4.69473600	-2.78312800	-0.36776900
H	5.28044800	-3.49559500	-0.97318000
H	3.92894400	-3.35475600	0.17629200
H	5.37662300	-2.32503500	0.36630400
C	0.64771000	-1.68404800	-2.75880100
H	0.54298600	-2.55394800	-3.43296000
H	0.97563700	-0.81826800	-3.35877000
H	-0.33875200	-1.44442000	-2.33090100
C	1.15321800	-3.19149200	-0.82404400
H	1.04549400	-4.08265200	-1.46851700
H	0.17346600	-2.97017000	-0.37554000
H	1.83958900	-3.44481700	0.00009900

**$C_2(IDip{\cdot}BC(O)O)_2$**	O	0.22266900	-1.40480700	1.03714600
	C	-0.44377700	-0.48990400	0.28387800
	B	-1.80536900	-0.15147600	-0.08026900
	O	2.31949700	-1.82899000	1.67036700
	C	1.59824700	-1.12908600	1.00012800
	C	-3.13044500	-0.77272500	0.40219200
	N	-4.26652300	-0.12021600	0.73706500
	C	-5.22303400	-1.01751500	1.17111100
	H	-6.21007900	-0.69029900	1.48613800
	C	-4.66471900	-2.25200200	1.09955100
	H	-5.07215500	-3.23541700	1.31462300
	N	-3.37257100	-2.08462500	0.63916900
	C	-4.44377400	1.30191200	0.65167400
	C	-3.81812800	2.12425100	1.60283600
	C	-4.03000700	3.50105700	1.48997600
	H	-3.56186600	4.17694200	2.21110600
	C	-4.82520000	4.02638400	0.47723600
	H	-4.97853700	5.10733800	0.41095900

C	-5.40846400	3.18545200	-0.46401900
H	-6.00647300	3.61418600	-1.27291100
C	-5.21413900	1.80437800	-0.40828100
C	-2.94721500	1.58180000	2.72679500
H	-2.81153000	0.50102000	2.57114800
C	-1.54488300	2.20519000	2.71564600
H	-1.06159900	2.08966400	1.73147300
H	-0.90491700	1.70629100	3.46376700
H	-1.57389600	3.28169100	2.96274900
C	-3.63662800	1.76034900	4.08606500
H	-3.78977700	2.82874800	4.32173200
H	-3.02088000	1.32136500	4.89076600
H	-4.62425000	1.26706500	4.10146000
C	-5.79987700	0.90230800	-1.48378000
H	-5.39539100	-0.11133800	-1.33287300
C	-5.36328300	1.33989200	-2.88720000
H	-5.81604700	2.30672500	-3.17084800
H	-5.68523700	0.59169000	-3.63350400
H	-4.26735000	1.44571300	-2.92558500
C	-7.32624500	0.81114500	-1.36403700
H	-7.63541300	0.44553500	-0.36906200
H	-7.73509800	0.12234100	-2.12424300
H	-7.79521200	1.79968400	-1.51652100
C	-2.46372800	-3.14313100	0.28925100
C	-2.39391800	-3.52076700	-1.06178100
C	-1.48676800	-4.52781500	-1.40058500
H	-1.39603800	-4.84322100	-2.44409600
C	-0.68934500	-5.12494400	-0.43054500
H	0.02284300	-5.90472700	-0.71536900
C	-0.77718900	-4.72371800	0.89836600
H	-0.12042800	-5.18288900	1.63967100
C	-1.66013600	-3.71536700	1.29026200
C	-3.23928200	-2.86495500	-2.14376800
H	-3.88843300	-2.11191200	-1.66983900
C	-2.36224500	-2.11996600	-3.15776700
H	-1.72349300	-2.81974000	-3.72605200
H	-1.70801100	-1.39448800	-2.64611100
H	-2.99145600	-1.56971600	-3.87908900
C	-4.16424200	-3.87983200	-2.82669300
H	-4.81141600	-3.37337600	-3.56430900
H	-4.81239000	-4.38814700	-2.09172300
H	-3.58938000	-4.65564400	-3.36273400
C	-1.73990200	-3.25960200	2.74016200
H	-2.01967500	-2.19213300	2.72698300
C	-2.82830500	-4.03371500	3.50088600
H	-3.82660600	-3.91542100	3.04992900
H	-2.88917300	-3.68347300	4.54638700
H	-2.59250500	-5.11285400	3.51680300
C	-0.40724000	-3.37111500	3.48848800
H	-0.48419900	-2.84349000	4.45526400
H	0.42168100	-2.92702000	2.91523900
H	-0.15885900	-4.42362500	3.71610500
O	-0.22260200	1.40499900	-1.03690900
C	0.44383200	0.49011600	-0.28360600

B	1.80542100	0.15162800	0.08044900
O	-2.31939800	1.82911000	-1.67027300
C	-1.59817900	1.12922000	-0.99998600
C	3.13050600	0.77281200	-0.40209200
N	4.26631600	0.12016400	-0.73757300
C	5.22292300	1.01741100	-1.17150700
H	6.20978700	0.69009600	-1.48699600
C	4.66493400	2.25200900	-1.09924000
H	5.07256400	3.23541400	-1.31399000
N	3.37287700	2.08474800	-0.63856000
C	4.44303200	-1.30208000	-0.65302300
C	3.81677600	-2.12359800	-1.60449800
C	4.02796200	-3.50057000	-1.49240100
H	3.55932400	-4.17583200	-2.21379000
C	4.82311100	-4.02683200	-0.48011100
H	4.97590300	-5.10789800	-0.41442800
C	5.40704900	-3.18669000	0.46142900
H	6.00505800	-3.61615300	1.26993200
C	5.21341800	-1.80548600	0.40647000
C	2.94606100	-1.58008700	-2.72809700
H	2.81087900	-0.49933700	-2.57182800
C	1.54342800	-2.20278500	-2.71735500
H	1.06008700	-2.08745600	-1.73318100
H	0.90381900	-1.70320800	-3.46532600
H	1.57190000	-3.27918200	-2.96496500
C	3.63539700	-1.75815800	-4.08747200
H	3.78802800	-2.82649000	-4.32377300
H	3.01986900	-1.31839200	-4.89191300
H	4.62326000	-1.26534800	-4.10256500
C	5.80003600	-0.90425400	1.48218900
H	5.39570800	0.10958600	1.33217500
C	5.36427500	-1.34258100	2.88562800
H	5.81702300	-2.30966800	3.16842500
H	5.68688900	-0.59488700	3.63215400
H	4.26834500	-1.44815800	2.92466800
C	7.32635500	-0.81342700	1.36150900
H	7.63499100	-0.44727600	0.36657200
H	7.73589600	-0.12522200	2.12188800
H	7.79513100	-1.80219800	1.51305700
C	2.46432900	3.14324000	-0.28782500
C	2.39505500	3.52021100	1.06342400
C	1.48804100	4.52709300	1.40307900
H	1.39770700	4.84197900	2.44678200
C	0.69023400	5.12469400	0.43364300
H	-0.02185800	5.90432100	0.71913600
C	0.77760400	4.72417100	-0.89551300
H	0.12059100	5.18373900	-1.63635000
C	1.66042300	3.71603900	-1.28826500
C	3.24088800	2.86389300	2.14474700
H	3.88998900	2.11121300	1.67016900
C	2.36435100	2.11820300	3.15866400
H	1.72556100	2.81754600	3.72743400
H	1.71020600	1.39273100	2.64689000
H	2.99393300	1.56782100	3.87956300

C	4.16595000	3.87852500	2.82790400
H	4.81350400	3.37173800	3.56495900
H	4.81371500	4.38738900	2.09297900
H	3.59117500	4.65391900	3.36464300
C	1.73982400	3.26117900	-2.73847000
H	2.01947600	2.19366800	-2.72602700
C	2.82818100	4.03566900	-3.49888500
H	3.82654000	3.91703000	-3.04815000
H	2.88886500	3.68606300	-4.54460900
H	2.59246200	5.11483700	-3.51410400
C	0.40705500	3.37334700	-3.48649900
H	0.48386600	2.84652300	-4.45372400
H	-0.42180000	2.92877900	-2.91352100
H	0.15867600	4.42605500	-3.71320800

$(IMes\cdot BBr)_2C(O)O$

C	-2.10611600	2.98962700	0.07063200
C	-2.50182400	3.48885500	1.32032600
C	-3.81158400	3.23891400	1.72807300
H	-4.14010700	3.61099700	2.70419700
C	-4.71319900	2.53908600	0.91975400
C	-4.27981300	2.08636900	-0.32573700
H	-4.97547700	1.54539500	-0.97406600
C	-2.97346700	2.29344500	-0.77723000
C	-1.55340300	4.27925900	2.18278400
H	-2.02942100	4.54672700	3.13915600
H	-1.24073500	5.21596000	1.68749500
H	-0.64497500	3.69301900	2.40388100
C	-6.11935800	2.27969700	1.39834300
H	-6.73514200	1.81676200	0.61006500
H	-6.61522300	3.21463800	1.71252500
H	-6.12037000	1.60176900	2.27102500
C	-2.52567300	1.75961000	-2.10781000
H	-3.39059900	1.53078300	-2.75021400
H	-1.95044200	0.82899900	-1.95629000
H	-1.87403000	2.47135600	-2.64309700
C	2.65119900	2.62756400	-1.08163700
C	2.90207500	1.98018000	-2.29667200
C	4.17584300	1.45020100	-2.49154900
H	4.38830800	0.91934700	-3.42512300
C	5.16566300	1.53924100	-1.51107300
C	4.87743600	2.22188700	-0.32842700
H	5.65023900	2.31466900	0.44195100
C	3.62182000	2.78166900	-0.08834100
C	1.81340700	1.78645200	-3.31677900
H	2.23445200	1.46260300	-4.28142600
H	1.22888500	2.70677000	-3.48870200
H	1.11116200	0.99821100	-2.98567000
C	6.49710800	0.86163800	-1.70614100
H	7.28282300	1.31892700	-1.08216300
H	6.82389400	0.90210100	-2.75876900
H	6.42194800	-0.20419700	-1.42277000

C	3.31881700	3.49638900	1.20094900
H	4.24462700	3.69271900	1.76460500
H	2.65177400	2.88509600	1.83480400
H	2.81324200	4.46226000	1.02629400
C	1.92119400	-2.99894500	1.10279200
C	2.31399900	-2.43569900	2.32519700
C	3.65836200	-2.10514400	2.47713600
H	3.98272400	-1.63291900	3.40994800
C	4.59130500	-2.33754300	1.46175700
C	4.16138700	-2.95217900	0.28750900
H	4.88104500	-3.14227800	-0.51542100
C	2.82192600	-3.28193300	0.07470500
C	1.31841200	-2.19589000	3.42986000
H	1.75921000	-1.56955200	4.22024700
H	0.99289200	-3.14732700	3.88923800
H	0.42228400	-1.66969200	3.06141700
C	6.01649800	-1.87279000	1.61126100
H	6.10082800	-0.81841800	1.28972700
H	6.70583900	-2.46775800	0.98938300
H	6.35735200	-1.93011200	2.65859100
C	2.36809600	-3.83815700	-1.24532500
H	3.19579900	-4.35347900	-1.75870500
H	2.01901400	-3.01448400	-1.89359700
H	1.53006900	-4.54698200	-1.13723600
C	-2.83948200	-2.49521900	0.02863600
C	-3.39707000	-3.05454100	-1.13010700
C	-4.63993700	-2.57573700	-1.54408300
H	-5.08677000	-2.98718400	-2.45504700
C	-5.32774800	-1.59637100	-0.82157400
C	-4.75005600	-1.10210800	0.34772500
H	-5.28751600	-0.35565600	0.93854200
C	-3.49621400	-1.52555300	0.79495700
C	-2.69373600	-4.15086400	-1.88658400
H	-3.21305000	-4.36314900	-2.83418000
H	-2.66446200	-5.08790600	-1.30148300
H	-1.65599400	-3.86377100	-2.12474600
C	-6.65183500	-1.06690600	-1.31038800
H	-7.23859100	-0.62604600	-0.48759100
H	-7.25837000	-1.86005400	-1.77893400
H	-6.49976500	-0.27894800	-2.07118100
C	-2.88254400	-0.93883400	2.03487700
H	-3.64025200	-0.40991000	2.63436900
H	-2.09598600	-0.21866300	1.74634300
H	-2.41303100	-1.71066800	2.66938100
N	0.51870800	-3.26042700	0.91338700
C	-0.36073400	-2.43347700	0.31044200
N	-1.57346300	-3.00115200	0.48669100
C	-1.45332400	-4.18271400	1.19113200
H	-2.32221600	-4.78835000	1.43507500
C	-0.13426500	-4.34728100	1.45728400
H	0.40585500	-5.12599600	1.98893300
N	1.32526100	3.13846700	-0.85200600
C	0.29915800	2.45051000	-0.31148700
N	-0.77049200	3.27091900	-0.38346300

C	-0.41449100	4.47302500	-0.96379400
H	-1.14124400	5.26713400	-1.11415000
C	0.90681400	4.39023600	-1.25648500
H	1.59141900	5.09239400	-1.72484800
B	-0.00722900	-1.03709900	-0.45090200
B	0.35379900	0.94123400	0.28458100
Br	0.55782800	1.20776400	2.38042000
Br	-0.28175600	-1.44598300	-2.50733200
O	-0.77935000	0.10866000	-0.02954200
C	1.40271800	-0.21473600	-0.22691600
O	2.58915500	-0.35993300	-0.42151500

acetaldehyde

C	1.16888700	-0.13361600	-0.00000300
H	1.92434400	0.66768800	-0.00011200
H	1.31269000	-0.77403600	-0.88782400
H	1.31276500	-0.77385800	0.88793300
C	-0.24220600	0.40885100	0.00001200
O	-1.22067000	-0.28714500	-0.00000300
H	-0.34451900	1.52596000	-0.00002500

acetone

C	1.28765000	-0.61318500	-0.00001500
H	1.33297700	-1.26800400	0.88847300
H	2.15099300	0.06872800	-0.00026300
H	1.33254700	-1.26817700	-0.88841000
C	-1.28764900	-0.61318600	0.00003800
H	-1.33134700	-1.27092900	0.88643900
H	-2.15099100	0.06872100	0.00340800
H	-1.33417600	-1.26524900	-0.89043400
C	0.00000000	0.18898800	0.00015700
O	-0.00000100	1.39490100	-0.00003600

benzophenone

C	0.00000000	1.10327000	-0.00000200
O	0.00000100	2.31585500	-0.00000100
C	-1.29909800	0.34933000	-0.02766100
C	-2.42894000	0.96962400	0.52157200
C	-1.43612700	-0.89950500	-0.64704700
C	-3.66780600	0.33732600	0.48415700
H	-2.31530700	1.95687700	0.97664600
C	-2.68169900	-1.52404700	-0.70131700
H	-0.56976500	-1.37934100	-1.11056300
C	-3.79558000	-0.91158100	-0.12782000
H	-4.54194200	0.82234000	0.92774500
H	-2.78260700	-2.49355300	-1.19723700
H	-4.77057400	-1.40640600	-0.16357400
C	1.29909600	0.34932700	0.02765800
C	1.43612500	-0.89951000	0.64704000
C	2.42894100	0.96962400	-0.52156900

	C	2.68169700	-1.52405000	0.70131500
	H	0.56976200	-1.37935400	1.11054600
	C	3.66780800	0.33732800	-0.48414900
	H	2.31530900	1.95687800	-0.97664100
	C	3.79558100	-0.91158100	0.12782500
	H	2.78260500	-2.49355800	1.19723100
	H	4.54194500	0.82234500	-0.92773300
	H	4.77057600	-1.40640300	0.16358400

**cinnamaldehyde**	C	3.36707700	-0.28604700	0.00000100
	O	4.35996700	0.39706800	0.00003000
	H	3.43922300	-1.40650400	-0.00002400
	C	1.99243800	0.24039800	-0.00000300
	H	1.89548600	1.33122200	0.00002300
	C	0.93762600	-0.59419500	-0.00002700
	H	1.15006300	-1.67282600	-0.00004900
	C	-0.48871300	-0.24439700	-0.00002000
	C	-0.94305500	1.08525300	-0.00002300
	C	-1.44118900	-1.27512000	-0.00000200
	C	-2.30418900	1.36967100	-0.00000300
	H	-0.22450100	1.90898500	-0.00004200
	C	-2.80527900	-0.99170800	0.00002000
	H	-1.10445900	-2.31672500	-0.00000200
	C	-3.24032200	0.33258300	0.00002100
	H	-2.64023300	2.41034000	-0.00000400
	H	-3.53150100	-1.80928500	0.00003700
	H	-4.31018600	0.55961400	0.00004000

**cyclohexanone**	C	-1.01154900	-1.26151000	-0.28208200
	C	0.39013300	-1.28096100	0.34633600
	C	0.39012300	1.28096300	0.34631800
	C	-1.01157400	1.26150700	-0.28206100
	C	-1.78556700	-0.00001200	0.10611200
	H	0.29206900	-1.36866800	1.44629300
	H	0.99103700	-2.13374300	-0.00562200
	H	-0.91479200	-1.30442000	-1.38287700
	H	-1.56591400	-2.16632000	0.01992100
	H	0.29209400	1.36869200	1.44627500
	H	0.99101500	2.13373800	-0.00567100
	H	-1.56594500	2.16630000	0.01998900
	H	-0.91484700	1.30445500	-1.38285700
	H	-1.96641800	-0.00002400	1.19839700
	H	-2.77737800	-0.00001600	-0.37775000
	C	1.15936700	0.00000300	0.06366400
	O	2.29418500	0.00000900	-0.34572800

**succinimide**	C	-0.76582600	1.25407500	0.00027700
	C	0.76589200	1.25403600	-0.00028400
	H	-1.20813300	1.73930100	-0.88429200
	H	-1.20739100	1.73935700	0.88518400
	H	1.20747600	1.73890900	-0.88542700
	H	1.20821300	1.73965900	0.88408100
	C	-1.17339500	-0.21514800	0.00007700
	C	1.17338400	-0.21526400	0.00004600
	N	-0.00004500	-0.95817900	-0.00005400
	H	-0.00013400	-1.97328100	-0.00001800
	O	-2.28333100	-0.67142300	-0.00010500
	O	2.28332500	-0.67143800	0.00012500

REFERENCES AND NOTES

1. H. Abu Ali, V. M. Dembitsky, M. Srebnik, Chemistry of the diboron compounds. *Stud. Inorg. Chem.* **22**, 1–57 (2005).

2. E. C. Neeve, S. J. Geier, I. A. I. Mkhalid, S. A. Westcott, T. B. Marder, Diboron(4) compounds: From structural curiosity to synthetic workhorse. *Chem. Rev.* **116**, 9091–9161 (2016).

3. E. Fernández, Ed., *Science of Synthesis Reference Library: Advances in Organoboron Chemistry Toward Organic Synthesis* (Thieme, 2020).

4. H. Braunschweig, R. D. Dewhurst, S. Mozo, Building electron-precise boron-boron single bonds: Imposing monogamy on a promiscuous element. *ChemCatChem* **7**, 1630–1638 (2015).

5. M. Arrowsmith, H. Braunschweig, T. E. Stennett, Formation and reactivity of electron-precise B−B single and multiple bonds. *Angew. Chem. Int. Ed. Engl.* **56**, 96–115 (2017).

6. L. Souillart, N. Cramer, Catalytic C–C bond activations via oxidative addition to transition metals. *Chem. Rev.* **115**, 9410–9464 (2015).

7. M. Murakami, N. Ishida, Potential of metal-catalyzed C–C single bond cleavage for organic synthesis. *J. Am. Chem. Soc.* **138**, 13759–13769 (2016).

8. F. Song, B. Wang, Z.-J. Shi, Transition-metal-catalyzed C-C bond formation from C-C activation. *Acc. Chem. Res.* **56**, 2867–2886 (2023).

9. P. Ceron, A. Finch, J. Frey, J. Kerrigan, T. Parsons, G. Urry, H. I. Schlesinger, Diboron tetrachloride and tetrafluoride as reagents for the synthesis of organoboron compounds. II. The behavior of the diboron tetrahalides toward unsaturated organic compounds. *J. Am. Chem. Soc.* **81**, 6368–6371 (1959).

10. J. A. Morrison, Chemistry of the polyhedral boron halides and the diboron tetrahalides. *Chem. Rev.* **91**, 35–48 (1991).

11. R. D. Dewhurst, E. C. Neeve, H. Braunschweig, T. B. Marder, sp^2–sp^3 Diboranes: Astounding structural variability and mild sources of nucleophilic boron for organic synthesis. *Chem. Commun.* **51**, 9594–9607 (2015).

12. J. W. B. Fyfe, A. J. B. Watson, Recent developments in organoboron chemistry: Old dogs, new tricks. *Chem* **3**, 31–55 (2017).

13. W. Biffar, H. Nöth, H. Pommerening, B. Wrackmeyer, Kernresonanzspektroskopische Untersuchungen an Bor-Verbindungen, XVII. ^{17}O-NMR-Studien an organyloxyboranen, organyloxydi-boranen(4), Dioxaborolanen und Boroxinen. *Chem. Ber.* **113**, 333–341 (1980).

14. T. Ishiyama, M. Murata, T.-A. Ahiko, N. Miyaura, BIS(pinacolato)diboron. *Organic Synth.* **77**, 176–185 (2000).

15. C. N. Welch, S. G. Shore, Boron heterocycles. V. Preparation and characterization of selected heteronuclear diboron ring systems. *Inorg. Chem.* **7**, 225–230 (1968).

16. J. Takaya, N. Iwasawa, Catalytic, direct synthesis of bis(boronate) compounds. *ACS Catal.* **2**, 1993–2006 (2012).

17. J. Yun, Copper(I)-catalyzed boron addition reactions of alkynes with diboron reagents. *Asian J. Org. Chem.* **2**, 1016–1025 (2013).

18. F. Zhao, X. Jia, P. Li, J. Zhao, Y. Zhou, J. Wang, H. Liu, Catalytic and catalyst-free diboration of alkynes. *Org. Chem. Front.* **4**, 2235–2255 (2017).

19. D. Hemming, R. Fritzemeier, S. A. Westcott, W. L. Santos, P. G. Steel, Copper-boryl mediated organic synthesis. *Chem. Soc. Rev.* **47**, 7477–7494 (2018).

20. X. Wang, Y. Wang, W. Huang, C. Xia, L. Wu, Direct synthesis of multi(boronate) esters from alkenes and alkynes via hydroboration and boration reactions. *ACS Catal.* **11**, 1–18 (2021).

21. P. Paetzold, B. Redenz-Stormanns, R. Boese, Boroboration of CO with tri-*tert*-butylazadiboriridine. *Angew. Chem. Int. Ed. Engl.* **29**, 900–902 (1990).

22. J. Teichmann, H. Stock, H. Pritzkow, W. Siebert, Carbon monoxide and isonitrile insertion into the B−B bond of five-membered cyclic organo-1,2-diborane. *Eur. J. Inorg. Chem.* **1998**, 459–463 (1998).

23. H. Asakawa, K.-H. Lee, Z. Lin, M. Yamashita, Facile scission of isonitrile carbon–nitrogen triple bond using a diborane(4) reagent. *Nat. Commun.* **5**, 4245 (2014).

24. Y. Katsuma, N. Tsukahara, L. Wu, Z. Lin, M. Yamashita, Reaction of B_2(*o*-tol)$_4$ with CO and isocyanides: Cleavage of the C≡O triple bond and direct C−H borylations. *Angew. Chem. Int. Ed. Engl.* **57**, 6109–6114 (2018).

25. E. Beck, I. Krummenacher, T. Kupfer, M. Dietz, M. Michel, K. Hammond, H. Braunschweig, Structural stability of a bent tetra(amino)tetraborane ring across four charge states. *Chem* **11**, 102338 (2025).

26. E. Beck, D. Bröllos, I. Krummenacher, T. Kupfer, M. Dietz, T. Wellnitz, C. Mihm, H. Braunschweig, Boron-chalcogen heterocycles and linear tetraboranes from a cyclic tetra(amino)tetraborane. *Nat. Commun.* **16**, 5304 (2025).

27. Z. He, A. Zajdlik, A. K. Yudin, Air- and moisture-stable amphoteric molecules: Enabling reagents in synthesis. *Acc. Chem. Res.* **47**, 1029–1040 (2014).

28. J. D. St. Denis, Z. He, A. K. Yudin, Amphoteric α-boryl aldehyde linchpins in the synthesis of heterocycles. *ACS Catal.* **5**, 5373–5379 (2015).

29. H. Noda, J. W. Bode, Synthesis and reactivities of monofluoro acylboronates in chemoselective amide bond forming ligation with hydroxylamines. *Org. Biomol. Chem.* **14**, 16–20 (2016).

30. F. K. Scharnagl, S. K. Bose, T. B. Marder, Acylboranes: Synthetic strategies and applications. *Org. Biomol. Chem.* **15**, 1738–1752 (2017).

31. F. H. Allen, O. Kennard, D. G. Watson, L. Brammer, A. G. Orpen, R. Taylor, Tables of bond lengths determined by X-ray and neutron diffraction. Part 1. Bond lengths in organic compounds. *J. Chem. Soc. Perkin Trans.* **2**, S1–S19 (1987).

32. D. S. Yufit, J. A. K. Howard, Cyclopentanone and cyclobutanone. *Acta Cryst.* **67**, o104–o106 (2011).

33. T. Xu, E. J. Munson, J. F. Haw, Toward a systematic chemistry of organic reactions in zeolites: In situ NMR studies of ketones. *J. Am. Chem. Soc.* **116**, 1962–1972 (1994).

34. M. Arrowsmith, J. Böhnke, H. Braunschweig, M. A. Celik, Reactivity of a Dihydrodiborene with CO: Coordination, insertion, cleavage, and spontaneous formation of a cyclic alkyne. *Angew. Chem. Int. Ed. Engl.* **56**, 14287–14292 (2017).

35. A. Ricci, M. Fiorenza, A. Degl'Innocenti, G. Seconi, P. Dembech, K. Witzgall, H. J. Bestmann, Acylsilane durch oxidation von phosphoniumyliden; erste Synthese von Bis(trimethylsilyl)keton und dessen Anwendung als $CO^{2}\ominus$-Äquivalent. *Angew. Chem. Int. Ed. Engl.* **97**, 1068–1069 (1985).

36. R. Johannesen, T. Benneche, Synthesis of trimethylgermyl trimethylsilyl ketone and bis(trimethylgermyl) ketone. *J. Chem. Soc. Perkin Trans.* **1**, 2677–2679 (2000).

37. H. Dahn, P. Péchy, H. J. Bestmann, NMR of terminal oxygen. Part 11. ^{17}O NMR spectra of disilyl ketone and related compounds: A case of strong deshielding by electronic excitation energy. *J. Chem. Soc. Perkin Trans.* **2**, 1497–1498 (1993).

38. C. Saalfrank, F. Fantuzzi, T. Kupfer, B. Ritschel, K. Hammond, I. Krummenacher, R. Bertermann, R. Wirthensohn, M. Finze, P. Schmid, V. Engel, B. Engels, H. Braunschweig, cAAC-stabilized 9,10-diboraanthracenes—Acenes with open-shell singlet biradical ground states. *Angew. Chem. Int. Ed. Engl.* **59**, 19338–19343 (2020).

39. A. Stoy, J. Böhnke, J. O. C. Jiménez-Halla, R. D. Dewhurst, T. Thiess, H. Braunschweig, CO_2 binding and splitting by boron–boron multiple bonds. *Angew. Chem. Int. Ed. Engl.* **57**, 5947–5951 (2018).

40. A. Stoy, M. Härterich, R. D. Dewhurst, J. O. C. Jiménez-Halla, P. Endres, M. Eyßelein, T. Kupfer, A. Deissenberger, T. Thiess, H. Braunschweig, Evidence for borylene carbonyl

(LHB═C═O) and base-stabilized (LHB═O) and base-free oxoborane (RB≡O) intermediates in the reactions of diborenes with CO_2. *J. Am. Chem. Soc.* **144**, 3376–3380 (2022).

41. W. Lu, N. Hensiek, K. Saha, R. D. Dewhurst, M. Härterich, C. Pranckevicius, S. Hagspiel, M. Dietz, I. Krummenacher, H. Braunschweig, Electron-precise dicationic tetra-boranes: Syntheses, structures and rearrangement to an alkylidene borate-borenium zwitterion and a 1,3-azaborinine. *Chem. A Eur. J.* **29**, e202300644 (2023).

42. H. Nöth, H. Pommerening, Hexakis(dimethylamino)*cyclo*-hexaborane, a boron(I) compound without electron deficiency. *Angew. Chem. Int. Ed. Engl.* **19**, 482–483 (1980).

43. G. Linti, D. Loderer, H. Nöth, K. Polborn, W. Rattay, Reactions and structure of electron-precise triborane(5) and tetraborane(6) derivatives. *Chem. Ber.* **127**, 1909–1922 (1994).

44. A. W. Mesbah, A. Berndt, M. Soleimani, W. Massa, K. Harms, Linear- and cyclo-hexa(amino)hexaboranes: New routes to synthesis of B_6 chain and B_6 ring. *Eur. J. Inorg. Chem.* **27**, e202400440 (2024).

45. Y. Shibutani, S. Kusumoto, K. Nozaki, Fully conjugated tetraborylethylene: Selenium mediated C–C double bond formation from diborylcarbenoid. *Chem. Sci.* **15**, 17912–17917. (2024).

46. R. Köster, G. Seidel, R. Boese, Ethyl-substituierte 2,5-dihydro-1,2,5-oxadiborole. *Chem. Ber.* **127**, 2159–2165 (1994).

47. J. L. Bohlen, L. Endres, R. Drescher, K. Radacki, M. Dietz, I. Krummenacher, H. Braunschweig, Boroles from alumoles: Accessing boroles with alkyl-substituted backbones *via* transtrielation. *Chem. Sci.* **14**, 9010–9015 (2023).

48. S. Stoll, A. Schweiger, EasySpin, a comprehensive software package for spectral simulation and analysis in EPR. *J. Magn. Reson.* **178**, 42–55 (2006).

49. I. Noviandri, K. N. Brown, D. S. Fleming, P. T. Gulayas, P. A. Lay, A. F. Masters, L. Phillips, The decamethylferrocenium/decamethylferrocene redox couple: A superior redox standard to the

ferrocenium/ferrocene redox couple for studying solvent effects on the thermodynamics of electron transfer. *J. Phys. Chem. B* **103**, 6713–6722 (1999).

50. D. Savoia, E. Tagliavini, C. Trombini, A. Umani-Ronchi, Active metals from potassium-graphite. Air oxidized nickel graphite as a new selective hydrogenation catalyst. *J. Organomet. Chem.* **46**, 5344–5348 (1981).

51. G. Sheldrick, Crystal structure refinement with *SHELXL*. *Acta Crystallogr.* **71**, 3–8 (2015).

52. G. Sheldrick, A short history of SHELX. *Acta Crystallogr.* **64**, 112–122 (2008).

53. M. J. Frisch, G. W. Trucks, H. B. Schlegel, G. E. Scuseria, M. A. Robb, J. R. Cheeseman, G. Scalmani, V. Barone, G. A. Petersson, H. Nakatsuji, X. Li, M. Caricato, A. V. Marenich, J. Bloino, B. G. Janesko, R. Gomperts, B. Mennucci, H. P. Hratchian, J. V. Ortiz, A. F. Izmaylov, J. L. Sonnenberg, D. Williams-Young, F. Ding, F. Lipparini, F. Egidi, J. Goings, B. Peng, A. Petrone, T. Henderson, D. Ranasinghe, V. G. Zakrzewski, J. Gao, N. Rega, G. Zheng, W. Liang, M. Hada, M. Ehara, K. Toyota, R. Fukuda, J. Hasegawa, M. Ishida, T. Nakajima, Y. Honda, O. Kitao, H. Nakai, T. Vreven, K. Throssell, J. A. Montgomery, Jr., J. E. Peralta, F. Ogliaro, M. J. Bearpark, J. J. Heyd, E. N. Brothers, K. N. Kudin, V. N. Staroverov, T. A. Keith, R. Kobayashi, J. Normand, K. Raghavachari, A. P. Rendell, J. C. Burant, S. S. Iyengar, J. Tomasi, M. Cossi, J. M. Millam, M. Klene, C. Adamo, R. Cammi, J. W. Ochterski, R. L. Martin, K. Morokuma, O. Farkas, J. B. Foresman, D. J. Fox, Gaussian 16, Revision C.01 (Gaussian Inc., 2016).

54. J.-D. Chai, M. Head-Gordon, Long-range corrected hybrid density functionals with damped atom-atom dispersion corrections. *Phys. Chem. Chem. Phys.* **10**, 6615–6620 (2008).

55. F. Weigend, R. Ahlrichs, Balanced basis sets of split valence, triple zeta valence and quadruple zeta valence quality for H to Rn: Design and assessment of accuracy. *Phys. Chem. Chem. Phys.* **7**, 3297–3305 (2005).

56. A. V. Marenich, C. J. Cramer, D. G. Truhlar, Universal solvation model based on solute electron density and on a continuum model of the solvent defined by the bulk dielectric constant and atomic surface tensions. *J. Phys. Chem. B* **113**, 6378–6396 (2009).

57. A. Gilbert, IQmol, version v3.1.3 (2024); www.iqmol.org.

58. H. Braunschweig, R. D. Dewhurst, F. Hupp, M. Nutz, K. Radacki, C. W. Tate, A. Vargas, Q. Ye, Multiple complexation of CO and related ligands to a main-group element. *Nature* **522**, 327–330 (2015).

59. J. Böhnke, H. Braunschweig, T. Dellermann, W. C. Ewing, K. Hammond, T. Kramer, J. O. C. Jimenez-Halla, J. Mies, The synthesis of $B_2(SIDip)_2$ and its reactivity between the diboracumulenic and diborynic extremes. *Angew. Chem. Int. Ed. Engl.* **54**, 13801–13805 (2015).

60. H. Braunschweig, T. Dellermann, R. D. Dewhurst, W. C. Ewing, K. Hammond, J. O. C. Jimenez-Halla, T. Kramer, I. Krummenacher, J. Mies, A. K. Phukan, A. Vargas, Metal-free binding and coupling of carbon monoxide at a boron-boron triple bond. *Nat. Chem.* **5**, 1025–1028 (2013).
